# The ABL-MYC axis controls WIPI1-enhanced autophagy in lifespan extension

**DOI:** 10.1038/s42003-023-05236-9

**Published:** 2023-08-24

**Authors:** Katharina Sporbeck, Maximilian L. Haas, Carmen J. Pastor-Maldonado, David S. Schüssele, Catherine Hunter, Zsuzsanna Takacs, Ana L. Diogo de Oliveira, Mirita Franz-Wachtel, Chara Charsou, Simon G. Pfisterer, Andrea Gubas, Patricia K. Haller, Roland L. Knorr, Manuel Kaulich, Boris Macek, Eeva-Liisa Eskelinen, Anne Simonsen, Tassula Proikas-Cezanne

**Affiliations:** 1 Interfaculty Institute of Cell Biology, Eberhard Karls University Tübingen, D-72076 Tübingen, Germany; 2 International Max Planck Research School ‘From Molecules to Organisms’, Max Planck Institute for Biology and Eberhard Karls University Tübingen, D-72076 Tübingen, Germany; 3 Proteome Center Tübingen, Interfaculty Institute of Cell Biology, Eberhard Karls University Tübingen, D-72076 Tübingen, Germany; 4 Institute of Basic Medical Sciences, University of Oslo, 0372 Oslo, Norway; 5 Centre for Cancer Cell Reprogramming, Institute of Clinical Medicine, University of Oslo, 0316 Oslo, Norway; 6 Institute of Biochemistry II, Frankfurt Cancer Institute, Goethe University Medical School, D-60590 Frankfurt, Germany; 7 Humboldt University of Berlin, Institute of Biology, D-10115 Berlin, Germany; 8 Graduate School and Faculty of Medicine, The University of Tokyo, Tokyo, 113-0033 Japan; 9 International Research Frontiers Initiative, Institute of Innovative Research, Tokyo Institute of Technology, Yokohama, 226-8503 Japan; 10 Department of Biosciences, University of Helsinki, Fl-00790 Helsinki, Finland; 11 Institute of Biomedicine, University of Turku, FI-20520 Turku, Finland; 12 Present Address: Institute of Molecular Biotechnology, A-1030 Vienna, Austria; 13 Present Address: Department of Anatomy, Faculty of Medicine, University of Helsinki, FI-00290 Helsinki, Finland

**Keywords:** Cell biology, Macroautophagy

## Abstract

Human WIPI β-propellers function as PI3P effectors in autophagy, with WIPI4 and WIPI3 being able to link autophagy control by AMPK and TORC1 to the formation of autophagosomes. WIPI1, instead, assists WIPI2 in efficiently recruiting the ATG16L1 complex at the nascent autophagosome, which in turn promotes lipidation of LC3/GABARAP and autophagosome maturation. However, the specific role of WIPI1 and its regulation are unknown. Here, we discovered the ABL-ERK-MYC signalling axis controlling WIPI1. As a result of this signalling, MYC binds to the WIPI1 promoter and represses WIPI1 gene expression. When ABL-ERK-MYC signalling is counteracted, increased WIPI1 gene expression enhances the formation of autophagic membranes capable of migrating through tunnelling nanotubes to neighbouring cells with low autophagic activity. ABL-regulated WIPI1 function is relevant to lifespan control, as ABL deficiency in *C. elegans* increased gene expression of the WIPI1 orthologue ATG-18 and prolonged lifespan in a manner dependent on ATG-18. We propose that WIPI1 acts as an enhancer of autophagy that is physiologically relevant for regulating the level of autophagic activity over the lifespan.

## Abstract (web summary)

The ABL-ERK-MYC signaling axis inhibits WIPI1 gene expression, thereby controlling the autophagy-enhancing effect of WIPI1 in the context of *C. elegans* lifespan control.

## Introduction

Macroautophagy (hereinafter referred to as autophagy)^1^ plays a critical role in controlling the lifespan of eukaryotic organisms by maintaining cellular integrity and breaking down intracellular proteins, lipids, and organelles for recycling purposes^2,3^. Loss of autophagy control and dysfunctional autophagy are closely associated with the development of age-related pathologies, including various types of cancer and neurodegenerative diseases^4–6^. In this context, the complexity of regulatory signalling networks that control the function of autophagy related (ATG) proteins in certain phases of the autophagy process is still insufficiently understood.

In previous studies, we identified four human WIPI proteins (WIPI1 to WIPI4)^7,8^ that fold into 7-bladed β-propellers^7,9–12^, specifically bind phosphoinositides^9,13,14^, and function as PI3P effectors on the nascent autophagosome^8^, also known as the phagophore^15^. Through their specific interactions with critical autophagy regulators, WIPI proteins can perform nonredundant functions in autophagy, with WIPI1 and WIPI2 acting upstream and WIPI3 and WIPI4 acting downstream of LC3/GABARAP lipidation at the phagophore^9^. After autophagy initiation via AMPK activation^16^ and TORC1 inhibition^17,18^, the ULK1 complex^19–21^ promotes activation of the PI3KC3 complex^22^, which produces PI3P and initiates phagophore formation at the omegasome structures of the endoplasmic reticulum (ER)^4,23–25^. Here, WIPI1 and WIPI2 can heterodimerize and act as lipid sensors by specifically binding to this pool of newly produced PI3P and mediating subsequent steps in autophagosome maturation^26,27^. It is believed that, mechanistically, WIPI1 assists WIPI2 in efficiently recruiting the ATG16L1 complex, which in turn promotes lipidation of LC3/GABARAP^9,27,28^. However, cells lacking WIPI1 can still undergo autophagosome formation^9^ since having WIPI2 is sufficient to enable recruitment of the ATG16L1 complex to the phagophore^29^. Therefore, the specific function of WIPI1 and its regulation remain unknown.

Here, we set out to identify upstream regulators of WIPI1 and performed an automated, image-based, high-throughput screening that targeted human kinases with lentiviral-delivered small hairpin RNAs (shRNAs) while assessing for the presence of WIPI1-decorated autophagic membranes, hereinafter referred to as WIPI1 puncta^30^. We identified ABL1, previously reported to control late stages of autophagy by regulating lysosomal acquisition of hydrolytic enzymes^31^, as a novel inhibitor of WIPI1 puncta formation and confirmed our finding by subsequent quantitative autophagy assessments using WIPI1, WIPI2, LC3/GABARAP^32,33^ and p62^34,35^. Furthermore, stable isotope labeling by amino acids in cell culture (SILAC)-based quantitative phosphoproteomics suggested the possibility that WIPI1 might be under the control of an ABL-ERK2-MYC axis^36,37^, an idea confirmed by (i) an unbiased approach using human autophagy pathway-focused gene expression profiling that revealed WIPI1 gene expression under the control of ABL and (ii) a targeted approach in which we showed that ERK-controlled MYC binds to the WIPI1 promoter and represses WIPI1 mRNA synthesis. To investigate the physiological relevance of ABL1-mediated gene expression control of WIPI1, we used *C. elegans* as a model organism and found that ABL deficiency promotes gene expression of ATG-18, the orthologue of WIPI1, and promotes autophagic flux and lifespan extension. To determine whether elevated WIPI1 protein levels can affect autophagy, we overexpressed WIPI1 in human cells and found evidence that WIPI1 can enhance phagophore formation, as determined by correlative light electron microscopy (CLEM) and by analysis of WIPI2 and LC3. In this context, we discovered that autophagic membranes positive for WIPI1, WIPI2, or LC3 are present in tunnelling nanotubes (TNTs), which are bridges for intercellular transport^38^, and we discovered that autophagic membranes are transported through TNTs to cells lacking sufficient autophagy, as assessed by coculture setups with ATG16 L-deficient human cells^39^.

## Results

### Human kinome screening revealed an inhibitory role for ABL kinases and DDR1 in initiating autophagy

**Fig. 1:**
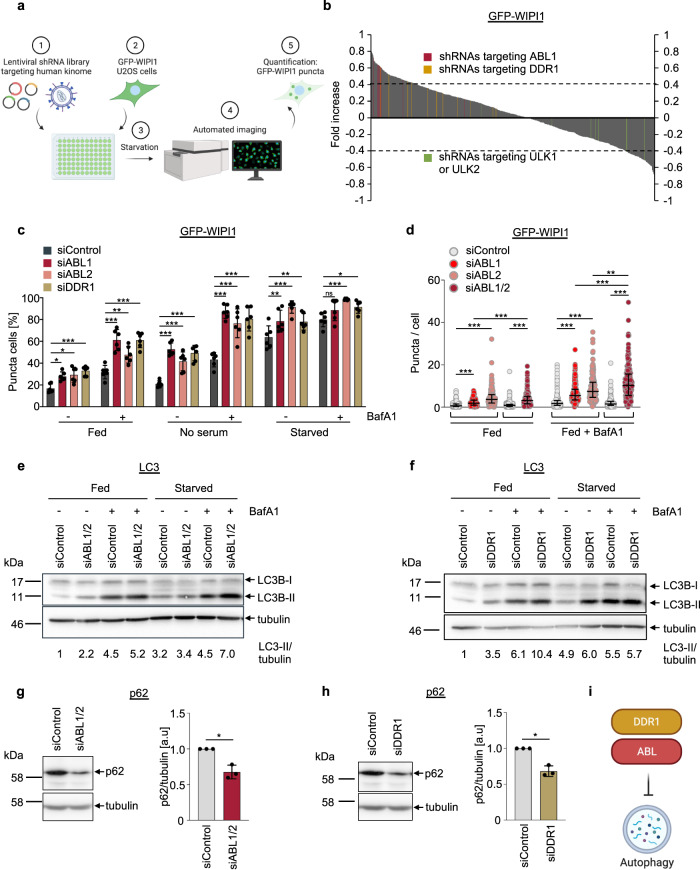
Lentiviral-based GFP-WIPI1 image-based kinome screen reveals a role for ABL kinases and DDR1 in the control of autophagy. **a** U2OS cells stably expressing GFP-WIPI1 were seeded in 96-well plates containing the shRNA library followed by 3 h of starvation, fixation and automated GFP-WIPI1 puncta image acquisition and analysis using the InCell Analyzer 1000. Created with BioRender.com. **b** The difference in the fold increase in GFP-WIPI1 puncta cells after transfection with 1103 single target shRNAs compared to control shRNAs is shown. All shRNAs targeting ABL1 (red), DDR1 (yellow) or ULK1/2 (green) are highlighted. Kinases with a difference in fold increase >0.4 were considered candidates. **c** U2OS cells stably expressing GFP-WIPI1 were transfected with siRNAs targeting ABL1 (siABL1), ABL2 (siABL2), DDR1 (siDDR1) or nontargeting control siRNAs (siControl) for 48 h. Prior to automated GFP-WIPI1 image acquisition and analysis using the InCell Analyzer 1000, cells were incubated in control medium (fed), control medium lacking serum (no serum) or starved for 3 h in the presence (+) or absence (−) of bafilomycin A1 (BafA1). The percentages of GFP-WIPI1 puncta cells are displayed. Puncta were defined by automated threshold-based segmentation in terms of intensity and size of the puncta. Two-way ANOVA with Dunnett’s post-hoc test, mean ± SD, up to 2758 analysed cells from *n* = 6 for each condition. Representative images are shown in Supplementary Fig. 2c. **d** Using CellProfiler-based single cell analysis of images acquired with automated confocal laser-scanning microscopy (LSM), the number of GFP-WIPI1 puncta per cell (threshold-based puncta segmentation) was determined with single knockdown of either ABL1 or ABL2, or double knockdown of ABL1/2 in fed conditions in the presence or absence of BafA1 as indicated. A two-way ANOVA with Tukey’s multiple comparisons test was performed (up to 1241 cells from *n* = 3 for each condition) and error bars show the mean ± SD deviation. **e**, **f** U2OS cells were transfected with siRNAs targeting ABL1/2 (siABL1/2), DDR1 (siDDR1) or nontargeting control siRNAs (siControl) as indicated for 48 h. Protein extracts were analysed by immunoblotting against LC3B and tubulin, and a representative result is shown (*n* = 3, additional immunoblots presented in Supplementary Fig. 2g, i). Numbers indicate LC3-II/tubulin ratios [a.u.]. **g**, **h** Likewise, protein extracts were analysed by immunoblotting against p62 and tubulin (left panels) and relative quantification of p62 protein abundance normalized over tubulin is presented (right panels). Welch’s *t* test, mean ± SD, *n* = 3. **i** The kinome screening results with subsequent confirmation are summarized graphically. Created with BioRender.com. Supplementary material is available (Supplementary Figs. 1, 2; Supplementary Data 1). *P* values: *p < 0.05; **p < 0.01; ***p < 0.001; ns not significant.

In the present study, our primary goal was to define the function of WIPI1 in autophagy, and we hypothesized that understanding the regulation of WIPI1 should represent a crucial step towards this goal. Therefore, using an automated fluorescence-based imaging platform with human U2OS cells stably expressing green fluorescent protein (GFP)-WIPI1^30^, we screened a lentiviral-based human shRNA library targeting human kinases^9^ under starvation conditions while measuring the translocation of GFP-WIPI1 to autophagic membranes, hereafter referred to as puncta (Fig. 1a). The results from targeting 250 human kinases, each with multiple shRNAs applied individually, enabled us to identify 29 candidate kinases whose knockdown led to a significant increase (Fig. 1b; Supplementary Fig. 1a) and 22 candidate kinases whose knockdown led to a significant decrease in the numbers of WIPI1 puncta cells (Fig. 1b; Supplementary Fig. 1b). Among the candidate kinases identified in this unbiased manner (Fig. 1b; Supplementary Fig. 1a, b), we identified known protein kinases, such as ULK2 and EGFR, that activate or inhibit autophagy, respectively, and that modulated the number of WIPI1 puncta cells as expected^40–42^. Pathway enrichment using all candidate kinases indicated involvement of MAPK signalling pathways (Supplementary Fig. 1c, d), and we decided to further analyse ABL1 and DDR1, as both were shown to be involved in MAPK signalling^43,44^ and because 9 (ABL1) or 12 (DDR1) individually applied shRNAs resulted in a consistent increase in WIPI1 puncta cells (Supplementary Data 1).

First, we used alternative small interfering RNA (siRNA) sources to transiently knockdown (KD) ABL1 or DDR1, and at this point, we included siRNAs targeting ABL2 (Supplementary Fig. 2a, b), an ABL1 paralogue that works in tandem with ABL1^45,46^. It is noted here that shRNAs targeting ABL2 were absent in the initial kinome screen. Further, we extended the treatment conditions from the initial starvation screening to also include fed conditions and serum deprivation, both in the absence and presence of bafilomycin A1 (Fig. 1c; Supplementary Fig. 2c). Automated imaging assessment confirmed that ABL1 KD as well as DDR1 KD significantly increased the number of GFP-WIPI1 puncta cells, and we extended this observation to ABL2 KD (Fig. 1c; Supplementary Fig. 2c). Since ABL1 and ABL2 have been shown to heterodimerize to regulate overlapping cellular processes but also to have distinguishable cellular functions^45^, we compared single ABL1 KD and ABL2 KD settings to double ABL1/2 KD settings in relation to autophagy (Fig. 1d). Using automated single-cell analysis to assess GFP-WIPI1 puncta per cell, we confirmed that both ABL kinases are involved in autophagy regulation and that their influence on autophagy inhibition is stronger when both ABL1/2 are down-regulated (Fig. 1d). Based on this result, which suggests a non-redundant effect of ABL1 and ABL2 on autophagy, we downregulated ABL1 and ABL2 together in follow-up experiments. This is consistent with the approach taken by Pendergast’s study, which shows that the late stages of autophagy control in the course of lysosomal acquisition of hydrolytic enzymes depend on both ABL1 and ABL2^31^.

Second, we employed the bona fide autophagy markers LC3 and GABARAPL1^32,33^, functioning downstream from WIPI1^9^, as well as the autophagic receptor SQSTM1/p62, which targets ubiquitinated cargo for autophagic degradation^34,35^. Automated imaging assessments of GFP-LC3 showed that ABL1 KD, ABL2 KD and DDR1 KD (Supplementary Fig. 2d, e) as well as ABL1/2 KD (Supplementary Fig. 2f), elicited a significant increase in the GFP-LC3 puncta per single cell, which was most evident in the presence of bafilomycin A1 (Supplementary Fig. 2d–f), indicating increased autophagic flux. Alternatively, we also used U2OS cells stably expressing tandem fluorescent-tagged LC3^47^ and found that ABL1/2 KD provoked a significant increase in both autophagosomes and autolysosomes (Supplementary Fig. 2g, left panel), further indicating that autophagic flux is increased when ABL kinases are depleted. However, this elevation was less pronounced in DDR1 KD settings, where we observed an increase in autophagosomes in fed conditions and an increase in autolysosomes in starved conditions (Supplementary Fig. 2g, right panel). For this reason, we also performed Western blotting to assess LC3/GABARAPL1 lipidation and p62 protein abundances. We observed an increase in LC3-II (Fig. 1e; Supplementary Fig. 2h) and GAPARAPL1-II (Supplementary Fig. 2i) in both the absence and presence of bafilomycin A1 in ABL1/2 KD, and in DDR1 KD settings (Fig. 1f; Supplementary Fig. 2j). This evidence of increased autophagic flux in both ABL1/2 KD and DDR1 KD settings was further confirmed by p62 Western blotting, which showed that ABL1/2 KD (Fig. 1g) as well as DDR1 KD (Fig. 1h) produced a significant decrease in p62 protein abundance, indicating increased autophagic degradation. Finally, due to the availability of the dasatinib (Supplementary Fig. 2k) and imatinib (Supplementary Fig. 2l, m), pharmacological inhibitors of ABL^48^, we found that, in agreement with the results described above, ABL inhibition led to a significant increase in both GFP-WIPI1 and endogenous WIPI2 puncta cells (Supplementary Fig. 2k–l). We also used DPH, a small-molecule allosteric activator of ABL^49^, and confirmed its mode of action in U2OS cells by using phosphospecific antibodies for CRKL, a bona fide ABL substrate (Supplementary Fig. 2n). Upon subsequent use of DPH to treat U2OS cells, we found that the number of GFP-WIPI1 puncta per single cell decreased significantly under starved conditions (Supplementary Fig. 2o), further confirming our kinome screening results (Fig. 1i).

### ABL kinases and DDR1 inhibit WIPI1 gene expression

Both ABL and DDR1 have been reported to regulate TORC1^50,51^, and the TORC1-TFEB pathway affects WIPI1 gene expression^52^. Furthermore, it has been found that increased WIPI1 gene expression is a response to autophagy initiation when TORC1 is inactive^53,54^. Therefore, the question arose whether the gene expression of WIPI1 could change depending on the status of ABL kinases and DDR1. Since GFP-WIPI1 homodimerizes with endogenous WIPI1^9^, we first assessed GFP-WIPI1 localization to autophagic membranes (puncta) in the presence or absence of cycloheximide (CHX), an inhibitor of eukaryotic translation, and observed that the increase in GFP-WIPI1 puncta cells evoked by ABL1/2 KD (Supplementary Fig. 3a) and DDR1 KD (Supplementary Fig. 3b) was blunted in the presence of CHX. To assess whether ABL kinases and DDR1 actually have an effect, we approached this point in an unbiased fashion by using a human autophagy pathway-focused gene expression array with 84 genes containing WIPI1 (*n* = 3 in duplicate). Indeed, WIPI1 mRNA was significantly upregulated by 3-fold upon ABL1/2 KD, which we also demonstrated using standard TaqMan assessments (Supplementary Fig. 3c–e). Similarly, DDR1 KD also provoked a significant increase in WIPI1 mRNA abundance, albeit to a lesser extent (Supplementary Fig. 3f–h). In addition, we examined the gene expression of all four WIPI genes, as only WIPI1 was part of the ATG array (Supplementary Fig. 3e, h). In both ABL1/2 KD (Supplementary Fig. 3d) and DDR1 KD (Supplementary Fig. 3g) conditions, additional ATG genes were significantly upregulated, including WIPI4 under ABL1/2 KD conditions (Supplementary Fig. 3e).

### ABL kinases and DDR1 inhibit autophagy initiation via MAPK signalling towards MYC

**Fig. 2:**
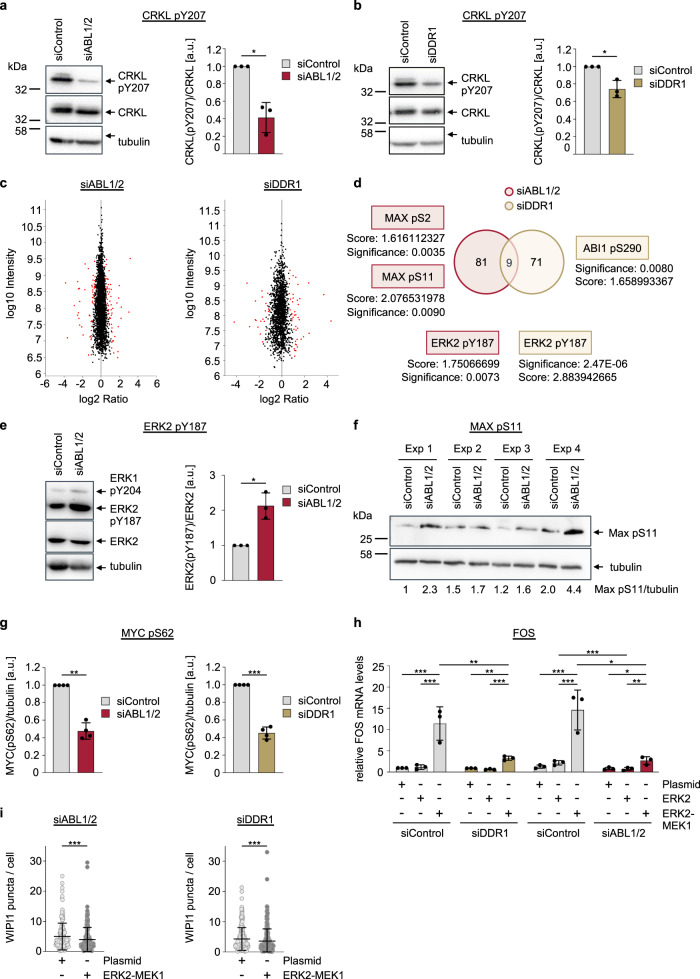
DDR1 and ABL1/2 signal via ERK2-MYC in the control of autophagy. **a**, **b** U2OS cells stably expressing GFP-WIPI1 were transfected with siRNAs targeting ABL1/2 (siABL1/2), DDR1 (siDDR1) or nontargeting control siRNAs (siControl) for 48 h as indicated. Cell extracts were analysed by immunoblotting against CRKL pTyr207, CRKL or tubulin. Representative immunoblots (left panels) and quantifications are presented (right panels). Welch’s *t* test, mean ± SD, *n* = 3. **c** SILAC-based quantitative phosphoproteomics comparing siControl, siABL1/2 and siDDR1 settings. Scatterplot of all phosphosites detected in phospho-SILAC upon downregulation of ABL1/2 (left) or DDR1 (right). Phosphorylation ratios of target siRNA/control siRNA (log(2) versus log(10)) signal intensity are plotted. Significant changes (*p* < 0.05) are highlighted in red. **d** Venn diagram of significantly changed phosphosites along with overlaps. **e** Changes in ERK2 phosphorylation following ABL1/2 knockdown were confirmed by Western blotting. U2OS cells were transfected with siABL1/2 or nontargeting siRNA (siControl) for 48 h, and cell extracts were analysed by immunoblotting against ERK1/2 p-Y204/Y187, ERK2 and tubulin. Quantification of protein abundance was conducted by Welch’s *t* test, mean ± SD, *n* = 3. **f** Confirmation of increased MAX p-Ser11 phosphorylation is displayed (*n* = 4, indicated as Exp 1 through Exp 4). **g** U2OS cells were transfected with siABL1/2 or siDDR1 and the corresponding nontarget control (siControl) for 48 h and then treated with 10 µM MG132 for 3 h to prevent proteasomal MYC degradation. Protein extracts were analysed by immunoblotting against MYC p-Ser62, MYC or tubulin. Welch’s *t* test, mean ± SD, *n* = 4. **h** U2OS cells were treated with siRNAs targeting ABL1/2 or DDR1 for 16 h prior to transfection with empty control plasmids or with plasmids encoding myc-tagged ERK2 or myc-tagged ERK2-MEK1 for 48 h. Total RNA was extracted, and relative FOS gene expression was analysed by qPCR. Two-way ANOVA with Tukey’s multiple comparison’s test, mean ± SD, *n* = 3 in triplicates. **i** Using CellProfiler-based single cell analysis of images acquired with automated confocal laser-scanning microscopy (LSM), the number of GFP-WIPI1 puncta per cell (threshold-based puncta segmentation) was determined in ABL1/2 KD (left panel) or DDR1 KD (right panel) U2OS cells overexpressing empty control plasmids or myc-tagged ERK2-MEK1 for 48 h in fed conditions. Welch’s *t* testing was performed (up to 1496 cells from *n* = 3 for each condition) and error bars show the mean ± SD deviation. Supplementary material is available (Supplementary Data 1). *P* values: *p < 0.05; **p < 0.01; ***p < 0.001; ns not significant.

Since we also found additional ATG genes upregulated in the settings of ABL KD (Supplementary Fig. 3c–e, Supplementary Data 1) and DDR1 KD (Supplementary Fig. 3f, g; Supplementary Data 1), we further considered whether the TORC1-TFEB route downstream of ABL and DDR1 could be the reason for our results. To address this question, we decided to perform an unbiased quantitative SILAC-based phospho-proteomics approach, assessing specific and overlapping changes in phosphoproteins of U2OS cells with KD of ABL1/2 and DDR1 (Supplementary Data 1). The rationale for using the KD of ABL1/2 and DDR1 to look for overlapping proteins that showed a changed phospho-status in both KD situations was based on the following initial experiment (Fig. 2a, b). While ABL1/2 KD resulted in the expected significant decrease in phosphorylation of its bona fide target CRKL at Y207 (Fig. 2a), we found that DDR1 KD also resulted in a significant decrease in CRKL phosphorylation (Fig. 2b), suggesting that ABL1/2 and DDR1 could operate on the same signalling axis. When we then turned to the phospho-proteome and compared the ABL1/2 KD and DDR1 KD settings, instead of pointing to the TORC1-TFEB route, our results led us to the ERK signalling axis (Fig. 2c, d; Supplementary Data 1). An increased level of ERK2 phosphorylation at Y187 was revealed in both the ABL1/2 KD and DDR1 KD settings (Fig. 2d; Supplementary Data 1). Y187 is part of the threonine–glutamic acid–tyrosine residue (TEY) motif within the ERK2 activation loop, and activation requires dual phosphorylation at T185 and Y187^55^. Since we observed an unequal phospho-status with more phosphorylation at Y187 but not at T185, ERK2 was in its inactive state, consistent with the established paradigm where ABL and DDR1 activate ERK signalling^43,44^. Notably, we also confirmed a significant increase in ERK2 Y187 phosphorylation by Western blotting in the ABL1/2 KD setting (Fig. 2e) as well as in DDR1 KD settings (Supplementary Fig. 4a).

In agreement with our initial finding concerning CRKL phosphorylation (Fig. 2a, b), our phosphoproteomics analysis further suggested that DDR1 should function upstream of ABL since DDR1 deficiency altered the phospho-status at S290 of ABI1 (Fig. 2d; Supplementary Data 1), an interaction partner and regulator of ABL kinases^56^. Interestingly, in both ABL1/2 KD and DDR1 KD settings, phosphorylation of MAX—a DNA-binding protein that heterodimerizes with MYC to transcriptionally control target gene expression involved in various cellular behaviours ranging from proliferation to lifespan control^57^—at S2 and S11^58^ was significantly altered (Fig. 2d, f; Supplementary Data 1). That this is relevant in this context to the heteromeric function of MYC-MAX downstream of ERK and dependent on ABL and DDR1 was then indicated when we assessed S62 phosphorylation of MYC, a bona fide target site for ERK^37^, required for transcriptional transactivation^59^. We found that both ABL1/2 KD and DDR1 KD (Fig. 2g) significantly decreased the abundance of phospho-S62 MYC, in line with ERK being inactive in this context. That ERK2 is inactive in the absence of ABL1/2 or DDR1 is also shown by employing a construct expressing constitutively active ERK as a myc-ERK2-MEK1 fusion protein^60^ and scoring for FOS gene expression upon ABL1/2 KD and DDR1 KD (Fig. 2h). Consistent with this result, in both ABL1/2 KD and DDR1 KD settings, GFP-WIPI1 puncta counts decreased significantly when the ERK2-MEK1 fusion protein was overexpressed, as shown in automated single-cell image analysis (Fig. 2i).

In addition, although our phospho-proteomics analysis showed no change in the TORC1-TFEB route, we analysed several mTOR targets by quantitative Western blotting, including TFEB, in the settings ABL1/2 KD, DDR1 KD, ERK2 KD and MYC KD (Supplementary Fig. 4b–l). Down-regulation of neither ABL1/2 nor DDR1 altered TFEB phosphorylation at the mTOR target site S211 (Supplementary Fig. 4b), and ABL/DDR1 KD had no additive effect on TFEB pS211 either (Supplementary Fig. 4b). It is also worth noting in this context that ABL1/2 KD and DDR1 KD also had no effect on the mTOR target site ULK1 pS758, which serves as a switch for autophagy initiation, while treatment with the specific mTOR inhibitor Torin 1, as expected, resulted in a significantly reduced level of ULK1 pS758 (Supplementary Fig. 4c, d). Likewise, the phosphostatus of S6K pT389 remained unchanged in the ABL1/2 KD and DDR1 KD settings (Supplementary Fig. 4c, e). In line with these results, downregulation of neither ERK2 nor c-MYC altered the phosphostatus of ULK1 pS758 or S6K pT389 (Supplementary Fig. 4g–k). The significant changes in the ABL-ERK-MYC axis detected by our phosphoproteome analysis are therefore not to be classified in the context of the TORC1 signaling pathway and therefore the following experiment is also to be understood in full agreement with this hypothesis. When we downregulated ABL1/2 or DDR1, we showed that the autophagic flux was increased, as indicated by decreased p62 levels (Fig. 1g, h). Now, when we performed this experiment in the presence of Torin 1, p62 levels decreased significantly further (Supplementary Fig. 4f), suggesting that ABL-ERK-MYC and TORC1 signalling are additive pathways in controlling the autophagic flux. In this context, we confirmed that ABL1/2 and DDR1 do not display an additive effect on p62 degradation (Supplementary Fig. 4l).

### MYC binds to the WIPI1 promoter and represses WIPI1 gene expression

**Fig. 3:**
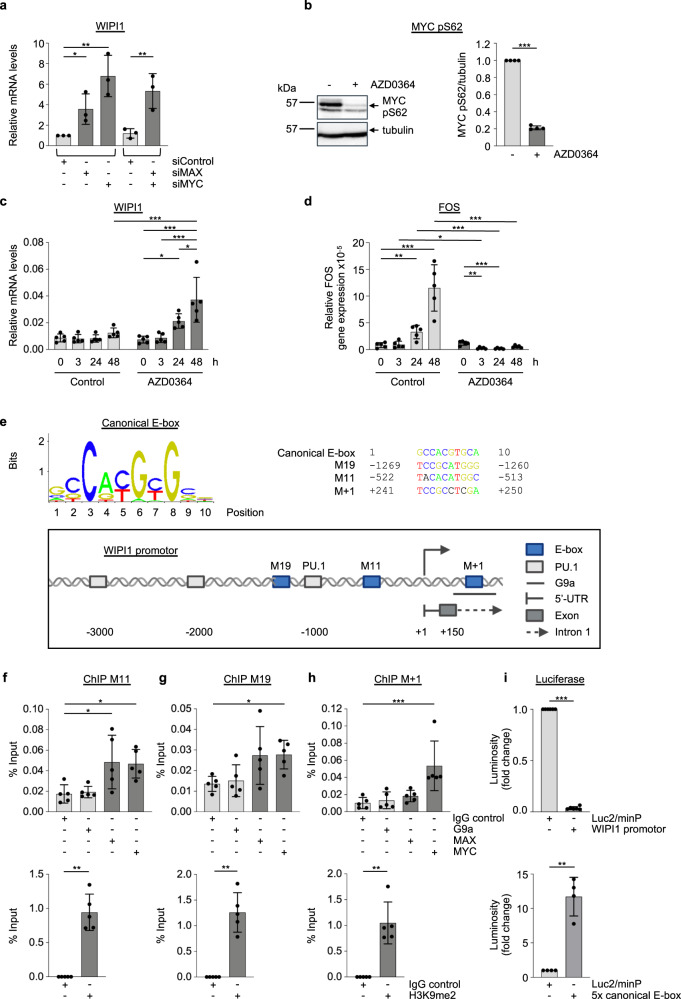
MYC represses WIPI1 gene expression. **a** MAX, MYC or both were transiently downregulated using siRNAs in U2OS cells for 72 h, and total RNA was extracted, followed by assessing WIPI1 mRNA levels by RT‒qPCR. One-way ANOVA with Holm-Sidak post-hoc testing, mean ± SD, *n* = 3. **b** U2OS cells were treated with 10 µM AZD0364 for 48 h, and during the last 3 h, 10 µM MG132 was added to prevent proteasomal MYC degradation. Cell extracts were analysed by immunoblotting against MYC p-Ser62, MYC or tubulin, (left panel: representative Western blot, right panel: quantification). Welch’s t test, mean ± SD, *n* = 4. **c**, **d** U2OS cells were treated with or without 10 µM AZD0364 for 0, 3, 24 or 48 h. Total RNA was extracted, and relative WIPI1 (**c**) or FOS (**d**) gene expression was analysed by RT‒qPCR. Two-way ANOVA with Tukey’s post hoc testing, mean ± SD, *n* = 5. **e** Canonical E-box sequence used to identify potential E-boxes in the human WIPI1 promoter (left upper panel) using ConTra v3. Clustal Omega-based multiple nucleotide sequence alignment using a canonical E-box sequence along with putative E-boxes (M + 1, M11, M19) in the human WIPI1 promoter (upper right panel, indicated are the positions relative to the transcription start site (+1) according to Ensembl Release 109 (Feb 2023), transcript ID ENST00000262139.10 WIPI1-201). A schematic WIPI1 promoter overview with putative E-boxes was created with BioRender.com. Published binding sites for PU.1 are additionally indicated. **f**–**h** U2OS cells were crosslinked, and chromatin immunoprecipitation against G9A, MAX, MYC or negative control IgG was performed. qPCR against M11 (**f**), M19 (**g**) or M + 1 (**h**) binding sites was conducted, the %input was calculated and analysed with one-way ANOVA followed by Dunnett’s post-hoc testing (mean ± SD, *n* = 5) (upper panels). Alternatively, chromatin immunoprecipitation was performed against H2K9me2 (lower panels). Welch’s *t* test, mean ± SD, *n* = 5. **i** Dual-luciferase reporter assays were conducted using U2OS co-transfected for 24 h with pGL4.73[hRluc/SV40] expressing Renilla luciferase together with plasmids with the potential to drive firefly luciferase from a minimal promotor: empty pGL4.23[luc2/minP] plasmid (Luc2/minP), pGL4.23-WIPI1promotor (WIPI1 promotor, upper panel, *n* = 6) or pGL4.23-5xE-box (5x canonical E-box, lower panel, *n* = 4). Firefly- and Renilla luciferase activities were measured via luminosity and firefly luminosity normalized to Renilla luminosity for each sample. For statistical analysis, an unpaired *t*-test with Welch’s correction was performed. Error bars show the mean ± Standard deviation. Supplementary material is available (Supplementary Fig. 4a–c; Supplementary Data 1). *P* values: *p < 0.05; **p < 0.01; ***p < 0.001; ns not significant.

Since unbiased analysis of ATG gene expression in both ABL1/2 KD and DDR1 KD settings revealed that WIPI1 gene expression increased in this context (Supplementary Fig. 3), and our proteomic analysis pointed towards the transcription factor MYC/MAX, we now asked whether MYC-MAX deficiency affects WIPI1 gene expression. Indeed, we were able to show through qPCR assessments that the absence of MYC or MAX significantly increased WIPI1 gene expression (Fig. 3a). Furthermore, we also pursued this question in an unbiased manner using the human autophagy pathway-focused gene expression array with 84 genes containing WIPI1 that we already used for ABL1/2 KD and DDR1 KD settings (Supplementary Fig. 3). Again, WIPI1 mRNA was significantly up-regulated and scored as a positive hit on MYC KD (Supplementary Fig. 5a). Likewise, as in ABL1/2 KD and DDR1 KD settings, additional ATG genes were upregulated after downregulation of MYC (Supplementary Fig. 5a), and in overlap with the ABL1/2 KD phenotype, WIPI4 was upregulated, albeit to a much lesser degree compared to WIPI1 (Supplementary Figs. 3e, 5b). As in the ABL1/2 KD and DDR1 KD settings, WIPI2 and WIPI3 are not significantly upregulated (Supplementary Figs. 3e, h, 5b). Since the upregulation of WIPI1 was an overlapping result after ABL1/2 KD, DDR1 KD and MYC KD (Supplementary Fig. 3, Fig. 3a; Supplementary Fig. 5a, b), and this being most pronounced compared to the other WIPI genes (Supplementary Figs. 3e, h, 5b), we subsequently focused on further characterizing WIPI1 gene expression in the context of the ABL-MYC signalling axis.

In this context, we wanted to assess whether ERK2 inactivation would also increase WIPI1 gene expression. To this end, we treated U2OS cells with AZD0364, an ERK2 inhibitor, which significantly reduced ERK-mediated phosphorylation of S62 in MYC (Fig. 3b), phenocopying the effect of ABL1/2 KD and DDR1 KD on ERK phosphorylation at Y187. Clearly, administration of AZD0364 over time elicited a significant upregulation of WIPI1 mRNA levels, showing that ERK inhibition also provoked an increase in WIPI1 gene expression (Fig. 3c). Furthermore, the AZD0364-mediated increase in WIPI1 gene expression was significantly reduced when c-MYC was simultaneously overexpressed in the siControl, and in siABL1/2 settings the AZD0364-mediated increase in WIPI1 expression was attenuated (Supplementary Fig. 5c). To verify that AZD0364 impacts ERK2-mediated changes in gene expression, we used FOS as a bona fide read-out for ERK-mediated transcription control and demonstrated that ERK inhibition by AZD0364 inhibited FOS gene expression as expected (Fig. 3d).

To investigate further, we next asked if MYC-MAX could directly control WIPI1 gene expression by binding to the WIPI1 promoter. For this purpose, we used the ConTra v3 webserver^61^ to predict E-boxes (Fig. 3e; Supplementary Fig. 5d-f), classic MYC-MAX binding sites^62^ (Fig. 3e, upper left panel) in the human WIPI1 promoter and identified candidate E-boxes termed M19, M11 and M + 1 hereafter (Fig. 3e, upper right panel, lower panel). In this context, we recognized that M + 1 is positioned at a specific site in the WIPI1 promoter previously shown to be occupied by histone methyltransferase G9a/EHMT2^63^ (Fig. 3e, lower panel). Since the heterodimer MYC-MAX interacts with G9a to repress transcription of target genes^64^, we tested whether MYC could bind to the predicted E-boxes (M11, M19) as well to the region occupied by G9a (M + 1) in the WIPI1 promoter by conducting chromatin immunoprecipitation (ChIP). In addition, we performed ChIPs with anti-H3K9me2 antibodies as repressive marks since MYC-MAX in complex with G9a regulates H3K9me2 dimethylation^64^. This evaluation revealed that MYC and MAX bind to the WIPI1 promoter at the M11 E-box (Fig. 3f, upper panel) and MYC further to the M19 E-box (Fig. 3g, upper panel), located upstream of the transcription initiation site (+1) (Fig. 3e, lower panel), marked as repressive sites by H3K9me2 occupancy (Fig. 3f, g; lower panels). Likewise, MYC could also bind to the M + 1 E-box in the WIPI1 promoter downstream of the transcription initiation site (Fig. 3h, e; lower panel). Based on these results, we performed luciferase reporter assays and confirmed that a partial WIPI1 promoter sequence, including E-boxes M19, M11, and M + 1 (Supplementary Fig. 5g), represses the minimal promoter activity driving firefly luciferase (Fig. 3i, upper panel). As a positive control for MYC-mediated activation, we used a sequence with five canonical E-boxes (Supplementary Fig. 5h), which activated minimal promoter activity on firefly luciferase (Fig. 3i, lower panel). Our analysis is consistent with the result from a global genome mapping approach that found that MYC can bind to the WIPI1 promoter, although this interaction had not yet been further characterized at the time^65^. Our results further support the idea that MYC represses WIPI1 gene expression, and when we then performed a WIPI1/MYC gene expression correlation meta-analysis with Genevestigator, a large-scale microarray reference database and analysis tool, we indeed found that high MYC expression correlates with low WIPI1 gene expression in various human cell lines (Supplementary Fig. 5i).

### Elevated WIPI1 protein levels enhance autophagy initiation across cell boundaries

**Fig. 4:**
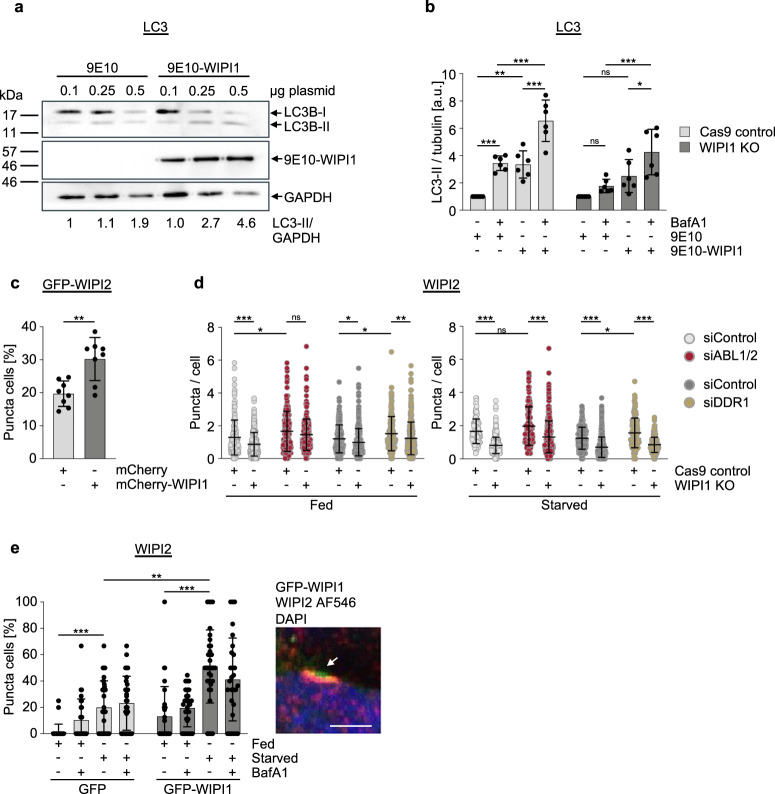
Increase of WIPI1 protein enhances autophagic flux. **a** Immunoblot analysis of LC3B lipidation in U2OS cells after transfection with control plasmids (9E10) or plasmids encoding 9E10-tagged WIPI1. U2OS cells were transfected with the indicated amounts of plasmids for 48 h before protein extraction and immunoblotting against LC3B, 9E10-tagged WIPI1 or GAPDH, *n* = 3. Additional immunoblots provided in Supplementary Fig. 5a. **b** U2OS Cas9 control or U2OS WIPI1 KO cells were transfected with control plasmids (9E10) or plasmids encoding 9E10-tagged WIPI1 for 48 h, followed by treatment with bafilomycin A1 (BafA1) in fed conditions for 3 h. Immunoblotting was conducted against LC3B, 9E10 and tubulin (*n* = 6, mean ± SD, Two-way ANOVA with Tukey’s multiple comparisons test). WIPI1 deficiency control Taqman qPCR is presented in Supplementary Fig. 6b, and representative Western blots in Supplementary Fig. 6c. **c** U2OS cells stably expressing GFP-WIPI2 were transfected with control plasmids or plasmids encoding mCherry-tagged WIPI1 for 48 h. The numbers of GFP-WIPI2 puncta cells were assessed by fluorescence microscopy in transfected cells. Welch’s t test, mean ± SD, up to 1272 analysed cells from *n* = 4 in duplicates. **d** U2OS Cas9 control or U2-OS WIPI1-KO cells were seeded into 96-well glass bottom plates and transfected with siABL1/2, siDDR1 or nontargeting siRNA (siControl) for 48 h, followed by treatment with either DMEM/FBS (fed) or EBSS (starved) for 3 h. After fixation, cells were stained with DAPI and anti-WIPI2/AF488. By automated confocal LSM, 20 images per well were acquired and between 621 to 2563 single cells (from *n* = 3) subjected to automated CellProfiler-based image analysis (threshold-based puncta segmentation). For statistical analysis, a two-way ANOVA with Tukey’s multiple comparisons test was performed (mean ± SD). **e** G361 cells were transfected with plasmids encoding GFP or GFP-WIPI1 and were fed or starved for 3 h in the presence or absence of bafilomycin A1, followed by anti-WIPI2/AF546 immunofluorescence staining. Confocal LSM stacks were acquired, and the numbers of WIPI2 puncta-positive cells per acquired image (individual data points represent the result derived from each image) were counted (left panel: two-way ANOVA with Tukey’s post-hoc test, mean ± SD, up to 215 single cells from *n* = 3 for each condition). Indicative of the presence of overexpressed GFP-WIPI1 are elongated, perinuclear autophagic membranes found to colocalize with WIPI2 (right panel: Scale bar: 5 μm, extended image presentation in Supplementary Fig. 5b). Supplementary material is available (Supplementary Fig. 6d; Supplementary Data 1). *P* values: *p < 0.05; **p < 0.01; ***p < 0.001; ns not significant.

**Fig. 5:**
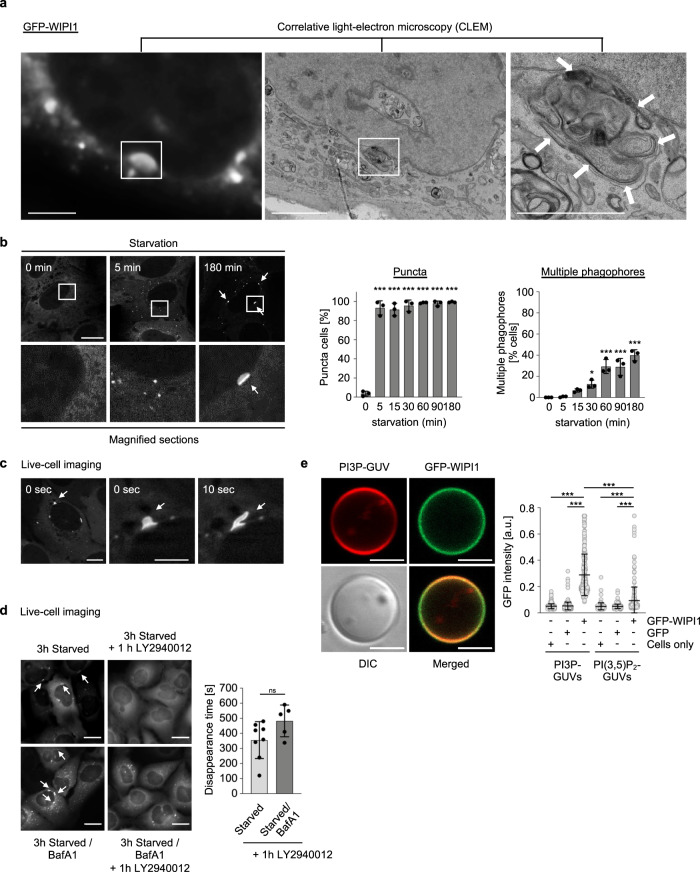
Elevation of WIPI1 protein abundance enhances phagophore formation. **a** Correlative light (left panel) electron microscopy (middle, right panel) of U2OS cells stably expressing GFP-WIPI1. Elongated, perinuclear autophagic membranes decorated with WIPI1 (left panel) were found to represent the presence of multiple phagophore-like membranes (indicated with white arrows, right panel). Scale bars: 20 μm (left panel), 5 μm (middle panel), 2 μm (right panel). **b** Time-course analyses of WIPI1 puncta formation after transient inhibition of PI3P synthesis. Stable U2OS GFP-WIPI1 cells were pretreated with LY294002 (100 µM) for 3 h, followed by starvation for 5, 15, 30, 60, 90 min as indicated. Representative images were acquired by confocal LSM (scale bar: 20 µm). The numbers of WIPI1 puncta cells (middle panel) and the numbers of cells harbouring elongated, perinuclear autophagic membranes decorated with GFP-WIPI1 (right panel) were quantified by fluorescence microscopy (one-way ANOVA with Dunnett’s post-hoc test, mean ± SD, 300 cells from *n* = 3). **c** Stable U2OS GFP-WIPI1 cells were starved and analysed by live-cell microscopy. Still images from Supplementary Video 1 displaying omegasome-like structures decorated with GFP-WIPI1 are displayed. **d** GFP-WIPI1-positive phagophores are sensitive to the inhibition of PI3P synthesis. U2OS cells stably expressing GFP-WIPI1 were starved with or without bafilomycin A1 (BafA1) and subjected to live-cell microscopy. Approximately 3 h after autophagy induction by starvation, LY294002 (100 µM) was added. The disappearance of WIPI1-positive perinuclear phagophores after LY294002 treatment was determined by live-cell microscopy and representative still images are shown (left panels, scale bar: 20 µm). Quantifications are provided in the right panels (Welch’s *t* test, mean ± SD, *n* = 8 videos for starved conditions, *n* = 5 videos for starved/BafA1 conditions). **e** Rhodamine-PE GUVs containing PI(3)P (representative images, left panels, scale bar: 5 µm) or PI(3,5)P_2_ (images shown with controls in Supplementary Fig. 7) were incubated with native protein extracts from U2OS cells stably expressing GFP-WIPI1 or GFP, along with parental U2OS cells, followed by confocal LSM imaging. CellProfiler-based image analysis was used to measure the GFP intensity on GUV edges (right panels, Kruskal–Wallis with Dunn’s post-hoc testing, up to 404 GUVs analysed from *n* = 3 for each condition). Supplementary material is available (Supplementary Fig. 7; Supplementary Video 1; Supplementary Data 1). *P* values: *p < 0.05; **p < 0.01; ***p < 0.001; ns not significant.

Combined, our results obtained up to this point provided evidence that the ABL-ERK-MYC axis negatively controls WIPI1 gene expression and that DDR1 may act upstream of ABL in this context. We committed at this stage to analyse the potential signalling pathway from DDR1 to ABL with an additional approach and here to focus more on effects resulting from increased WIPI1 levels. Because WIPI1, in contrast to WIPI2, is expressed at extremely low levels^30^, we transiently overexpressed 9E10-tagged WIPI1 using increasing concentrations of plasmids and observed that an increased level of overexpressed WIPI1 correlated with an increase in lipidated LC3 (Fig. 4a; Supplementary Fig. 6a). Consistent with this result, quantification of LC3 lipidation in fed conditions in the presence or absence of lysosomal inhibitor upon overexpression of 9E10-WIPI1 demonstrated significantly increased autophagic flux (Fig. 4b, Cas9 control). The autophagic flux was less pronounced in WIPI1 KO cells (Fig. 4b; Supplementary Fig. 6b, c) as seen under fed conditions comparing minus and plus BafA1 settings, when overexpressing the empty 9E10 vector control (Fig. 4b). However, this deficiency was rescued by overexpression of 9E10-WIPI1 (Fig. 4b). Likewise, we also found that the number of GFP-WIPI2B puncta cells increased significantly when mCherry-WIPI1 was overexpressed (Fig. 4c), and conversely, that the absence of WIPI1 decreased the number of endogenous WIPI2 puncta in both ABL1/2 KD and DDR1 KD settings, as shown by automated single-cell image analysis (Fig. 4d). As WIPI1 is the interaction partner of WIPI2 and is expected to support WIPI2-mediated LC3 lipidation, we have detailed the WIPI2 response in terms of its localization to autophagic membranes. To this end, we overexpressed GFP-WIPI1 under both fed and starved conditions and in the absence or presence of bafilomycin A1 and immunostained endogenous WIPI2. Again, we observed that an increase in WIPI1 protein caused an increase in WIPI2 puncta cells, particularly under starved conditions (Fig. 4e, left panel; Supplementary Fig. 6d). In this context, we observed that typical WIPI1 structures, elongated puncta mainly in the perinuclear region^7^, became visible for WIPI2 only when WIPI1 was overexpressed (Fig. 4e, right panel; Supplementary Fig. 6d), suggesting that WIPI1 can promote WIPI2 localization towards autophagic membranes. If this is the case, we hypothesized that treatments that counteract the ABL-ERK-MYC axis and induce WIPI1 gene expression should also affect WIPI2 localization to autophagic membranes. Indeed, both ABL inhibition by the pharmacologically selective inhibitor dasatinib and MYC inhibition by 10058-F4 or 10074-G5, specific compounds that prevent MYC-MAX heterodimerization and inhibit the regulation of MYC target genes, resulted in an increase in WIPI2 puncta cells (Supplementary Fig. 2k). Likewise, ERK inhibition by AZD0364 resulted in an increase in WIPI2 puncta over time (Supplementary Fig. 6e) as well as an increased p62 degradation (Supplementary Fig. 6f), a response that correlates with an increase in WIPI1 gene expression (Fig. 3c) and that was blunted by inhibiting gene expression using CHX (Supplementary Fig. 3a, b). These assessments suggest that increasing WIPI1 levels can increase autophagy levels to a certain extent. This idea was further supported by correlative light electron microscopy observations, which revealed that perinuclear, elongated WIPI1 puncta represent multiple formed phagophores at the same site (Fig. 5a). Moreover, such multiple phagophore production sites were found to develop with prolonged starvation (Fig. 5b). Since multiple phagophore formation sites should be associated with omegasomes, we evaluated GFP-WIPI1 puncta by live cell microscopy and indeed showed that elongated GFP-WIPI1 puncta are highly dynamic and resemble omegasomes (Fig. 5c, Supplementary Video 1), at which WIPI2 is located, as previously shown^27^. Next, we confirmed that elongated GFP-WIPI1 puncta are not a result of a blockage downstream in the pathway of forming productive autophagosomes by performing the following live-cell microscopy experiment. First, we starved cells and then treated these starved cells with the PI3P inhibitor LY2940002, resulting in cells lacking any GFP-WIPI1 puncta (Fig. 5d, left upper panels). Importantly, we conducted the same experiment but in the presence of bafilomycin A1, blocking the autophagic pathway at the lysosome level (Fig. 5d, left lower panels). This demonstrated that elongated GFP-WIPI1 puncta (marked with arrows) disappeared in starved cells that were first treated with bafilomycin A1 and afterwards with the PI3P inhibitor LY2940002, indicating that elongated GFP-WIPI1 puncta formation is the result of newly formed autophagic membranes and not the result of a block in autophagic flux. This idea was further supported by measuring the disappearance time of perinuclear elongated GFP-WIPI1 puncta in cells treated with LY2940002 after starvation (3 h) in the presence or absence of bafilomycin, with the disappearance time being similar in both conditions (Fig. 5d, right panel). Finally, we confirmed that GFP-WIPI1 preferentially binds to PI3P over PI(3,5)P_2_^9,14^ by generating giant unilamellar vesicles (GUVs) containing either PI3P or PI(3,5)P_2_ and then quantifying the binding efficiency of GFP-WIPI1 derived from native cell extracts (Fig. 5e; Supplementary Fig. 7a–c). Taken together, these data suggest that WIPI1 should act as an enhancer of autophagy.

**Fig. 6:**
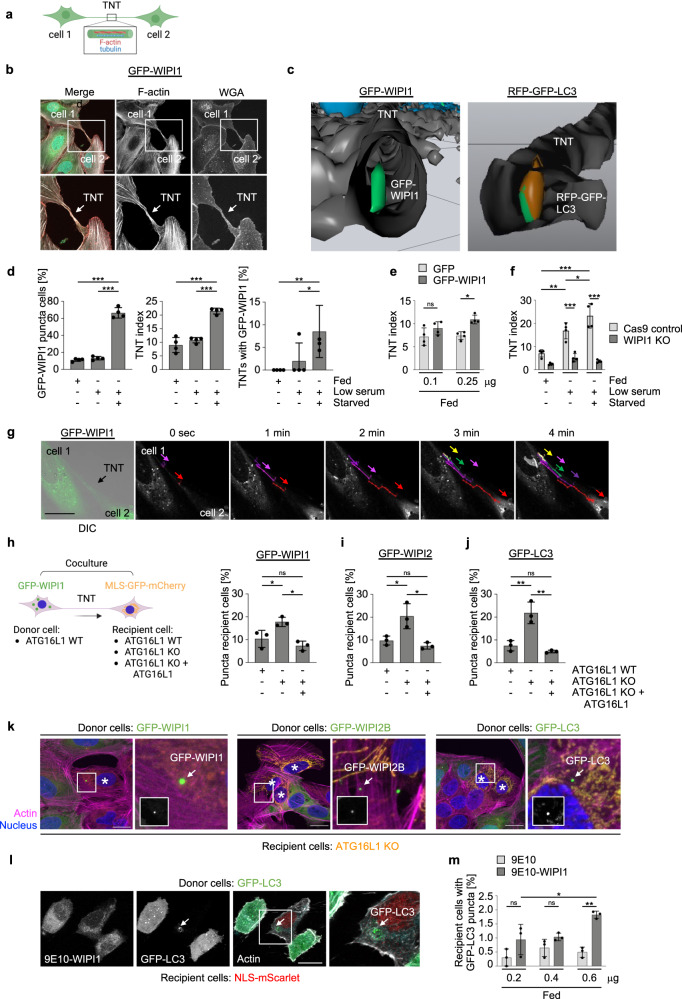
WIPI1-, WIPI2- and LC3-decorated autophagic membranes migrate through tunnelling nanotubes (TNTs) to neighbouring cells with limited autophagic activity. **a** Scheme displaying a TNT connecting two cells. Created with BioRender.com. **b** U2OS cells stably expressing GFP-WIPI1 form TNTs. U2OS GFP-WIPI1 cells were costained with phalloidin-AF546 to visualize F-actin and with WGA-AF647 to visualize the plasma membrane. Merged channels (left panels) are shown as well as split channels displaying F-actin (middle panels) and WGA only (right panels). Boxes (upper panels) indicate magnified sections (lower panels), and arrows point at a TNT connecting two cells. Scale bar: 20 µm. **c** Starved (24 h) U2OS cells stably expressing GFP-WIPI1 were stained with phalloidin-AF546 and DAPI and imaged by confocal LSM. A video was generated from the 3D reconstruction of confocal z-stacks (Supplementary Video 2), and a still image displaying GFP-WIPI1 puncta within a TNT is presented (left panel). Likewise, a video was generated from the 3D reconstruction of confocal z-stacks (Supplementary Video 3), and a still image revealing the presence of RFP-GFP-LC3 puncta within a TNT is shown (right panel). **d** U2OS GFP-WIPI1 cells were fed for 24 h with high (10%, fed) or low (2.5%, low serum) serum or starved as indicated. GFP-WIPI1 puncta-positive cells were counted (left panel), as well as the numbers of TNTs per 100 cells (TNT index, middle panel) and the numbers (%) of TNTs containing GFP-WIPI1 puncta (right panel). One-way ANOVA with Holm-Sidak post-hoc test, up to 952 cells from *n* = 4 for each condition. **e** U2OS cells were transfected with plasmids encoding GFP or GFP-WIPI1 in the concentrations of 0.1 μg or 0.25 μg for 48 h, then re-seeded onto coverslips and cultured for 24 h in fed conditions. After fixation, cells were stained with DAPI, and F-actin stained with phalloidin-AF546 and tubulin with anti-αTubulin/AF647. The TNT index was calculated from up to 1057 cells per condition (*n* = 4). Two-way ANOVA, Tukey’s post-hoc test. Scale bar: 20 μm. **f** U2OS Cas9 control and U2OS WIPI1 KO were seeded onto coverslips and cultured overnight (16 h) and then fed for 24 h with high (10%, fed) or low (2.5%, low serum) serum or starved as indicated. Cells were stained with DAPI, WGA-AF488, phalloidin-AF546, anti-αTubulin/AF647 and the TNT index determined from up to 1440 cells per condition (*n* = 4, two-way ANOVA, Tukey’s post-hoc test). Representative image panels are shown in Supplementary Fig. 8c, d. **g** Airyscan superresolution live time-series microscopy (~30 min., Supplementary Video 4) of U2OS cells stably expressing GFP-WIPI1 was conducted, and merged GFP and brightfield channels are displayed (left image). Using the plug-in MTrackJ in Fiji, GFP-WIPI1 puncta were tracked within a TNT connecting two cells, and GFP-WIPI1 tracks of different puncta are indicated in distinct colours whereby the movement direction is indicated with colour-corresponding arrows (Supplementary Video 5). Scale bar: 20 μm. **h** U2OS GFP-WIPI1, **i** U2OS GFP-WIPI2 or **j** U2OS GFP-LC3 donor cells were cocultured (ratio 1:1) in fed conditions for 24 h with recipient U2OS cells either expressing wild-type ATG16L1 (WT) or not (U2OS ATG16L1 KO), along with ATG16L1 KO cells reconstituted for WT ATG16L1 (ATG16L1 KO + ATG16L1 WT). GFP puncta fluorescence from GFP-WIPI1, GFP-WIPI2 or GFP-LC3 within recipient MLS-EGFP-mCherry ATG16L1 U2OS cell lines bearing labelled mitochondria was clearly distinguishable from mitochondria, which appeared as orange fluorescence in recipient cells and which lacked GFP only puncta. Based on this, the numbers of recipient cells harbouring GFP-WIPI1 (**h**), GFP-WIPI2B (**i**) or GFP-LC3 (**j**) puncta were counted (one-way ANOVA with Holm-Sidak post-hoc test, up to 650 cells from *n* = 3 for each condition). **k** Representative merged images are displayed from (**h**) to (**j**), and green fluorescent puncta (arrows) in recipient cells (white asterisks), marked by mitochondrial staining (orange), are indicated (arrows). White boxes display zoomed-in sections. All cells, donor and recipient cells were stained with phalloidin-AF647 (violet) and DAPI (blue). All recipient cell lines were able to take up autophagic membranes decorated with GFP-WIPI1, GFP-WIPI2B or GFP-LC3, derived from respective donor cell lines in coculture settings. Extended image presentation in Supplementary Fig. 8e. **l**, **m** Donor U2OS cells stably expressing GFP-LC3 were transfected with different concentrations (0.2, 0.4 or 0.6 μg) of either control (9E10) or WIPI1 (9E10-WIPI1) expression plasmid for 24 h and then co-cultured with recipient U2OS cells stably expressing NLS-Scarlet for an additional 24 h. Subsequently, cells were stained with phalloidin-AF647 and DAPI. Representative images are shown (left panels). The percentage of recipient cells containing GFP-LC3 puncta derived from donor cells was calculated by manual counting (right panels, up to 700 cells per condition, two-way ANOVA with Tukey’s post-hoc test, mean ± SD, *n* = 3). Scale bar: 20 µm. Supplementary material is available (Supplementary Fig. 8; Supplementary Videos 2–5; Supplementary Data 1). *P* values: *p < 0.05; **p < 0.01; ***p < 0.001; ns not significant.

While assessing the intracellular WIPI1 localization, we observed that the formation of WIPI1 puncta was not uniform in cell monolayers and that cells with high puncta content sometimes lie adjacent to cells with little or no puncta content, even though all cells were starved. We hypothesized that there might be a compensatory mechanism that allows sufficient autophagy in all cells and assessed whether autophagic membranes positive for WIPI1, WIPI2, or LC3 could reach neighbouring cells, perhaps through tunnelling nanotubes (TNTs), intercellular bridges (Fig. 6a) through which cells exchange organelles, such as lysosomes^66–69^. In fact, we found that U2OS cells can form TNTs, stained here with phalloidin to detect F-actin and with wheat germ agglutinin (WGA) to visualize the plasma membrane (Fig. 6b). Alternatively, we stained cells with phalloidin and an anti-tubulin antibody and confirmed that TNTs in U2OS cells could also contain both F-actin and tubulin (Supplementary Fig. 8a) according to previous observations in different cell types^66^. In line with this, TNTs did not form in the presence of latrunculin A, an F-actin inhibitor, or nocodazole, a tubulin inhibitor (Supplementary Fig. 8b). Since it has been found that lysosomes can move through TNTs^70^, we asked if autophagic membranes could be observed in TNTs. For this aim we used a stable GFP-WIPI1 U2OS cell line as well as a stable tandem fluorescent RFP-GFP-LC3 U2OS cell line (Fig. 6c) and observed that, indeed, autophagic membranes are present in TNTs, and such GFP-WIPI1-harbouring TNTs are exemplified using a 3D video reconstruction from confocal laser-scanning microscopy (Supplementary Video 2), and a still image from this video displaying GFP-WIPI1 puncta in a TNT is shown (Fig. 6c, left panel). That autophagosomes are also found in TNTs is indicated by the identification of RFP-GFP-LC3 puncta in TNTs (Fig. 6c, right panel; Supplementary Video 3).

On this basis, we extended TNT assessment in U2OS cells stably expressing GFP-WIPI1 using fed and starved conditions (Fig. 6d). While the number of puncta-positive GFP-WIPI1 cells increased significantly during starvation-induced autophagy, as expected (Fig. 6d, left panel), the number of TNTs also increased (Fig. 6d, middle panel), as did the number of TNTs containing GFP-WIPI1 puncta (Fig. 6d, right panel). Furthermore, we found that the presence or absence of WIPI1 affects TNT formation (Fig. 6e, f). Transient overexpression of GFP-WIPI1 significantly increased the number of TNTs (Fig. 6e), while WIPI1 deficiency significantly decreased the number of TNTs and starvation-induced TNT formation was significantly attenuated in U2OS cells (Fig. 6f; Supplementary Fig. 8c, d). Importantly, using live-cell microscopy, we tracked GFP-WIPI1 puncta through the TNTs (Fig. 6g; Supplementary Video 4, 5). These results demonstrate that starvation-induced autophagy is accompanied by the formation of TNTs and the transport of autophagic membranes to neighbouring cells via the TNTs. To investigate further, asking if autophagic membranes can indeed invade neighbouring cells and whether such a scenario could represent a compensatory response to certain cells with low autophagic activity, we developed the following experimental design. We used cocultures of U2OS cells stably expressing GFP-WIPI1, GFP-WIPI2B or GFP-LC3^9^, which were defined as donor cells, along with U2OS containing labelled mitochondria^39^, which were defined as recipient cells. U2OS cells with labelled mitochondria expressed ATG16L1 (U2OS WT), were ATG16L1 deficient (U2OS ATG16L1 KO), or expressed ATG16L1 in an ATG16L1 deficient background (U2OS ATG16L1 KO + ATG16L1)^39^. Using this approach, we quantified the number of recipient cells with GFP puncta and showed that trafficking of autophagic membranes positive for GFP-WIPI1 (Fig. 6h), GFP-WIPI2B (Fig. 6i) and GFP-LC3 (Fig. 6j) was significantly increased towards autophagy-incompetent recipient cells (U2OS ATG16L1 KO). Representative images of recipient cells containing GFP-WIPI1, GFP-WIPI2B or GFP-LC3 puncta are shown (Fig. 6k; Supplementary Fig. 8e). Our results suggest that low levels of autophagy in single cells may be compensated for by cell-to-cell communication, with cells with a higher proportion of formed autophagic membranes or autophagosomes spreading a subfraction thereof to cells with low autophagic activity. In this context, it is conceivable that an enhancing role of WIPI1 in the formation of autophagic membranes should therefore also influence the extent of autophagic membrane transport by TNTs. This assumption turned out to be relevant since the transfer of GFP-LC3 positive autophagosomes was significantly increased when WIPI1 was overexpressed (Fig. 6l, m).

### ABL deficiency elevates autophagy and extends lifespan in *C. elegans*

**Fig. 7:**
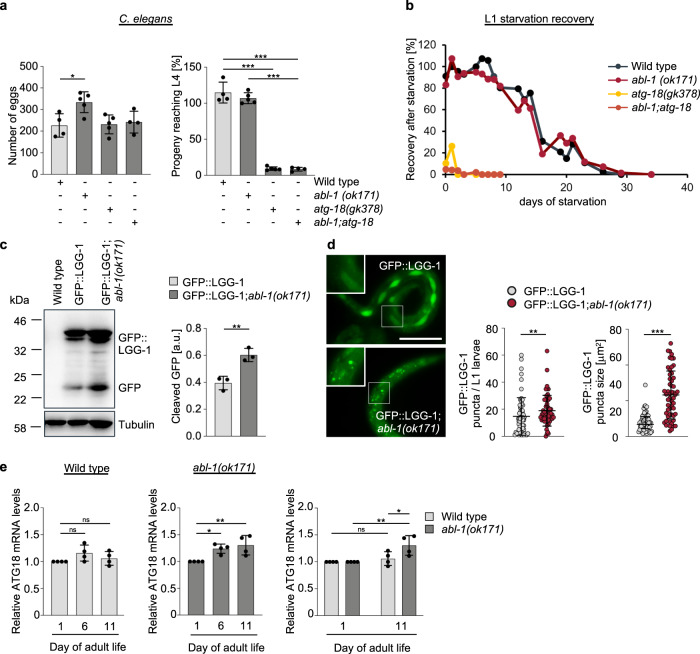
ABL1 deficiency in *C. elegans* increases ATG-18 gene expression and autophagic flux. **a** Hermaphrodites of the N2 wild type, *abl-1(ok171)* mutant, *atg-18 (gk378)* mutant or *abl-1(ok171);atg-18 (gk378)* double mutant strains were singly transferred to NGM plates, and eggs per hermaphrodite were counted over the whole reproductive period, *n* ≥ 4 (left panels). The number of L4 nematodes was calculated as a percentage of the total number of eggs laid (right panels). One-way ANOVA with Holm-Sidak post-hoc test, mean ± SD, *n* ≥ 4. **b** For the L1 starvation assay, eggs were isolated, and L1 larvae hatched in nutrient-free medium, in which they were kept for up to 34 days. Every 2-3 days, larvae were removed from starvation and spotted onto NGM/OP50 plates. After 2 days of unrestricted feeding, nematodes that reached the L2 larval stage or later were scored, and the percentage of developing larvae was calculated. The corresponding results for WT, *abl-1(ok171)* mutant, *atg-18 (gk378)* mutant or the *abl-1(ok171);atg-18 (gk378*) double mutant strain are shown. **c** N2 wild-type, and wild-type (GFP::LGG1) or *abl-1(ok171)* mutant (GFP::LGG1; *abl-1(ok171*) L1 larvae expressing the adIS2122 transgene GFP::LGG1 were starved for 16 h. Protein extracts from whole larvae were analysed by immunoblotting against GFP or tubulin (left panels). Relative protein levels of cleaved GFP over tubulin were quantified (right panel, Welch’s *t* test, mean ± SD, *n* = 3). **d** Likewise, wild-type (GFP::LGG1) or *abl-1(ok171)* mutant (GFP::LGG1; *abl-1(ok171)*) L1 larvae expressing the adIS2122 transgene GFP::LGG1 were imaged (left panels) and GFP-LGG1 puncta number (middle panels) as well as the mean puncta size (right panels) per nematode determined using CellProfiler. For statistical analysis, an unpaired *t*-test with Welch’s correction was performed (GFP::LGG1, 61 nematodes; GFP::LGG1; *abl-1(ok171*, 55 nematodes; mean ± SD). Scale bar = 50 µm. **e** Total RNA from synchronised and sterilised wild-type or *abl-1(ok171)* nematodes was extracted on day 1, day 6 and day 11 of adulthood. Relative ATG-18 mRNA levels were analysed (Welch’s *t* test, mean ± SD, *n* = 4). Left panel: ATG-18 expression in wild type nematodes. Middle panels: ATG-18 expression in *abl-1(ok171)* nematodes. Right panel: Additional comparative display of results (left panels, middle panels) of day 1 and day 11 only. Supplementary material is available (Supplementary Data 1). *P* values: *p < 0.05; **p < 0.01; ***p < 0.001; ns not significant.

In our final component of this study, we wanted to address the role of WIPI1 as an autophagy enhancer in a physiological context. We predicted that if ABL signalling negatively affects WIPI1 expression and autophagy in human cells, this level of autophagy control should also play an important role in lower eukaryotes such as *C. elegans* because the autophagy pathway is evolutionarily conserved. Furthermore, since a large number of previous studies reported that key signalling pathways that control autophagy are important in controlling lifespan through their effect on autophagy^2,71,72^, we speculated that ABL deficiency would not only increase autophagy but could therefore also prolong lifespan. In *C. elegans*, ABL1 and ABL2 have a single homologue, ABL-1, and for our studies, we used the *C. elegans* strain XR1 expressing a nonfunctional ABL-1 mutant (*abl-1(ok171*)^73^. In addition, we employed the VC893 strain (*atg-18(gk378)*), lacking ATG-18^74,75^, the orthologue for human WIPI1/WIPI2^7,76^, and generated an *abl-1(ok171);atg-18(gk378)* double mutant (*abl1;atg-18*). Initially, we assessed the viability of these strains and observed no significant differences in terms of egg-laying capacity (Fig. 7a, left panel), while the progeny that reached the fourth larval (L4) stage were significantly reduced to 10% in the *C. elegans* strain lacking ATG-18 (*atg-18(gk378)*), as previously reported^74,75,77^ (Fig. 7a, right panel). This was also observed for the *C. elegans* strain lacking both ABL-1 and ATG-18 function (*abl1;atg-18*) but not when ABL1 function alone was missing (*abl-1(ok171)*) (Fig. 7a, right panel). Next, we assessed the recovery of first-stage (L1) larvae after prolonged starvation followed by unrestricted feeding (Fig. 7b), indicating autophagy-mediated compensation for nutrient deficiencies in early life^75^. In a time course experiment, we starved L1 larvae for extended periods of time (days of starvation) and then unrestrictedly subjected larvae to food and counted the number of larvae able to survive and progress beyond the L2 larvae stage (Fig. 7b). As expected*, atg-18(gk378)* larvae were unable to recover from starvation of more than one day^75^. Only 5% of the *abl-1;atg-18* double mutant larvae were able to recover from up to 3 days of starvation, but not beyond 3 days (Fig. 7b). In contrast, *abl-1(ok171)* larvae were able to survive and recover from prolonged starvation of up to 29 days, as were wild-type larvae (Fig. 7b). Further, we demonstrate that in this context, autophagy is indeed more active in L1 larvae without ABL-1 function (*abl-1(ok171)*) by measuring the level of cleaved GFP in *abl-1(ok171)* nematodes carrying a GFP::LGG1 reporter transgene. Compared to wild-type nematodes, *abl-1(ok171)* nematodes showed significantly increased levels of cleaved GFP, indicating an increase in autophagic flux (Fig. 7c). In line with this result, both the number and size of GFP-LGG1 puncta increased significantly in *abl-1(ok171)* nematodes carrying a GFP::LGG1 reporter transgene (Fig. 7d).

Further, to determine whether we could observe a difference in ATG-18 gene expression in ABL-1-deficient nematodes, as we did for WIPI1 in human cells, we examined ATG-18 mRNA levels at three different time points in the lifespan of *C. elegans*, day 1, day 6 and day 11 of adulthood. Indeed, we observed a significant increase in ATG-18 mRNA levels from day 1 to day 6 as well as to day 11 in adult nematodes lacking ABL-1 function (Fig. 7e, middle panel), such increase was not observed in wild-type nematodes (Fig. 7e, left and right panels).

**Fig. 8:**
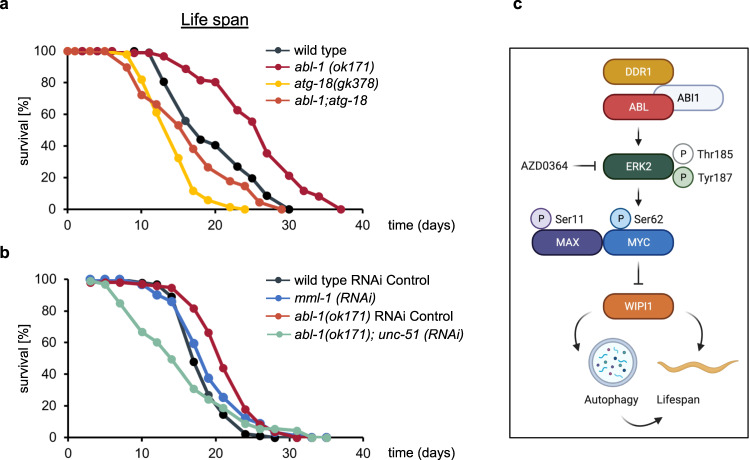
ABL1 deficiency in *C. elegans* increases lifespan in an ATG18-dependent manner. **a** For lifespan assessments, eggs were isolated by hypochlorite treatment and grown on NGM/OP50 plates until they reached the L4 larval stage. L4 nematodes were then transferred to NGM/OP50/FUdR plates to sterilise the nematodes, and surviving nematodes were counted every 2–3 days. The resulting lifespan curves are shown (statistical OASIS analysis, Supplementary Table 1). **b** Likewise, lifespan assessments were conducted while depleting the c-MYC homologue MML-1 in N2 wild type (*mml-1 (RNAi)*) or the ULK homologue UNC-51 in the *abl-1(ok171) strain (abl-1(ok171); unc-51 (RNAi))* by RNA interference. Lifespan curves are shown and statistical OASIS analysis is displayed in Supplementary Table 2). **c** A predicted model for the regulation of WIPI1 gene expression by the ABL/MYC axis and its impact on autophagy and lifespan in *C. elegans*. Created with BioRender.com.

We further performed adult lifespan assessment for all four *C. elegans* strains (Fig. 8a**;** Supplementary Table 1), which strikingly showed that ABL-1 deficiency in *abl-1(ok171)* nematodes significantly extended both the mean lifespan and the overall lifespan (Fig. 8a; Supplementary Table 1) compared to wild-type nematodes. ABL-1 deficiency-mediated lifespan extension was dependent on autophagy, since *abl-1;atg-18* double mutant nematodes showed survival rates similar to those of *atg-18(gk378)* nematodes, whose lifespans were expected to be shortened^77^ (Fig. 8a; Supplementary Table 1). We have also confirmed that lifespan extension due to ABL-1 deficiency is dependent on autophagy, since depleting UNC-51, the ULK homologue in *C. elegans*, in the ABL-1 deficient strain by RNA interference (*abl-1(ok171); unc-51 (RNAi)*) counteracted lifespan extension (Fig. 8b; Supplementary Table 2). Furthermore, we show by RNA interference in wild-type nematodes that the lack of MML-1, which shares some functional similarities with mammalian MYC^78–80^, also contributes to an extended lifespan, but to a much lesser extent than ABL-1-deficient *C. elegans* (Fig. 8b; Supplementary Table 2).

Taken together, these results strongly suggest that ABL-1 signalling inhibits autophagy, and when this negative impact is removed, *C. elegans* lifespan is prolonged through increased ATG-18 gene expression and autophagy enhancement.

## Discussion

According to the current paradigm, lifespan-extending signalling pathways culminate in the process of autophagy, which essentially defines the lifespan of eukaryotes^2,71,72^. In this context, model organisms’ orthologues of human WIPI1, such as ATG-18 in *C. elegans*, have been identified as one of the most critical autophagic factors for lifespan extension^72,77^. However, since ATG-18 is the orthologue for both human WIPI1 and WIPI2^7,76^ and the lack of WIPI2 but not WIPI1 severely impairs autophagosome formation^9^, the specific role of human WIPI1 in autophagy and lifespan control remains unclear. Nevertheless, it has been found that in human cells and in response to PI3P production at the onset of autophagy, WIPI1 assists WIPI2^9^ in efficiently recruiting the ATG16L1 complex to the nascent autophagosome for subsequent LC3/GABARAP lipidation and autophagosome maturation^9,27,28^. Interestingly, in the context of human lifespan, it has been reported that centenarians are characterized by an increased expression of WIPI1^81^, indicating that WIPI1 gene expression may be relevant to autophagy-related lifespan determination. Here, we provide a mechanistic explanation for why WIPI1 should indeed play this important role.

We have identified here the ABL-MYC signalling axis to repress WIPI1 gene expression and have shown that eliminating this negative impact increases WIPI1 mRNA levels, promotes autophagy and extends *C. elegans* lifespan (Fig. 8c). This finding is consistent with the notion that MYC counteracts lifespan extension in mice^82^ and provides insight into a novel pathway that controls lifespan through autophagy. In this context, WIPI1 can be viewed as an autophagy enhancer since elevated WIPI1 protein levels can promote abundant formation of autophagic membranes. Although this increase in autophagic membranes is not essential for the process of autophagy, because WIPI1 deficiency does not abolish autophagy^9^, it should become relevant under circumstances of reduced autophagic activity, for example, during the ageing process^83^. WIPI1 seems to be predestined for this function, since WIPI1 is generally weakly expressed and the protein interactome of WIPI1, in contrast to the other WIPI proteins, is limited and with regard to interacting ATG proteins, is restricted to self-interaction and heterodimerization with WIPI2. This is ideal for strengthening the WIPI2 function by having more WIPI1 without disturbing the balance of or sequestering other ATG functions. That elevated WIPI1 levels are associated with improved survival is also indicated by the finding that elevated WIPI1 levels have prognostic survival value in human melanoma patients^84^.

Surprisingly, while analysing the abundance of WIPI1-decorated autophagic membranes, we observed their presence in tunnelling nanotubes (TNTs), which are direct connections between cells that mediate intercellular communication^66,68^ and have a role in organelle transfer^67,69^. Since we considered WIPI1 as an enhancer of autophagic membrane formation, we hypothesized that this role might be extended to neighbouring cells and reasoned that this fact might be relevant to special circumstances such as extremely unequal levels of autophagy in neighbouring cells. Ultimately, using coculture conditions with human cells lacking ATG16L1 that were therefore autophagy incompetent^39^, and with cells expressing GFP-tagged WIPI1, WIPI2 or LC3, we provide evidence that autophagic membranes are indeed found in autophagy-deficient cells if they can form TNTs with autophagy-competent cells. Furthermore, our study showed that WIPI1 deficiency reduces both the formation of TNTs and the transport of LC3-positive autophagic membranes through TNTs. Although this finding now warrants the collection of molecular details in future studies, important for a mechanistic understanding of TNT formation and selectivity in autophagic cargo transport by TNTs, it underscores that autophagic membrane transport through TNTs should be a vital mechanism by which cells with high autophagic activity may rescue cells with low autophagic activity.

In the context of our functional assignment of WIPI1 as an autophagy enhancer relevant to lifespan extension, we propose that this autophagy enhancement may not be confined to single cells but that such a surplus can be spread through TNTs to neighbouring cells if needed. During the ageing process, this scenario may explain why a WIPI1-mediated increase in autophagy can have far-reaching beneficial effects. Hence, it can be speculated that an increase in WIPI1 gene expression with age, as seen in centenarians^81^, could have beneficial effects on human health span. In line with our results and deduced paradigm, it has recently been shown that mild elevation of autophagy through forced overexpression of Atg1 (ULK1 in humans) extended lifespan^85^.

However, it should be noted that our presented lifespan data in the context of the ABL-MYC-WIPI1 axis, was carried out in the model organism *C. elegans*. Hence the question arises as to whether the outcome can be transferred to the organismic complexity of the aging process in humans. However, our study integrates with the current focus on deciphering the regulation of human aging, with the goal of being able to extend human healthspan in the future. To what extent an increased WIPI1 expression could have a positive effect in this context can currently only be speculated. Nevertheless, the finding that centenarians have elevated WIPI1 levels warrants further study.

## Methods

### Primary antibodies

The following primary antibodies were used: c-Abl (Cell Signaling Technologies, 2862; WB: 1:1000), CrkL (Santa Cruz Biotechnologies, sc-319; WB: 1:1000), DDR1 (Cell Signaling Technologies, 5583; WB: 1:1000), ERK2 (Cell Signaling Technologies, 9108; WB: 1:1000), GAPDH (Santa Cruz Biotechnologies, sc-47724; WB: 1:1000), GABARAPL1 (Cell Signaling Technologies, 26632; WB: 1:1000), GFP (Roche, 11814460001; WB: 1:1000), LC3 (nanoTools, 0231-100/LC3-5F10; WB: 1:1000), c-myc (9E10) (Santa Cruz Biotechnologies, sc-40; WB: 1:1000), c-myc (Cell Signaling Technologies, 9402S; WB: 1:500), p62 (Santa Cruz Biotechnologies, sc-28359; WB: 1:1000, IF: 1:50), p62 (Medical and Biological Laboratories, PM045; WB: 1:500-1:1000), S6K (Cell Signaling Technologies, 2708, WB: 1:500-1:1000), TFEB (Cell Signaling Technologies, 37785, WB: 1:500), α-Tubulin (Sigma‒Aldrich, T5168; WB: 1:50000, IF 1:2000) and ULK1 (Cell Signaling Technologies, 8054; WB: 1:500–1:1000).

The following phospho-specific primary antibodies were used: phospho-CrkL (Tyr207) (Cell Signaling Technologies, 3181S; WB: 1:1000), phospho-p44/42 ERK1/ERK2 (Y204/Y187) (Cell Signaling Technologies, 5726; WB: 1:1000), phospho-MAX (S11) (Thermo Fisher, PA5-97346; WB: 1:500), phospho-c-Myc (Ser62) (Cell Signaling Technologies, 13748; WB: 1:500), phospho-S6K (Cell Signaling Technologies, 9234, WB: 1:500-1:1000), phospho-TFEB (Ser211) (Cell Signaling Technologies, 37681, WB: 1:500) and phospho-ULK1 (Ser757) (Cell Signaling Technologies, 6888, WB: 1:500-1:1000).

### Secondary antibodies

The following secondary antibodies were used: Alexa Fluor 488 goat anti-rabbit IgG (Life Technologies, A-11008, IF 1:200), anti-mouse IgG HRP-linked (Cell Signaling, 7076; WB 1:5000–1:10,000), and anti-rabbit IgG HRP-linked (Cell Signaling, 7074; WB 1:5000–10,000).

### Dyes

The following dyes were used: 4,6-diamidino-2-phenylindole (DAPI) (AppliChem, A1001), Alexa FluorTM 546 Phalloidin, hereafter Phalloidin-AF546 (Thermo Fisher, A22283, 1:1000), Alexa FluorTM 647 Phalloidin, hereafter Phalloidin-AF647 (Thermo Fisher, A22287, 1:1000), and Wheat Germ Agglutinin, Alexa FluorTM 647 Conjugate, hereafter WGA-AF647 (Thermo Fisher, W32466, 1:200).

### Plasmids

The following plasmids were used to express tagged WIPI1 and WIPI2 fusion proteins: GFP-WIPI1^7,9^, GFP-WIPI2B^7,9^, 9E10-WIPI1^14^ and mCherry-WIPI1^86^. pCMV6-MAPK1 (MR222304-OR) and pCMV6-Entry (PS100001) were purchased from Origene. pCMV-myc-ERK2-MEK1_fusion was provided by Melanie Cobb (Addgene, #39197). pCMV3-untagged negative control plasmid (CV011) and pCMV3-untagged c-MYC cDNA ORF (HG11346-UT) were purchased from SinoBiological through BIOZOL Germany. For conducting dual-luciferase assays, the plasmids pGL4.73[hRluc/SV40] (E6911) and pGL4.23[luc2/minP] (E8411) were purchased from Promega. Using pGL4.23[luc2/minP], the plasmids pGL4.23-5xE-box and pGL4.23-WIPI1promotor employed for luciferase assays in this study, were prepared by AZENTA Life Sciences, GENEWIZ Germany custom cloning service. The pGL4.23-5xE-box plasmid was generated by custom order synthesis of a 312 bp DNA fragment that contained 5 canonical E-boxes (GCCACGTGCA) spaced by 50 nucleotides (referred to as 5xE-box), while the 5’-site was designed to match the XhoI and 3’-site to match the HindIII restriction site of the pGL4.23[luc2/minP] multiple cloning region. Subsequently, the 5xE-box fragment was cloned into pGL4.23[luc2/minP] (XhoI/HindIII) to generate pGL4.23-5xE-box. The pGL4.23-WIPI1promotor plasmid was generated by custom order synthesis of the WIPI1 promotor sequence (1555 bp) spanning the region from minus 1226 bp upstream to plus 329 bp downstream of the transcription start site, harbouring all three MYC-binding sites identified in this study (M + 1, M11, M19). In addition, 5’-NheI and 3’-HindIII recognition sites were added for cloning into pGL4.23[luc2/minP] (NheI/HindIII) to generate pGL4.23-WIPI1promotor. Plasmid integrities were verified by DNA sequencing (AZENTA Life Sciences, GENEWIZ Germany). The plasmids pLentiGuide (Addgene, # 117986), pLenti_mx2-PGK-BSD (a gift from Koraljka Husnjak Frankfurt CRISPR/Cas Screening Center (FCSC), Goethe University Frankfurt am Main, Germany), pPAX2 (Addgene, #12260), pMD2.G (Addgene, #12259) were used for lentiviral particle production in the course of generating U2OS WIPI KO cells.

### Cell culture

Human U2OS osteosarcoma cells (ATCC, HTB-96) or human G361 malignant melanoma cells (ATCC; CRL-1424) were cultured in Dulbecco’s modified Eagle medium (DMEM) GlutaMAX (Life Technologies, 31966)/10% fetal bovine serum (Life Technologies, 10270-106) supplemented with 100 U/ml penicillin/100 mg/ml streptomycin (Life Technologies, 15140-122) at 37 °C and 5% CO2. Monoclonal U2OS cells stably expressing GFP-WIPI1, GFP-WIPI2B, GFP-LC3^9^, or RFP-GFP-LC3 were obtained by G418 selection and cultured in the presence of 0.6 mg/ml G418 sulfate (Life Technologies, 11811-031). Monoclonal U2OS cell lines deficient in ATG16L1 (ATG16L1 KO) or reconstituted for ATG16L1 expression (here referred to as ATG16L1 KO + ATG16L1 WT) stably expressing MLS-EGFP-mCherry were cultured in DMEM GlutaMAX (Life Technologies, 31966) supplemented with 10% fetal bovine serum (Life Technologies, 10270-106) and 100 U/ml penicillin/100 mg/ml streptomycin (Life Technologies, 15140-122) at 37 °C and 5% CO_2_. Monoclonal U2OS cell line stably expressing NLS-mScarlet was likewise cultured in DMEM GlutaMAX (Life Technologies, 31966) supplemented with 10% fetal bovine serum (Life Technologies, 10270-106) and 100 U/ml penicillin/100 mg/ml streptomycin (Life Technologies, 15140-122) at 37 °C and 5% CO_2_.

### Generation of WIPI1 knockout U2OS cells

For generating U2OS cells deficient in WIPI1 (here referred to as WIPI1 KO), CRISPR-Cas gRNA sequences targeting human WIPI1 were designed using a gradient-boosted regression trees model with an augmented feature set (Rule Set 2)^87^. The top three scoring gRNAs

WIPI1-1 GAGCAGCTGGATCAAGTCCA,

WIPI1-2 TAGTCAGTCACACAAAACCA,

WIPI1-3 GACCAGAAGAGCCTTCGACC

were selected and 3Cs-based cloned as mini-pool into pLenti-Guide^88,89^. Generation of lentiviral particles were performed by using HEK293T cells and lentiviral supernatant was harvested 48 hours after transfection and stored at −80 °C. Subsequently, hTERT-U2OS Cas9 cells (a gift from Andrew Holland, John Hopkins School of Medicine, Baltimore, MD, USA) were used to generate WIPI1 KO cells that were cultured in DMEM/F-12 (Life Technologies, 11320-074) supplemented with 10% fetal bovine serum (Life Technologies, 10270-106), 100 U/ml penicillin/100 mg/ml streptomycin (Life Technologies, 15140-122) and 0.1% Blasticidin (Gibco, A11139-03) at 37 °C and 5% CO_2_.

### Autophagy assays

Cells were washed three times with Dulbecco’s phosphate buffered saline (DPBS, Life Technologies, 14190144) before incubation in full (fed, DMEM/10% FCS), serum starvation (DMEM) or starvation (starved, Earle’s balanced salt solution (EBSS), Life Technologies, 24010-043) medium in the presence or absence of 200 nM (AppliChem; A7823) or 100 nM (EMD Millipore, 196000) bafilomycin A1. Autophagy assays were generally performed for three hours unless stated otherwise. Moreover, AZD0364 (final concentration: 10 µM, Selleckchem, S8708), MG132 (final concentration: 10 µM, Selleck Chemicals, S2619), DPH (final concentration: 10 µM, Sigma‒Aldrich, SML0202), cycloheximide (final concentration: 10 µg/ml Applichem, A0879), LY294002 (SelleckChem, S1105; final concentration: 100 µM), 10058-F4 (SelleckChem S7153; final concentration: 1 µM), 10074-G5 (SelleckChem S8426; final concentration: 1 µM), and dasatinib (SelleckChem, S5254; final concentration: 1 µM) were added for the indicated times.

### DNA transfection

Transient DNA transfections were conducted using Lipofectamine 2000 (Invitrogen, 11668019). When using 24-well plates, 0.1-0.8 µg DNA and 0.2–2.0 µl Lipofectamine 2000 were diluted in 25-30 µl OPTI.MEM (Life Technologies, 51985026) each and incubated for 5–20 min at room temperature. Both mixtures were combined and incubated for another 20–30 min at room temperature before the addition of 50 µl of the mix into one well of a 24-well plate containing 50,000 cells in 200–500 µl DMEM/10% fetal bovine serum (FBS) or OPTI.MEM and transfection was carried out up to 48 h. When using 96-well plates, 100 ng of DNA and 0.25 μl Lipofectamine 2000 were diluted in 5 μl OPTI.MEM each and incubated for 5 min at room temperature prior combining the mixtures and subsequent incubation (20 min at room temperature). This mix (10 μl) was added to one well of a 96-well plate containing 10,000 cells in 100 μl DMEM/10% fetal calf serum (FCS).

### RNA interference

Knockdown experiments using siRNAs were performed using Lipofectamine RNAiMAX (Invitrogen, 13778150) as follows. siRNAs (Santa Cruz: 25 nM or Origene: 3 × 20 nM) and 1 µl Lipofectamine RNAiMAX were diluted in 100 µl OPTI.MEM (Life Technologies, 51985-026) and incubated at room temperature for 20 min. The mixture was then added to one well of a 24-well plate, and 500 µl of cell suspension containing 50,000 U2OS cells in DMEM/10% FCS was seeded on top of the transfection solution for 48 h. Knockdown was confirmed by qPCR and Western blotting. The following siRNAs were purchased from Santa Cruz Biotechnology: c-Abl (Abl1) siRNA (h) (sc-29843), Arg siRNA (Abl2) siRNA (h) (sc-38945), DDR1 siRNA (h) (sc-35187), and control siRNA-A (sc-37007). The following siRNAs were purchased from OriGene Technologies: c-Abl (ABL1) Human siRNA Oligo Duplex (SR3000017/SR319232), ABL2 Human siRNA Oligo Duplex (SR3000019/SR319233), MCK10 (DDR1) Human siRNA Oligo Duplex (SR300547/SR319533), c-Myc (MYC) Human siRNA Oligo Duplex (SR321047), MAX Human siRNA Oligo Duplex (SR302818), and Trilencer-27 Universal scrambled negative control siRNA duplex (SR30003/SR30004).

### RNA extraction and quantitative PCR (qPCR)

Total RNA extraction was performed using the RNeasy Mini Kit (QIAGEN, 74104) in combination with QIAshredder (QIAGEN, 79654) and the RNase-free DNase Set (QIAGEN 79254). After washing, the cells were resuspended in RLT buffer. The cell suspension was then added onto the QIAshredder column and lysed at 14,200 rpm for 2 min. The flowthrough was mixed with an equal volume of 70% ethanol, immediately transferred to a RNeasy extraction column, and centrifuged at 10,000 rpm for 15 s. After one wash with RW1 buffer, DNA was digested on-column for 15 min using DNase I, after which the column was washed again once with RW1 and twice with RPE buffer. RNA was then eluted in RNase free water. Concentrations were determined using a Nanodrop 1000, and the RNA was transcribed using TaqMan Reverse Transcription Reagents (Thermo Fisher, N8080234). qPCR was performed using 2x Taqman Fast Advanced Master Mix (Applied Biosystems, 4444963) and the following 20x Taqman Gene Expression Assays (Applied Biosystems, 4331182) with the following assay identification (ID) numbers: Hs01104728_m1 (Abl1), Hs00943652_m1 (Abl2), Hs01058430_m1 (DDR1), Hs04194186_s1 (FOS), Hs00420895_gH (RPLPO), Hs00215872_m1 (WIPI1), Hs00255379_m1 (WIPI2), Hs00750495_s1 (WIPI3/WDR45B), and Hs01079049_g1 (WIPI4/WDR45). In 384-well plates, 6.25 ng of cDNA was used per 10 µl reaction containing 1x Taqman Fast Advanced Master Mix and the appropriate 1x Taqman Gene Expression Assay and qPCR was run using a QuantStudio7Flex Real-Time PCR System.

### Autophagy pathway-focused gene expression analysis using qPCR arrays

RNA was extracted for qPCR analysis of single genes (see above). The RNA concentration was determined using a Nanodrop 1000 and Qubit. cDNA was synthesized using the RT2 First Strand Kit (QIAGEN, 330404). Genomic DNA was eliminated by adding 4 µl GE buffer to 800 ng RNA in 16 µl RNase-free water and incubating the mixture at 42°C for 5 min and subsequently cooling it on ice. The reverse transcription mix was prepared by combining 8 µl 5x Buffer BC3, 2 µl Control P2, 4 µl RE3 Reverse Transcriptase Mix, 6 µl RNase-free water and 20 µl of the genomic DNA elimination mix. Synthesis of cDNA was conducted with the following PCR cycler program: 15 min 42 °C and 5 min 95 °C. After that, 40 µl of cold RNase-free water were added to each reaction and the samples were placed on ice. Downregulation of the kinases was confirmed by TaqMan qPCR (see above). The cDNA was then used in the RT2 Profiler PCR Array (QIAGEN, PAHS-084Z) in combination with RT2 SYBR Green qPCR Mastermix (QIAGEN, 330529) using a QuantStudio7Flex Real-Time PCR System. Per 10 µl reaction, 5 µl RT2 SYBR Green qPCR Mastermix, 0.78 µl cDNA synthesis reaction and 4.22 µl RNase-free water were used. qPCR reaction was conducted with the following program: 1 cycle with 10 min 95 °C, 40 cycles with 15 s 95 °C and 1 cycle with 1 min 60 °C. Data analysis was performed in the data analysis webportal at Qiagen.com/geneglobe using the following parameters: CT Cut-off: 35, Normalisation method: Automatic Selection from Full Panel; Settings for visualization: fold regulation: 2, *P* value: 0.05.

### Fluorescence microscopy

Cells were grown on sterile coverslips and fixed using 3.7% formaldehyde. If appropriate, cells were stained with primary antibodies and secondary antibodies. Cell nuclei were stained with 4,6-diamidino-2-phenylindole (DAPI) (AppliChem, A1001) for 20 min at room temperature. Cells were mounted on glass slides using ProLong Gold Antifade Mountant (Life Technologies, P36930) and imaged using a laser scanning microscope (LSM) 800 (Zeiss) and a C Plan-Apochromat 63x/1.4 oil differential interference contrast (DIC) objective or a Leica SP8/HCX PL APO 100x/1.44 oil objective. Z-stacks were acquired with a slice distance of 0.5 μm. For 3D reconstruction, Z-stack images were obtained with a 40x/1.3 DIC Plan-Apochromat Oil-Immersion Objective (Zeiss).

### Automated quantitative confocal microscopy analysis

Image acquisition and analysis was performed in 96-well plates with glass bottom (Cellvis, P96-0-N) using a laser scanning microscope (LSM) 800 (Zeiss) and a C Plan-Apochromat 63x/1.4 oil differential interference contrast (DIC) objective. The automated image acquisition was carried out with the ‘tiles’ module of the ZEISS ZEN system 3.0 blue edition software (Carl Zeiss Microscopy Deutschland GmbH). Automated image analysis for the quantitative assessments of fluorescent puncta was then performed using CellProfiler (Version 4.2.1) with a pipeline designed for cell and puncta recognition^90^. In this CellProfiler pipeline, cell nuclei were identified as primary objects on DAPI images using the Otsu thresholding method to calculate a global thresholding value for each image. Cells were identified as secondary objects with the watershed method, using the nuclei as seed objects. Prior to puncta identification, the background of the images was subtracted using a median filter. Subsequently, the puncta were identified using the Otsu method to calculate a global threshold for each image. Then the puncta were assigned to cells based on spatial overlap and single cell values exported as a CSV spreadsheet for statistical analysis.

### Automated high throughput fluorescence-based imaging

Image acquisition and analysis were performed in 96-well plates. Cells were fixed using 3,7% formaldehyde, and cell nuclei were stained with 4,6-diamidino-2-phenylindole (DAPI) (AppliChem, A1001). Cells were kept in PBS, and images were acquired using the IN Cell Analyzer 1000 (GE Healthcare) using the Nikon Plan Fluor ELWD 40 × 0,6 objective. In each well of a 96-well plate, 20 images were taken, each with an average of 10 cells per image. Subsequent automated image analysis was performed using IN Cell Analyzer Workstation 3.4 software^9,30^.

### Lentiviral shRNA screen

The MISSION® LentiExpress™ Human Kinases (Sigma‒Aldrich, SHX001) shRNA library was screened as follows. 96-well plates precontaining shRNAs were thawed at room temperature for 10 min before centrifugation for 1 min at 1000 rpm. U2OS-GFP-WIPI1 cells were resuspended in DMEM/10% FCS supplemented with 11.4 µg/ml hexadimethrinbromide (Sigma Aldrich, H9268). A total of 2000 cells in 70 µl DMEM/10% FCS were seeded into each well of 96-well plates and incubated at 37 °C/5% CO_2_. After 24 h, the medium was replaced with 100 µl DMEM/10% FCS supplemented with 0.6 mg/ml G418 and 1 µg/ml puromycin for selection. The medium change was repeated after an additional 48 h. Twenty-four hours after the last medium change, the cells were washed twice with EBSS and starved for 3 h. The cells were then fixed with warm 3.7% paraformaldehyde (PFA) and stained with DAPI for 20 min. Automated image acquisition and analysis were performed using the IN Cell Analyzer 1000 and the In Cell Analyzer 1000 Workstation 3.4 software (GE Healthcare). Candidate kinases were chosen if they had two or more individual shRNAs showing >0,4 difference in fold increase or decrease compared to the mean of the control shRNAs. To identify overrepresented pathways, we performed statistical enrichment analysis of selected genes using the R (version 3.6.0) package gprofiler2 (version 0.1.8) with a hypergeometric test and the default gSCS method for multiple testing correction^91^. Pathways with *p* value less than 0.05 were treated as significantly overrepresented. Gene network plots of selected enriched pathways were generated using the clusterProfiler (version 3.12.0) R package^92^.

### Immunoblotting

Proteins were extracted by washing cells once with PBS and subsequently lysing in boiling 2x Laemmli buffer. The chromatin was sheared using a 23 G needle, and samples were boiled for 5 min. Alternatively, cells were washed and scraped into ice-cold ACA lysis buffer (750 mM aminocaproic acid, 50 mM Bis-Tris, 0.5 mM ethylenediaminetetraacetic acid (EDTA), pH 7.0, 0.1% Tween 20) supplemented with cOmplete EDTA-free protease inhibitor cocktail (Roche, 04693132001) and PhosStop phosphatase inhibitor cocktail (Roche, 04906837001). Cells were lysed by vortexing three times with a 5 min incubation on ice in between. After this, the protein extracts were spun down at 20,000 × *g* for 20 min, the supernatant was transferred to a fresh tube, and 4x Laemmli buffer was added. Before gel separation, the samples were boiled for 5 min. Proteins were separated by sodium dodecyl sulfate–polyacrylamide gel electrophoresis (SDS‒PAGE) and transferred to a 0,45 µm polyvinylidene difluoride (PVDF) transfer membrane (Thermo Scientific, 88518). Nonspecific binding was blocked by 5% BSA/TBS/T or 5% milk in TBS/T for one hour before antibody incubation overnight at 4 °C. The secondary antibody was incubated for one hour at 4 °C, and ECL analysis was performed using Signal West Femto Maximum Sensitivity Substrate (Thermo Scientific, 34095). Signal detection and analysis was conducted using the Amersham Imager 600 (GE healthcare) or the Fusion Sl instrument (Vilber Lourmat) and the Fusion Capt advance software (Vilber Lourmat). Alternatively, the iBright CL750 instrument (Thermo Scientific, A44116) was used along with the iBright Analysis Software (Thermo Scientific, Version 4.01).

### Phospho-SILAC analysis

In order to conduct phospho-SILAC based proteomics^93–99^, U2OS cells were labelled using light (SILAC-DMEM (PAN-Biotech, P04-02505S1) containing lysine-0/arginine-0/1% PenStrep (PAN-Biotech, P06-07100)/10% dialyzed FBS (PAN-Biotech, P30-2102)) or heavy (SILAC-DMEM containing lysine-8/arginine-10/1% PenStrep/10% dialyzed FBS) culture medium. ABL1/2 or DDR1 were transiently downregulated as described above, replacing OPTI.MEM with the appropriate heavy or light DMEM. siABL1/2 or siDDR1 KD was performed in heavy DMEM, while siControl KD was done in light DMEM. After 48 h, cells were lysed in lysis buffer (6 M urea, 2 M thiourea, 10 mM Tris pH 8.0, 1% N-octylglucoside (NOG)) for 10 min on ice. DNA and RNA were digested using Benzonase (Merck, 101695) for 10 min at room temperature followed by centrifugation at 2800 × *g* for 20 min. Proteins were precipitated by acetone/methanol (acetone:MeOH:sample 8:1:1) overnight at −20 °C, centrifuged (2000 × *g*, 20 min, 4 °C), and the protein pellet washed with 80% acetone and resuspended in lysis buffer without NOG. Bradford assay was performed to determine the protein concentration, and corresponding heavy and light samples were mixed 1:1. Proteins were digested with trypsin (Promega, V5113) overnight^93^. Twenty μg of peptides were directly desalted with C18 StageTips^94^. The rest of the peptide mixture was purified on Sep-Pak 18 cartridges (Waters), and phosphopeptides were enriched using TiO_2_ beads (Titansphere, 10 μm, GL Sciences) that were equilibrated in 80% acetonitrile (ACN), 1% trifluoroacetic acid (TFA), 3% 2,5-dihydroxybenzoic acid (DHB).Purified peptides were added in a bead to protein ratio of 1:2, and washed first with 30% ACN, 1% TFA, followed by 50% ACN, 1% TFA, and 80% ACN, 1% TFA. Elution of peptides was facilitated in two steps, first with 5% NH_4_OH in 20% TFA, and then with 80% ACN in 1% FA^99^. In total 10 enrichment cycles were run. All peptides were analysed on an Easy-nLC 1200 system coupled to a Q Exactive HF mass spectrometer (both Thermo Fisher Scientific)^95^ that was operated in positive ion mode in the m/z range of 300 to 1,650 with the following settings: peptides were separated with a 227 (proteome) or 57 min (phosphoproteome) segmented gradient from 10-33-50-90% of HPLC solvent B (80% ACN in 0.1% formic acid (FA)) in HPLC solvent A (0.1% FA) at a flow rate of 200 nl/min. The resolution of the MS full scan was 60,000 with target values of 3 × 10^6^ charges and a fill time of 25 ms. The twelve (proteome) or seven (phosphoproteome) most intense precursor ions were sequentially fragmented in each scan cycle using higher energy collisional dissociation (HCD) fragmentation, and sequenced precursor masses were excluded from further selection for 30 s. The target values for MS/MS fragmentation were 10^5^ charges with fill times of 45 (proteome) and 220 ms (phosphoproteome) and a resolution of 30,000 and 60,000, respectively. The data was processed with MaxQuant software (version 1.5.2.8) with integrated Andromeda search engine. The data was processed with MaxQuant software (version 1.5.2.8) with integrated Andromeda search engine. Database search was performed against a *Homo sapiens* database obtained from Uniprot, (93,827 entries, downloaded 20th of December 2017), and 286 commonly observed contaminants. Trypsin was defined as digestion enzyme with full specificity and a maximum of two missed cleavages. Carbamidomethylation on cysteine was defined as fixed modification, whereas phosphorylation of serine, threonine, and tyrosine, as well as oxidation of methionine and protein N-terminal acetylation were set as variable modifications. The precursor mass tolerance was set to 4.5 parts per million (ppm), whereas on fragment ion level 20 ppm was tolerated. The false discovery rate (FDR) was calculated by the target/decoy approach^97^ and set to 0.01 on peptide, protein and modification site level. Protein group quantitation was based on a minimum of two quantified peptides. Further downstream analyses were done with R (v 3.5.1). The requirement for a localized phosphorylation site was a reported localization probability of at least 0.75. The detected phosphorylation sites were normalised for changes in protein abundance. Taking into account the intensity distribution as well as ratios of all quantified peptides, a *P* value was calculated for each of the heavy to light ratio specifying significantly regulated phosphosites. A *P* value < 0.01 was defined as significantly regulated^98^.

### In silico prediction of MAX/MYC binding sites in the WIPI1 promoter

The online tool ConTra v3 was used to look for putative MAX/MYC binding sites in the WIPI1 promoter using the following parameters: Type of analysis: visualization; reference organism: Human (*Homo sapiens*); Transcript: WIPI1 chr17:66453653, number of introns:12, NM_017983; Sequence parts: promoter 5000 bp; Transcription factor: c-Myc:Max TRANSFAC20113,V$MYCMAX_B,M00322; stringency: core = 0.95, similarity matrix 0.85.

### Chromatin immunoprecipitation (ChIP)

Chromatin immunoprecipitation was carried out using the SimpleChIP® Enzymatic Chromatin IP Kit (Cell Signaling Technologies, 9002). U2OS cells were crosslinked using 1% formaldehyde for 10 min, washed once with ice-cold PBS/protease inhibitor cocktail (PIC) and pelleted at 2000 × *g*. After resuspension in Buffer A/DTT/PIC and 10 min incubation on ice with frequent inversion, nuclei were pelleted at 2000 × *g* for 5 min, washed once with Buffer B/DTT and resuspended in Buffer B/DTT for 20 min micrococcal nuclease treatment at RT. Nuclei were pelleted again and resuspended in ChIP buffer/PIC. After 10 min incubation on ice, the nuclear membrane was fragmented by sonication (BioRuptor Plus, 12 cycles, 20 s pulse/30 s rest at 4 °C). Samples were clarified by centrifugation, and the DNA quality was determined using a Nanodrop 1000 and agarose gel. Per immunoprecipitation 8 µg chromatin were used with the following antibodies: c-myc (Cell Signaling Technologies, 9402 S; ChIP: 2,4 µg), Di-Methyl-Histone H3 (Lys9) (H3KMe2) (Cell Signaling, 9753S; ChIP: 7,7 µg), G9a (Cell Signaling, 3306; ChIP: 0,8 µg), Max (Abcam, ab53570; ChIP: 3 µg), and Normal Rabbit IgG (Cell Signaling, 2729; ChIP: 6 µg). Immunoprecipitation was performed overnight at 4°C and Protein G agarose beads were used to capture antibodies. After three washing steps with ChIP buffer and one washing step with high salt buffer, elution was done in ChIP elution buffer for 30 min at 65 °C and 1200 rpm shaking. Proteinase K treatment was performed overnight and the DNA was cleaned up using DNA purification columns. qPCR analysis was performed using 2 µl of the DNA, SYBR Green PCR Master Mix (Applied Biosystems, 4309155) and 0.5 µM of the following primers:

M + 1_1F 5′-TTTCAACCAGGACTGCACGTAAGC-3′,

M + 1_1R 5′-ACAAGATCCCAATGCGTCCGAA-3′,

M11F 5′-TTGGAGTCTAACGCTCTCTGCTA-3′,

M11R 5′-AGGGTGACAGGAGGACGCGCTA-3′,

M19F 5′-ACAATCCAAAGCTGGCAGAGCTC-3′,

M19R 5′-CTCACGTAATTCGAGATAAACCTT-3′.

### Dual-Luciferase Reporter Assay

For luminescence measurement, U2OS cells were seeded into a 96-well plate (Greiner, 655083) co-transfected with pGL4.73[hRluc/SV40] and pGL4.23[luc2/minP], pGL4.23-WIPI1promotor or pGL4.23-5xE-box using Lipofectamine 2000 and incubated for 24 h. For cell lysis and luminescence detection, the Dual-Luciferase Reporter Assay System (Promega, E1969) was used. Cells were lysed by adding 20 µl of the 1× passive lysis buffer per well and shaking the culture plate for 15 min at room temperature. Addition of the firefly and Renilla luciferase substrates as well as luminescence measurement was performed using the Synergy Neo2 Multi-mode Microplate reader with the software BioTek Gen5 (Version 3.11) for automated liquid handling and luminescence intensity measurement. Firefly and Renilla luciferase activities were measured, and firefly activities normalized to Renilla luciferase activities.

### Correlative light electron microscopy (CLEM)

U2OS-GFP-WIPI1 cells were seeded to 50% confluency and starved for 6 h (see autophagy assay protocol above). Cells were fixed in 4% PFA/0.2 M 4-(2-hydroxyethyl)-1-piperazineethanesulfonic acid (HEPES) buffer pH 7.4 for 10 min at room temperature. PFA was replaced with 0.2 M HEPES buffer, and wide field images of GFP fluorescence and bright field images were acquired at 20x, 40x and 63x. Cells were further fixed using 2% glutaraldehyde for 2 h at room temperature and kept in 0.2 M HEPES buffer until processing for EM. For electron microscopy, the cells were washed twice with 0.1 M sodium cacodylate (NaCac) buffer, pH 7.4, and osmicated using 1% OsO4, 0.1 M NaCac buffer and 15 mg/ml K_4_[Fe(CN)_6_] for 1 h at room temperature. After two washes with 0.1 M cacodylate buffer and three washes with water, samples were incubated with 1% uranyl acetate for 1 h at 4 °C and washed three times with water. The samples were then dehydrated stepwise in 50%, 70%, 96%, and 100% ethanol for 3 min each. Finally, the samples were embedded in Epon resin (TAAB 812, TAAB Laboratories Equipment, 030) for 2 h at room temperature and baked at 60 °C for >14 h. Samples were then thin sectioned into 100 nm sections, which were stained with 0.5% uranyl acetate for 30 min and 3% lead citrate for 1 min before acquiring electron microscopy images on a Jeol JEM-1400 (Jeol) microscope at 80 kV.

### Live-cell microscopy

Live-cell imaging was performed using a Zeiss Cell Observer, consisting of an Axiovert 200 inverted microscope, EXFO X-Cite 120 illuminaCon system, Apotome module, and Pecon Incubator XL-3 equipped with a CO_2_/temperature control unit and heating unit. The incubation chamber of the microscope was brought to 37 °C, 5% CO_2_ prior to the start of the imaging. For live-cell microscopy, cells were cultivated to a confluency of 80–90% in custom-made 6-well plates with glass bottom. A 63x/1.4 DIC Plan Apochromat, EC Plan-Neofluor 100x/1.3 or an EC Plan Neofluor 40x/1.3 oil immersion objective were used to acquire a series of images in 5–15 s intervals. With the ApoTome module, a transmission grid (Grid H) was inserted into the beam path, and 3 images with different grid positions were acquired. The final images were calculated from the three raw images. The videos were generated with ImageJ by displaying 5 images per second^100^.

### Live-time tracking

Live time series microscopy was performed using a laser scanning microscope (LSM 800; Carl Zeiss GmbH) with a 40×/1,3 DIC Plan-Apochromat Oil-Immersion Objective (Zeiss). Cells were imaged in live cell imaging solution (Invitrogen, A14291DJ) supplemented with 5 mM or 20 mM glucose after starvation or full medium treatment for 24 h. During time series imaging, the cells were kept at 37 °C and 5% CO_2_. Airyscan superresolution images were obtained in combination with an electronically switchable illumination and detection module (ESID), with time intervals ranging between 30 and 60 s per position. Projects were processed with ZEN software (ZEISS), whereas the time series stacks were extracted and processed into videos in Fiji. Manual tracking of GFP-WIPI1 puncta within TNTs was performed with the free Fiji Plug-in MTrackJ, where GFP-WIPI1 puncta’s positions were manually selected through each time-lapse frame. Overlay videos were exported displaying tracks of each selected object.

### Giant unilamellar vesicles (GUVs)

For binding studies of GFP-WIPI with GUVs containing either PI3P or PI(3,5)P_2_, freshly prepared native cell extracts^9,14^ from U2OS cells stably expressing GFP-WIPI1 were used, along with control native extracts made from U2OS cells stably expressing GFP or from the parental U2OS cell line. GUVs were generated using a modified polyvinyl alcohol (PVA)-assisted swelling method. Coverslips were coated with a PVA solution (Sigma, 363065; 1% in double-distilled water (ddH_2_O)) and dried at 60 °C for 20 min. One hundred microlitres of each lipid mix was prepared in chloroform in a 4 ml glass bottle. All lipids were purchased from Avanti Polar Lipids. Lipid mixes containing 1,2-dioleoyl-sn-glycero-3-phosphocholine (DOPC, 850375 C), 18:1 PI(3)P (850150) or 18:1 PI(3,5)P_2_ (850154) and rhodamine-PE (810150) were prepared in chloroform at a molar ratio of 97:2:1. DOPC and rhodamine-PE in a 99:1 ratio were also employed as controls. Two microlitres of each lipid mix was spread onto the PVA-coated slide and then dried for 30 min at RT. Slide chambers with silicone isolators (Invitrogen, P18174) were assembled, and the lipid films were hydrated for 20 min at RT by adding 100 µl of 0.75 M sucrose solution. Protein extracts were diluted 1:5 in 0.8 M glucose solution and incubated for 50 min with GUVs (ratio 1:1) in slide chambers before imaging. Confocal LSM images were acquired with a Zeiss LSM 800 using a 40× air objective, and microscopy images were quantified using CellProfiler (Version 4.2.4), while GUVs were detected and filtered based on their rhodamine fluorescence, followed by correlation analysis and fluorescence-based size measurements.

### Tunnelling nanotubes (TNTs)

Using fluorescence microscopy (Zeiss, Axiovert 200 M) and a 100x oil objective, structures were counted as TNTs if they (i) connected two or more cells, (ii) contained F-actin and iii) did not touch the substrate. Phalloidin-AF546 or phalloidin-AF647 was used to stain F-actin filaments, whereas WGA-AF488 was applied to stain the plasma membrane. For the assessment of autophagic membranes moving between two cells through TNT connections, cocultures of donor and recipient cells were performed at a ratio of 1:1. For TNT inhibition, nocodazole (Sigma, M1404) or latrunculin A (Merck, 428021) was applied to inhibit microtubules and G-actin polymerization, respectively. The TNT index was defined as the number of TNTs per 100 cells.

### *C. elegans* strain maintenance

*Caenorhabditis elegans* strains were grown on NGM (nematode growth medium, 3 g/L NaCl, 17 g/L agar, 2.5 g/L peptone, 5 mg/L cholesterol, 1 mM MgSO_4_, 1 mM CaCl_2_, 2.5% v/v KPO_4_-buffer pH 6) (KPO_4_ buffer: 0.87 M KH_2_PO_4_, 0.13 M K_2_HPO_4_) plates seeded with *E. coli* OP50 at 15(°C using standard techniques. During the experiments, the nematodes were kept at 20 °C. The following strains were used in this study and provided by the CGC: N2 (wild-type/WT), VC893: *atg-18(gk378)*, XR1: *abl-1(ok171)*, DA2123: *lgg-1::GFP+rol-6(su1006)* and *E. coli* OP50. The *abl-1(ok171);atg-18(gk378)* and *abl-1(ok171);gfp::lgg-1* strains were generated in this study. OP50 bacterial cultures were prepared by inoculating the bacteria from the glycerol stock in liquid Luria-Bertani broth (LB) medium (10 g/L tryptone, 5 g/L yeast extract, 5 g/L NaCl) overnight at 37 °C and 180 rpm before seeding onto the NGM plates the next day.

### *C. elegans* egg isolation

Gravid adult nematodes were washed off nematode growth media (NGM) plates using water, spun down at 2000 rpm for 2 min and resuspended in bleach solution (2–4% NaOCl, 1.5 M NaOH). The tube was shaken until all worms were lysed and then filled with M9 buffer (3 g/L KH_2_PO_4_, 6 g/L Na_2_HPO_4_, 5 g/L NaCl, 1 mM MgSO4). The eggs were pelleted at 2000 rpm for 2 min and washed three times with M9 buffer.

### *C. elegans* brood-size assay

L4 hermaphrodites were singly transferred to NGM/OP50 plates and kept at 20 °C. The hermaphrodites were transferred to a fresh plate every day, and eggs on the previous plate were counted immediately after transfer. Two to three days later, the number of L4/adults hatched and developed from the eggs was determined.

### *C. elegans* L1 survival assay

Eggs were isolated from gravid adult nematodes by hypochlorite treatment and suspended in M9 buffer at a density of 1000 eggs/ml. The liquid culture was incubated at 20 °C and shaken at 180 rpm without the addition of food. Every day for the first week, and afterwards, every 2–3 days, a 50 µl aliquot of the liquid culture was placed on an NGM/OP50 plate. The number of L1 larvae was immediately counted, and after 2–4 days at 20 °C, the number of larvae that developed past the L1 stage was analysed.

### *C. elegans* adult lifespan assay

Nematodes were synchronized by hypochlorite treatment and allowed to grow on NGM/OP50 plates until the L4 larval stage. L4 stage larvae were then transferred to NGM/OP50 plates supplemented with 5 mg/l 5-Fluoro-20-deoxyuridin (FUdR, Sigma–Aldrich, F0503). One hundred nematodes (5 plates with 20 nematodes each) were counted for every strain and set.

Knockdown experiments in *C. elegans* were conducted using RNAi ORF constructs cloned into the pL4440-DEST vector (Horizon Discovery) and transformed into *E. coli* HT115(DE3) feeder bacteria, expressing double stranded (ds)RNA against the following target mRNAs: unc-51 (RCE1182-202301424, CloneId: Y60A3A.1), mml-1 (RCE1182-202299302, CloneId: T20B12.6). Bacterial cultures were prepared by streaking bacteria from the glycerol stock onto LB plates containing ampicillin (100 µg/ml) and tetracycline (12.5 µg/ml). After single colony picking, cultures were maintained in liquid LB medium with antibiotics. Bacterial cultures were seeded onto NGM plates supplemented with 5 mg/l 5-Fluoro-20-deoxyuridin (FUdR, Sigma-Aldrich, F0503), 25 µg/ml Carbenicillin (AppliChem, A1491) and 1 mM Isopropyl-β-D-1-thiogalactopyranosid (IPTG, Sigma-Aldrich, I6758). Nematodes were synchronized by hypochlorite treatment and allowed to grow on NGM/OP50 plates until the L4 larval stage. L4 stage larvae were then transferred to NGM/HT115 plates. Here, 100 nematodes (4 plates with 25 nematodes each) were counted for every strain.

The day of transfer to FUdR-containing plates was considered day 0 of the lifespan. The nematodes were kept at 20°C and counted every 2–3 days. Nematodes were scored dead when they did not respond to gentle prodding with a platinum wire and were then removed from the plate. Missing/dried worms on the plastic of the plate were excluded. Basic survival analysis was performed using OASIS 2^101^.

### *C. elegans* imaging

Eggs from nematodes were extracted via hypochlorite treatment and seeded onto NGM/OP50 plates. After 6 and 30 hours, nematodes were washed off the plates with M9 and anaesthetized with 0.1 M NaN_3_. L1 Larvae were subsequently mounted onto glass slides with pads of 2.5% agar solution. Images of L1 larvae were acquired using a Zeiss Axiovert 200 M microscope with a 63×1.4 DIC Plan-Apochromat oil-immersion objective. Puncta number and size were quantified using CellProfiler (Version 4.2.1).

### RNA extraction and qPCR from *C. elegans*

RNA extraction was performed using a TRIzol/RNeasy hybrid protocol^102^. Eggs were isolated by hypochlorite treatment from gravid adults on 4 10 cm NGM/OP50 plates, and the eggs were dispersed on 15 cm NGM/OP50 dishes. Two days later, L4 larvae were distributed onto NGM/FUdR/OP50 plates (day 0 of lifespan), 1000 nematodes for day 1 and 2000 nematodes for day 11 RNA extraction. On the day of extraction, nematodes were harvested in M9 and washed twice. The nematodes were frozen in liquid nitrogen and quickly thawed to room temperature. RNA was extracted using TRIzol Reagent (Life Technologies, 15596026) as follows. TRIzol Reagent was added to the animals, followed by 15 min of incubation while vortexing repeatedly. Thereafter, chloroform was added, followed by 3 min of incubation, again while vortexing repeatedly. After centrifugation for 15 min at 12,000 × *g*, the aqueous phase was transferred to a new vessel and isopropanol was added and the mixture incubated for 10 min. Then, the mixture was centrifuged at 12,000 × *g* for 10 min, the supernatant discarded, and the pellet resuspended in 75% ethanol. Finally, the RNA was pelleted by centrifugation at 7500 × *g* for 5 min and the supernatant removed. Afterwards, the RNA was resuspended in 100 µl nuclease-free water and further purified using the RNeasy Mini Kit and RNase-free DNase Set (QIAGEN). The RNA concentration was determined using a Nanodrop 1000, and cDNA was prepared from 350 ng/sample in a 30 µl reaction using TaqMan Reverse Transcription Reagents as follows. qPCR (10 µl reaction volume) was performed using SYBR Green PCR Master Mix (Applied Biosystems, 4309155), 2 µl of the cDNA reaction and 0.5 µM of the following primers:

PMP-3_fw 5′-GTTCCCGTGTTCATCACTCAT-3′,

PMP-3_rev 5′-ACACCGTCGAGAAGCTGTAGA-3′,

ATG-18_fw 5′-AGTGCACGTCTTCAAACTTGAG-3′,

ATG-18_rev 5′-TGACTTGGCATATACGCAGAGA-3′.

### Protein extraction from *C. elegans*

To obtain synchronous nematodes, eggs were isolated through bleaching and seeded onto NGM plates, which were kept at 20 °C until the L4 larval stage was reached. Then, the nematodes were harvested, washed twice with M9 buffer and kept in M9 buffer in liquid culture at 20 °C with shaking at 150 rpm for 18 h. Subsequently, the nematodes were washed with M9 buffer and lysed by adding hot Laemmli buffer and boiling for 10 min. Chromatin was sheared with a 26 G needle, extracts were centrifuged, and supernatants were used for Western blotting.

### Statistics and reproducibility

The statistical analysis methods used in this study are indicated in the legend for each subfigure. Statistical tests and graphs were performed with GraphPad Prism 9.2.0. Data are presented as mean ± standard deviation (SD) in bar graphs or scatter plots. Normal distribution of the data sets was assessed using normality tests and QQ plots. The following parametric tests were applied to data with normal distribution. Two-tailed heteroscedastic *t*-test (Welch’s *t* test) was used for pairwise comparisons. One-way ANOVA was used for multiple comparisons with one factor, followed by the Dunnett’s or Holm-Sidak post-hoc testing. Two-way ANOVA with Dunnett’s or Tukey’s post hoc test was used for multiple comparisons with more than one factor. When datasets were not normally distributed, they were logarithmically transformed (X log (Y) or x log (Y + 1)) and when they fit a Gaussian distribution, parametric tests were run using the transformed data. In this case, the graphs presented in this study display original data with statistical analysis resulting from transformed data^103^. If Gaussian distribution was not achieved through logarithmic transformation, Kruskal–Wallis testing was conducted for multiple comparisons.

### *C. elegans* lifespan assays were quantified by employing OASIS 2

The following *P* values were considered significant: *p < 0.05; **p < 0.01; ***p < 0.001.

### Reporting summary

Further information on research design is available in the Nature Portfolio Reporting Summary linked to this article.

### Supplementary information

Peer Review File

Supplementary Information

Description of Additional Supplementary Files

Supplementary Data 1

Supplementary Video 1

Supplementary Video 2

Supplementary Video 3

Supplementary Video 4

Supplementary Video 5

Reporting Summary

## Data Availability

Source data for Figures and Supplementary Figures are provided as Supplementary Data File (Supplementary Data 1) and uncropped Western blots are provided in Supplementary Fig. 9 along with this article. Newly generated plasmids have been deposited to Addgene: pGL4.23-WIPI1promotor (Addgene ID 206840), pGL4.23-5xE-box (Addgene ID 206841) and mass spectrometry proteomics data to the ProteomeXchange Consortium via the PRIDE partner repository with the dataset identifier PXD023146.
