# Supplementary material for: The ABL-MYC axis controls WIPI1-enhanced autophagy in lifespan extension
Source: Commun Biol. 2023 Aug 24;6:872. doi: 10.1038/s42003-023-05236-9 (PMC10449903; doi:10.1038/s42003-023-05236-9)
Supplement: Supplementary file 2 — Supplementary Information [file 42003_2023_5236_MOESM2_ESM.pdf]

Supplementary Information

The ABL-MYC axis controls WIPI1-enhanced autophagy in lifespan extension

Katharina Sporbeck[1,2], Maximilian L. Haas[1,#], Carmen J. Pastor-Maldonado[1,#], David S. Schüssele[1,#], Catherine Hunter[1,2], Zsuzsanna Takacs[1,2,†], Ana L. Diogo de Oliveira[1], Mirita Franz-Wachtel[3], Chara Charsou[4,5], Simon G. Pfisterer[1,§], Andrea Gubas[6], Patricia K. Haller[1,2], Roland L. Knorr[7,8,9], Manuel Kaulich[6], Boris Macek[2,3], Eeva-Liisa Eskelinen[10,11], Anne Simonsen[4,5], Tassula Proikas-Cezanne[1,2,*]

[1]Interfaculty Institute of Cell Biology, Eberhard Karls University Tübingen, 72076 Tübingen, Germany.
[2]International Max Planck Research School 'From Molecules to Organisms', Max Planck Institute for Biology and Eberhard Karls University Tubingen, 72076 Tübingen, Germany.
[3]Proteome Center Tübingen, Interfaculty Institute of Cell Biology, Eberhard Karls University Tübingen, 72076 Tübingen, Germany.
[4]Institute of Basic Medical Sciences, University of Oslo, 0372 Oslo, Norway.
[5]Centre for Cancer Cell Reprogramming, Institute of Clinical Medicine, University of Oslo, 0316 Oslo, Norway.
[6]Institute of Biochemistry II, Frankfurt Cancer Institute, Goethe University Medical School, 60590 Frankfurt, Germany.
[7]Humboldt University of Berlin, Institute of Biology, 10115 Berlin, Germany.
[8]Graduate School and Faculty of Medicine, The University of Tokyo, Tokyo 113-0033, Japan.
[9]International Research Frontiers Initiative, Institute of Innovative Research, Tokyo Institute of Technology, Yokohama 226-8503, Japan.
[10]Department of Biosciences, University of Helsinki, FI-00790 Helsinki, Finland.
[11]Institute of Biomedicine, University of Turku, FI-20520 Turku, Finland.
[†]Present address: Institute of Molecular Biotechnology, A-1030 Vienna, Austria.
[§]Present address: Department of Anatomy, Faculty of Medicine, University of Helsinki, FI-00290 Helsinki, Finland.
[#]These authors contributed equally.

*Correspondence and requests for materials should be addressed to T.P.-C. (email: tassula.proikas-cezanne@uni-tuebingen.de)

Supplementary Table 1. Lifespan assessment using wild type, ABL1 and ATG-18 deficient *C. elegans* strains. The corresponding OASIS-based statistical evaluation is listed.

***C. elegans* strain**	**Number of worms**	**Mean life span (days)**	**Bonferroni P-value**
wild type (N2)	101	19.81	
abl-1(ok171)	99	26.03	0
atg-18(gk378)	100	14.53	0
abl-1(ok171); atg-18(gk378)	98	16.65	0.0018

Supplementary Table 2. Lifespan assessment using wild type and ABL1 deficient *C. elegans* strains treated with RNAi. The corresponding OASIS-based statistical evaluation is listed.

***C. elegans* strain**	**Number of worms**	**Mean life span (days)**	**Bonferroni P-value**
wild type (N2) L4440*	100	18.56	
mml-1 (RNAi)	100	19.56	0.0264 (versus wild type (N2) L4440)
abl-1(ok171) L4440*	100	21.46	0.000000054 (versus wild type (N2) L4440)
abl-1(ok171); unc-51 (RNAi)	100	15.69	0.0000051 (versus *abl-1(ok171)* L4440)

* Control RNAi, empty L4440 plasmid.

a

Increase (>0.4)
ABL1
ADCK1
ADCK5
ALPK1
BUB1
CDKL4
DDR1
EGFR
EIF2AK4
FGFR1
INSR
IRAK1
ITK
JAK1
MAP2K6
MAPK13
MAPK14
MET
MINK1
MST1R
PLK1
PLK4
PRKAR1B
PRKCD
PTK2
RIOK2
ROCK1
SGK1
TXK

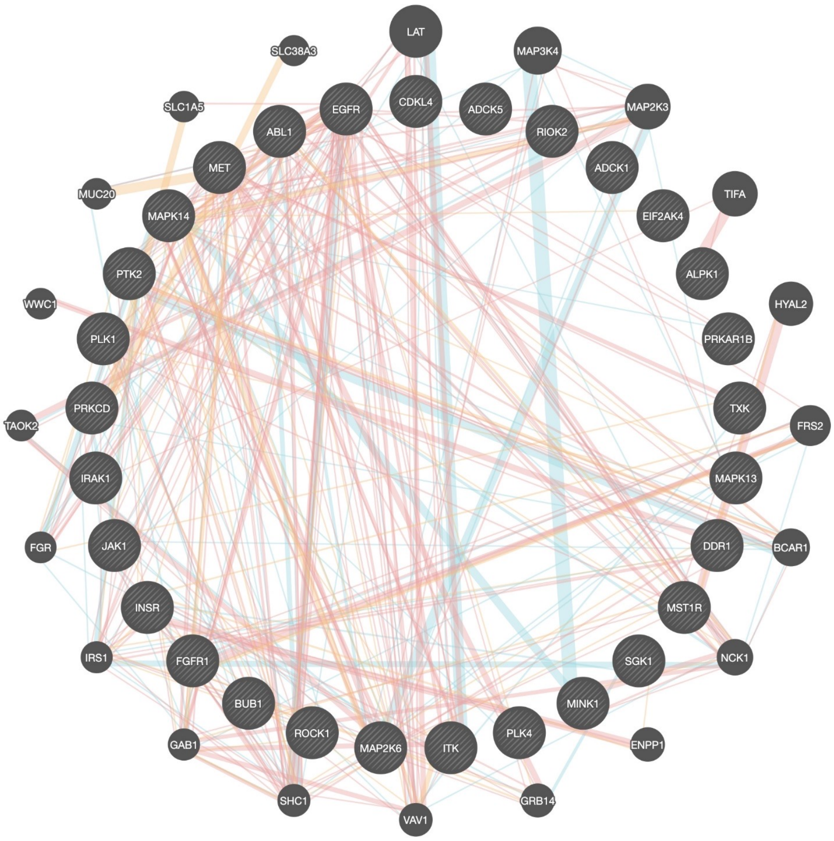

b

Decrease (<-0.4)
ATM
BUB1B
CASK
CHEK1
CSNK1G2
LMTK2
MAP2K5
MAPK1
MAPK9
MLKL
MYLK
NEK1
PCTK1
PDK1
RAF1
RPS6KA3
STK24
STK39
STK4
TGFBR1
TLK1
ULK2

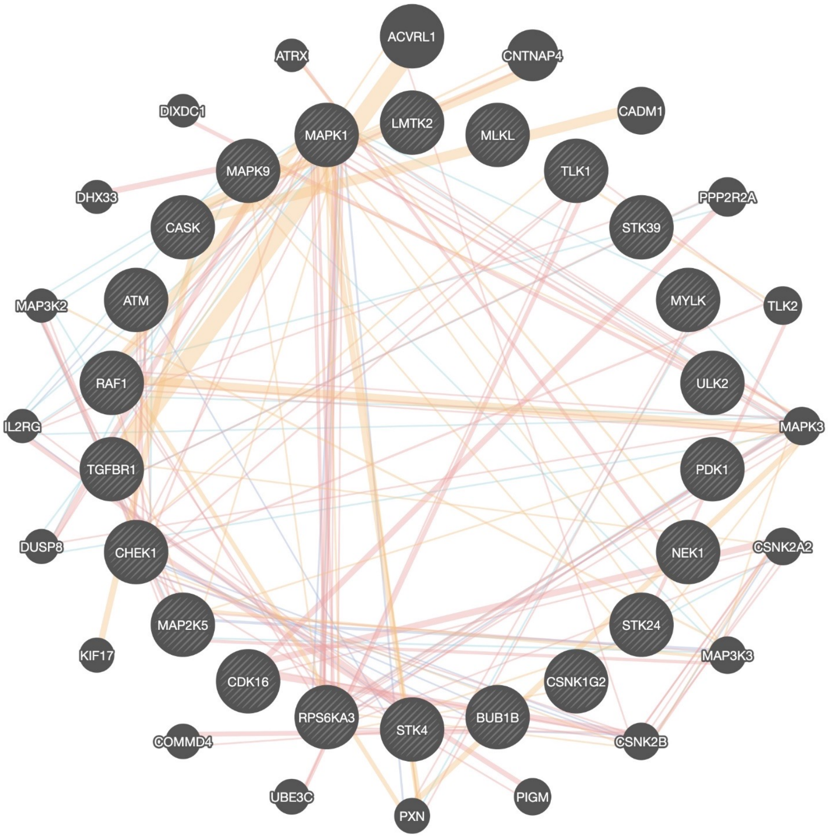

c

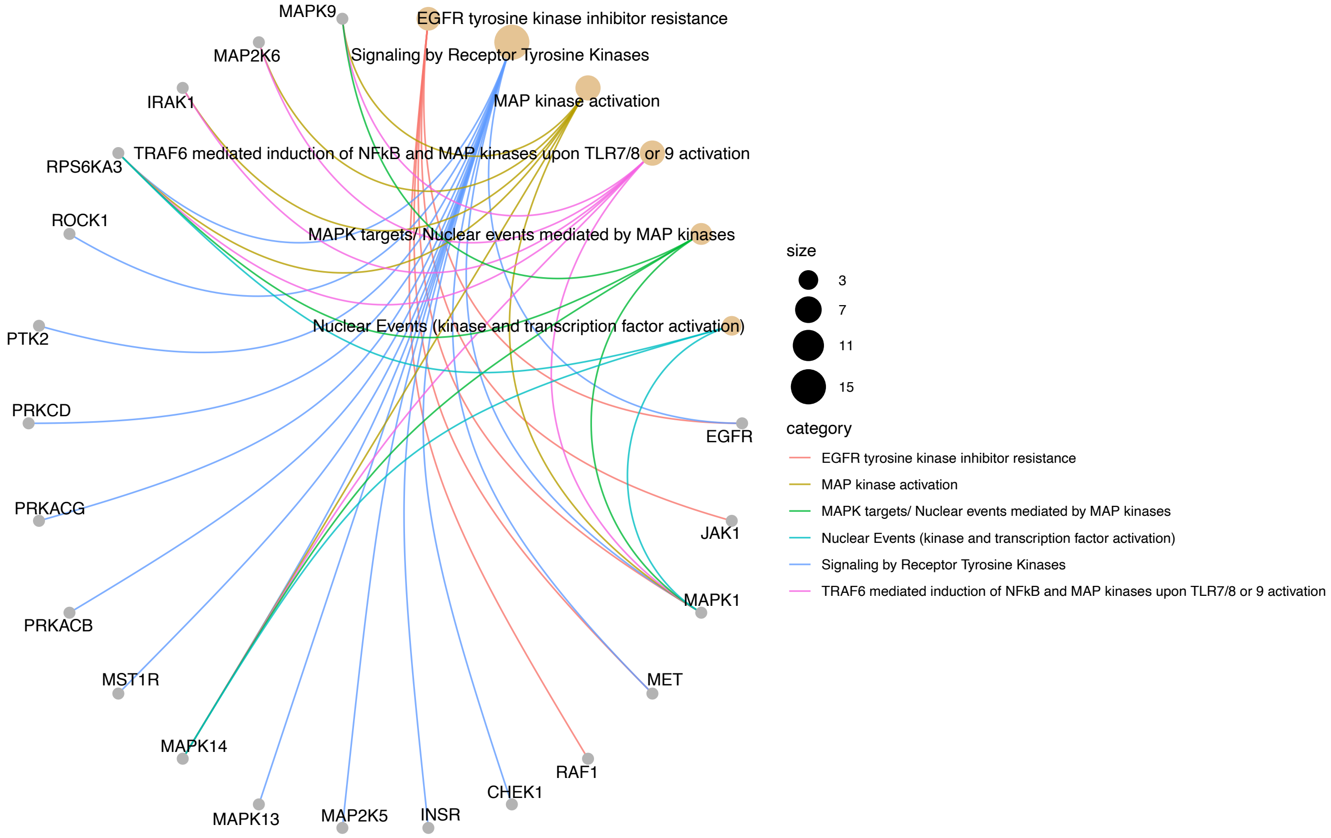

MAPK9
EGFR tyrosine kinase inhibitor resistance
MAP2K6
Signaling by Receptor Tyrosine Kinases
IRAK1
MAP kinase activation
RPS6KA3
TRAF6 mediated induction of NFkB and MAP kinases upon TLR7/8 or 9 activation
ROCK1
MAPK targets/ Nuclear events mediated by MAP kinases
PTK2
Nuclear Events (kinase and transcription factor activation)
PRKCD
EGFR
PRKACG
JAK1
PRKACB
MAPK1
MST1R
MET
MAPK14
RAF1
CHEK1
MAPK13
MAP2K5
INSR
size
3
7
11
15
category
EGFR tyrosine kinase inhibitor resistance
MAP kinase activation
MAPK targets/ Nuclear events mediated by MAP kinases
Nuclear Events (kinase and transcription factor activation)
Signaling by Receptor Tyrosine Kinases
TRAF6 mediated induction of NFkB and MAP kinases upon TLR7/8 or 9 activation

d

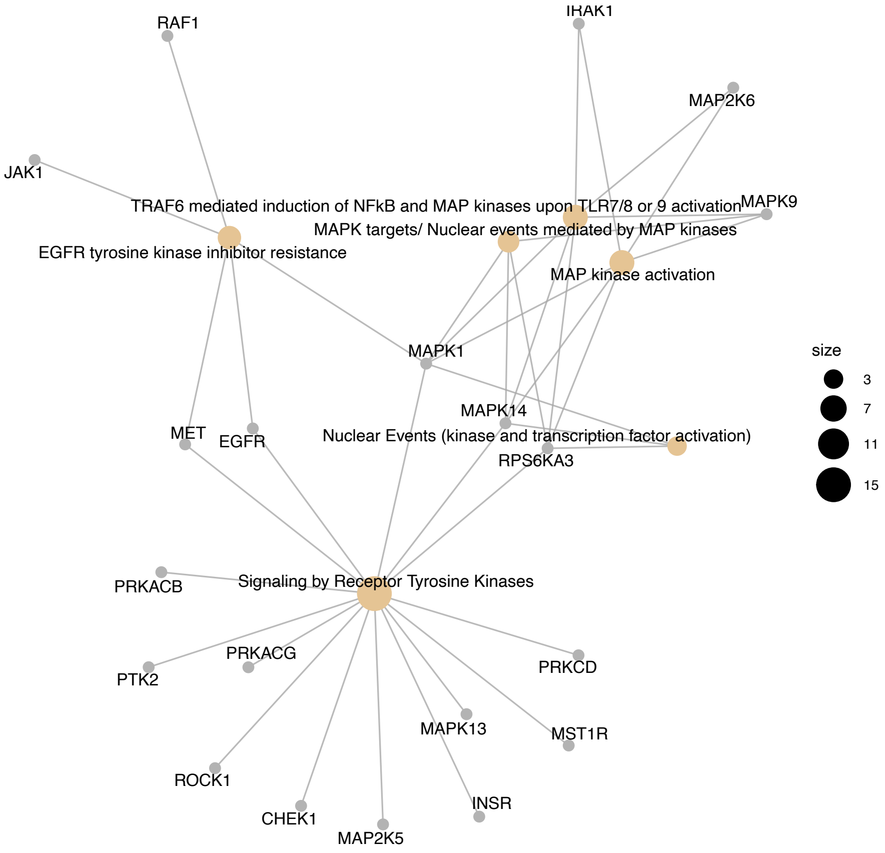

RAF1
IRAK1
MAP2K6
JAK1
TRAF6 mediated induction of NFkB and MAP kinases upon TLR7/8 or 9 activation
MAPK9
MAPK targets/ Nuclear events mediated by MAP kinases
EGFR tyrosine kinase inhibitor resistance
MAP kinase activation
MAPK1
MAPK14
MET
EGFR
Nuclear Events (kinase and transcription factor activation)
RPS6KA3
PRKACB
Signaling by Receptor Tyrosine Kinases
PRKACG
PRKCD
PTK2
MAPK13
MST1R
ROCK1
INSR
CHEK1
MAP2K5
size
3
7
11
15

Supplementary Fig. 1: Lentiviral-based GFP-WIPI1 image-based kinome screening results. (**a, b**) List of all kinases considered candidates based on having two or more shRNAs showing a fold difference >0,4 (left panels) along with their interaction profile using Genemania (right panels). **(c, d)** Pathway enrichment of all kinases (from a and b) with a difference in puncta-positive cell fold increase >0.4 is visualized as a circus plot (**c**) or network (**d**). **Supplementary** material is available (**Supplementary Data 1**).

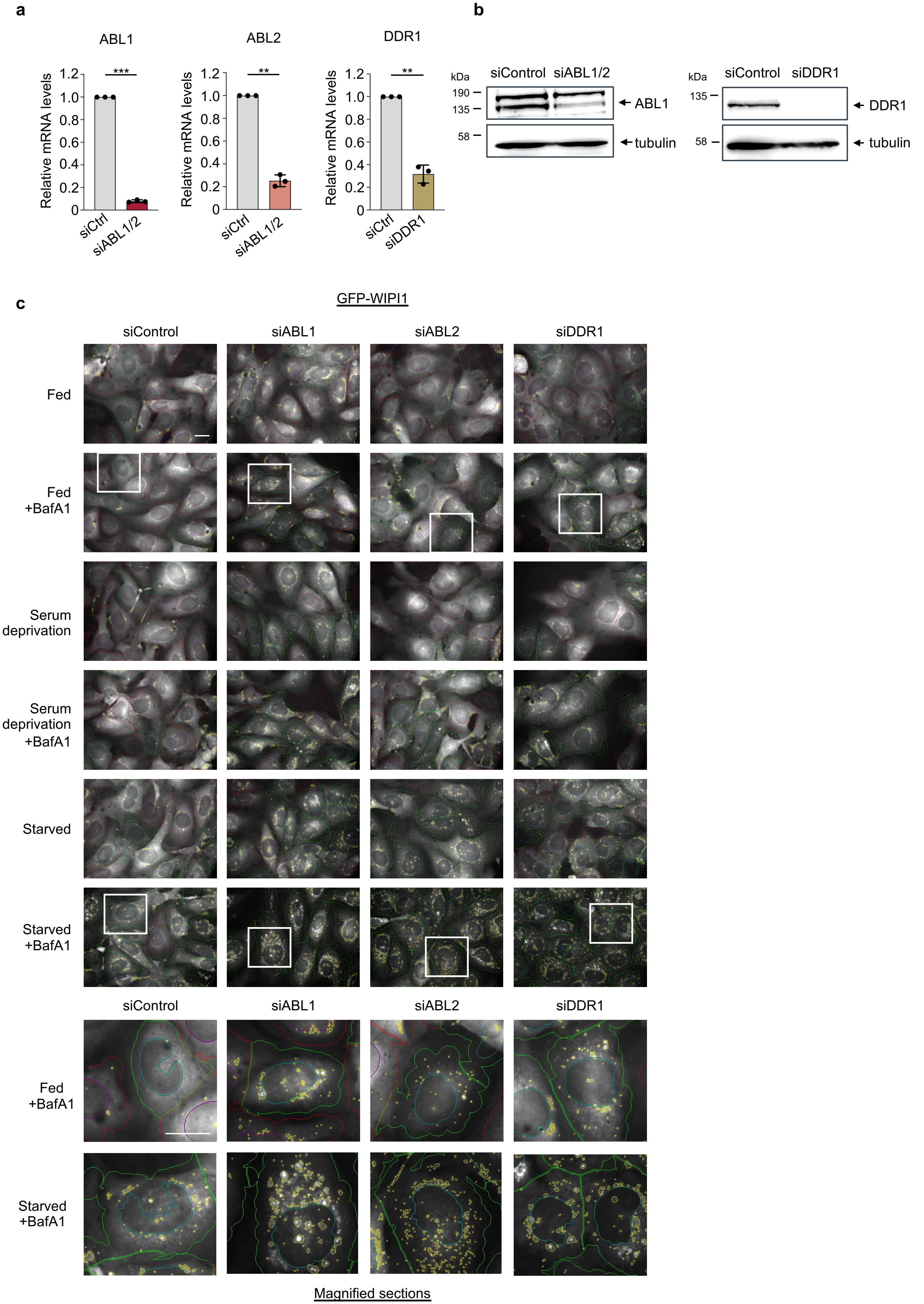

a
ABL1
ABL2
DDR1
Relative mRNA levels
1.2
1.0
0.8
0.6
0.4
0.2
0
***
**
**
siCtrl
siABL1/2
siDDR1
b
kDa
siControl
siABL1/2
190
135
58
ABL1
tubulin
siDDR1
DDR1
c
GFP-WIPI1
siControl
siABL1
siABL2
siDDR1
Fed
Fed +BafA1
Serum deprivation
Serum deprivation +BafA1
Starved
Starved +BafA1
Magnified sections

d

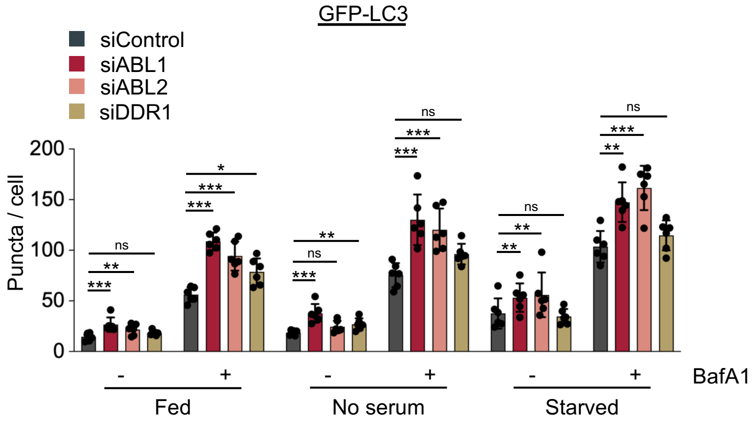
GFP-LC3
siControl
siABL1
siABL2
siDDR1
Puncta / cell
200
150
100
50
0
ns
**
***
*
***
***
**
ns
***
ns
***
***
ns
**
**
ns
***
**
-
+
BafA1
Fed
No serum
Starved

e

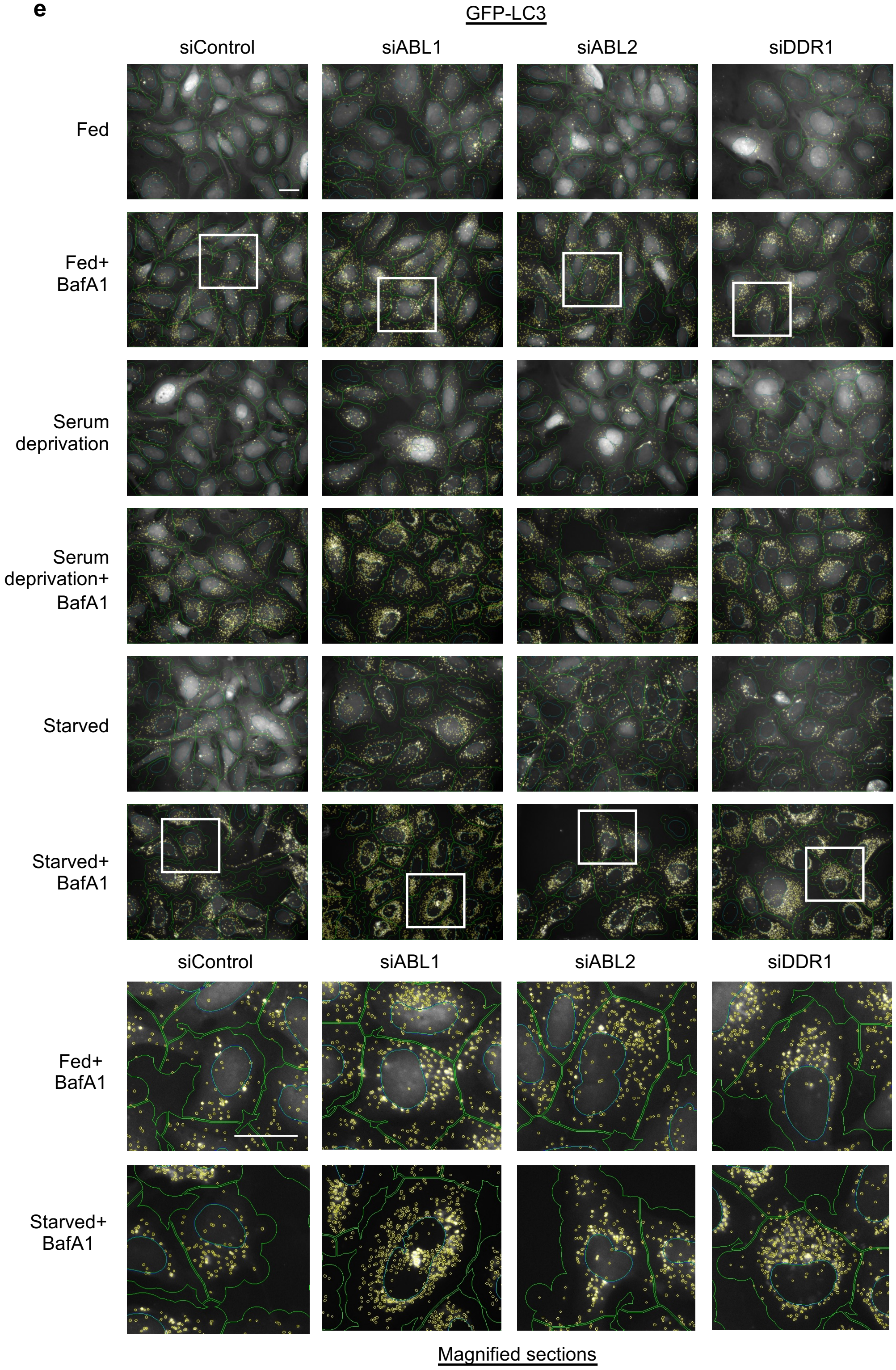
GFP-LC3
siControl
siABL1
siABL2
siDDR1
Fed
Fed+
BafA1
Serum
deprivation
Serum
deprivation+
BafA1
Starved
Starved+
BafA1
siControl
siABL1
siABL2
siDDR1
Fed+
BafA1
Starved+
BafA1
Magnified sections

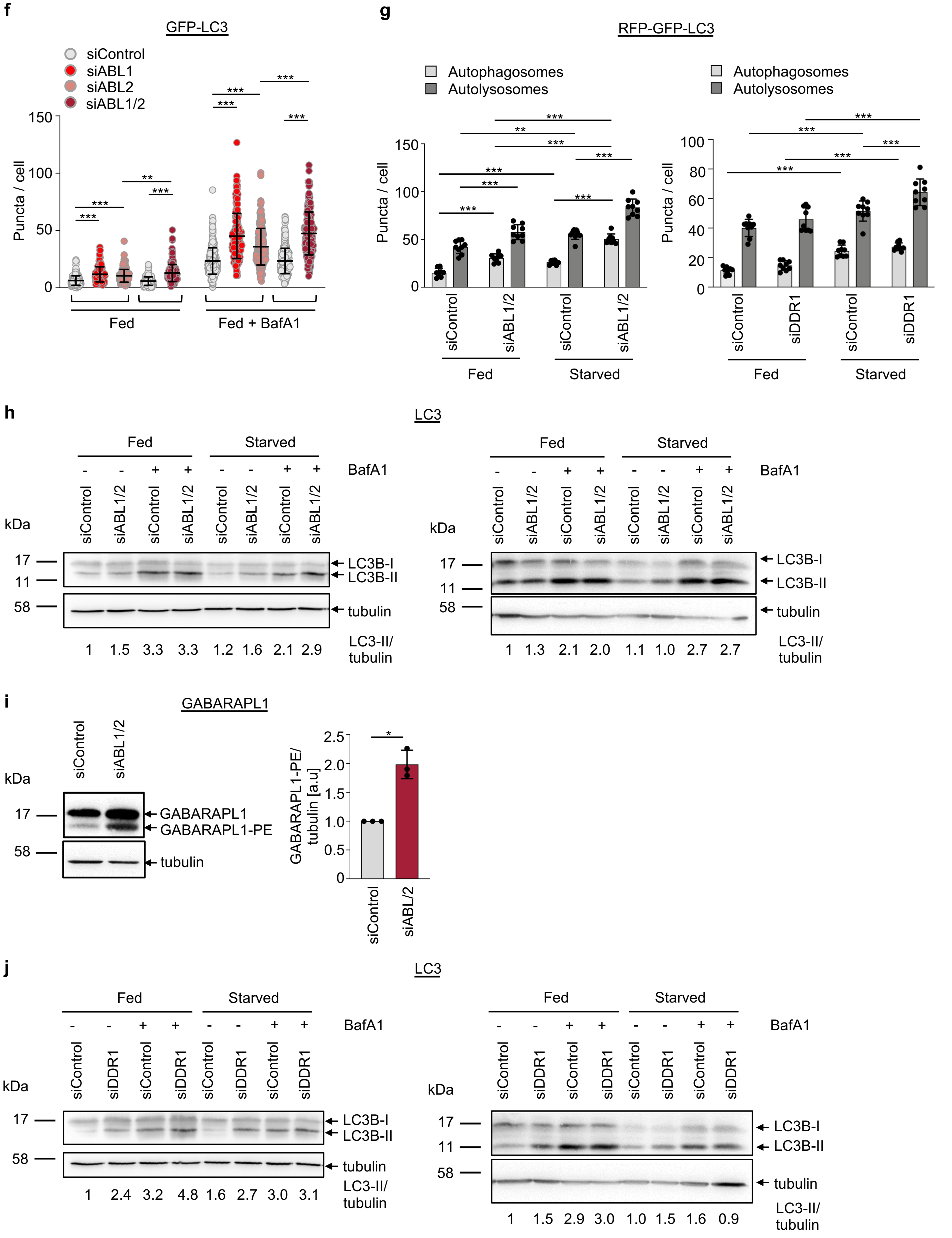
f
GFP-LC3
siControl
siABL1
siABL2
siABL1/2
Puncta / cell
150
100
50
0
Fed
Fed + BafA1
g
RFP-GFP-LC3
Autophagosomes
Autolysosomes
Puncta / cell
siControl
siABL1/2
siDDR1
Fed
Starved
h
LC3
Fed
Starved
BafA1
kDa
17
11
58
LC3B-I
LC3B-II
tubulin
1 1.5 3.3 3.3 1.2 1.6 2.1 2.9
LC3-II/ tubulin
1 1.3 2.1 2.0 1.1 1.0 2.7 2.7
i
GABARAPL1
siControl
siABL1/2
GABARAPL1
GABARAPL1-PE
tubulin
GABARAPL1-PE/ tubulin [a.u]
2.5
2.0
1.5
1.0
0.5
0
j
LC3
siControl
siDDR1
1 2.4 3.2 4.8 1.6 2.7 3.0 3.1
1 1.5 2.9 3.0 1.0 1.5 1.6 0.9

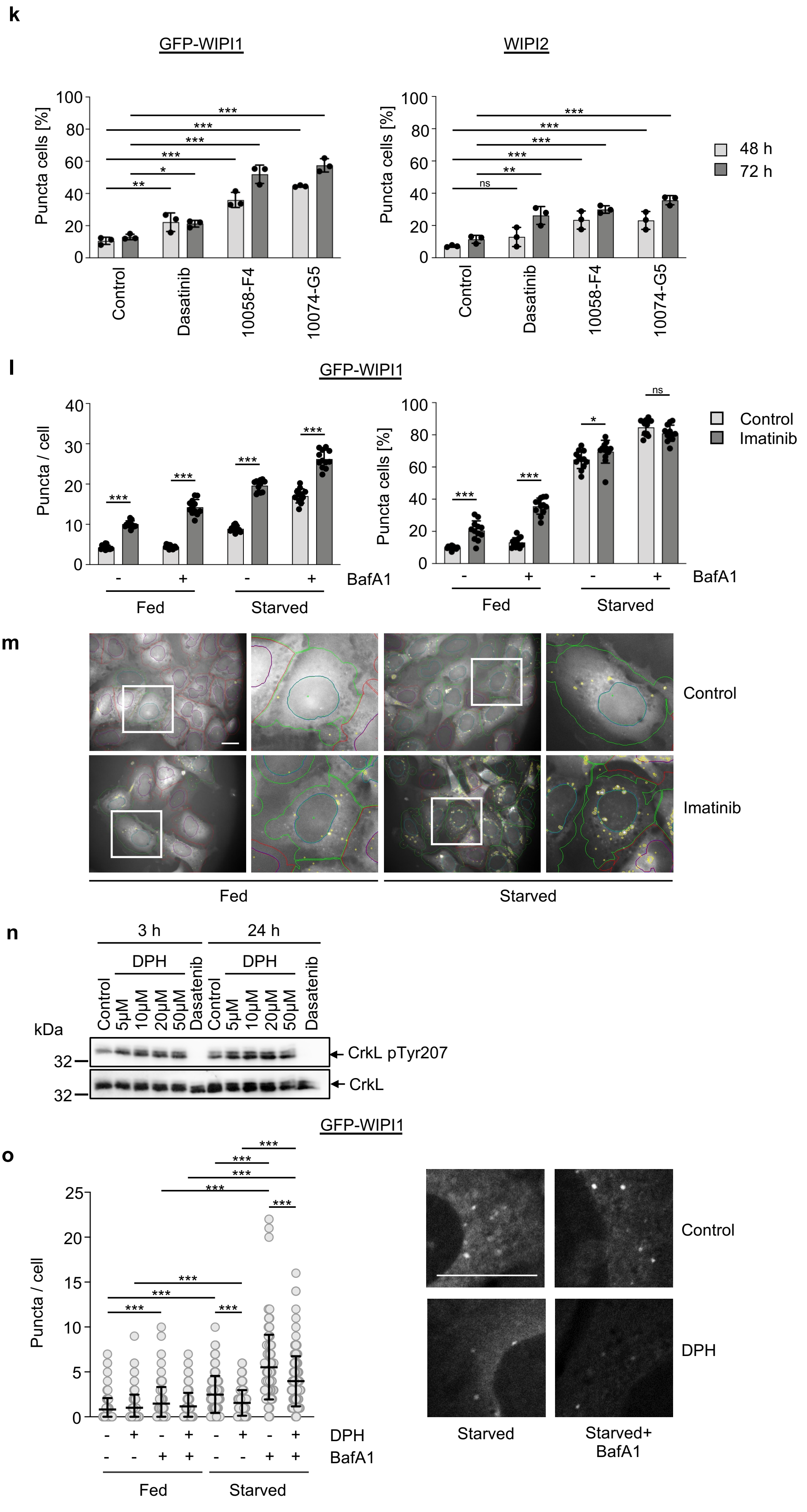
k
GFP-WIPI1
WIPI2
Puncta cells [%]
100
80
60
40
20
0
***
**
*
ns
Control
Dasatinib
10058-F4
10074-G5
48 h
72 h
l
GFP-WIPI1
Puncta / cell
40
30
20
10
0
Puncta cells [%]
Control
Imatinib
BafA1
Fed
Starved
- + - +
ns
m
Control
Imatinib
Fed
Starved
n
3 h
24 h
DPH
Control
5µM
10µM
20µM
50µM
Dasatenib
kDa
32
CrkL pTyr207
CrkL
GFP-WIPI1
o
Puncta / cell
25
20
15
10
5
0
DPH
BafA1
Fed
Starved
Control
DPH
Starved
Starved+
BafA1

Supplementary Fig. 2: ABL1, ABL2 and DDR1 inhibit autophagy. (a) ABL1 and ABL2 were downregulated (siABL1/2) in U2OS cells for 48 h, and ABL1 (left panel) and ABL2 (middle panel) downregulation was confirmed by qPCR (two-tailed heteroscedastic t test, mean±SD, n=3 in duplicates). Likewise, DDR1 downregulation (siDDR1) was also confirmed by qPCR (right panel). (**b**) Where available (ABL1, DDR1) downregulation was further confirmed by Western blotting. (**c**) Image gallery related to **Fig. 1c**. White boxes indicate magnified sections displayed in the lower panels. Scale bar: 20 µm. (**d**) U2OS cells stably expressing GFP-LC3 were transfected with siRNAs targeting ABL1 (siABL1), ABL2 (siABL2), DDR1 (siDDR1) or nontargeting control siRNAs (siControl) for 48 h. Prior to automated GFP-LC3 image acquisition and analysis using the InCell Analyzer 1000, cells were incubated in control medium (fed), control medium lacking serum (no serum) or starved for 3 h, in the presence (+) or absence (-) of bafilomycin A1 (BafA1). The numbers of GFP-LC3 per single cell (threshold-based puncta segmentation) are displayed (Welch's t test, mean±SD, up to 2775 analysed cells from n=6 for each condition). (**e**) Representative images of the experiment shown in **Supplementary Fig. 2d** are presented (upper panels). White boxes indicate the magnified image sections displayed in the lower panels. Scale bar: 20 µm. (**f**) Using CellProfiler-based single cell analysis of images acquired with automated confocal laser-scanning microscopy (LSM), the number of GFP-LC3 puncta per cell (threshold-based puncta segmentation) was determined in ABL1, ABL2, or ABL1/2 KD cells in fed conditions in the presence or absence of BafA1. A two-way ANOVA with Tukey's multiple comparisons test was performed (up to 1523 cells from n=3 for each condition) and error bars show the mean±SD deviation. (**g**) U2OS cells stably expressing RFP-GFP-LC3 were transfected with siRNAs targeting ABL1/2 (siABL1/2) (left panel) or DDR1 (siDDR1) (right panel) along with nontargeting control siRNAs (siControl) for 48 h. Prior to automated image acquisition and analysis using the InCell Analyzer 1000 (threshold-based puncta segmentation), cells were incubated in control medium (fed) or starved for 3 h. Autophagosomes were defined as RFP/GFP-positive LC3 puncta, and autolysosomes were defined as RFP-only LC3-positive puncta. Two-way ANOVA with Dunnett's post hoc test, mean±SD, up to 2690 (siControl for siABL1/2), 1493 (siABL1/2), 2778 (siControl for siDDR1) and 2843 (siDDR1) analysed cells from n=6 in each condition (fed or starved). (**h**) Additional Western blotting results regarding the data displayed in **Fig. 2d** are presented. (**i**) U2OS cells were transfected with siRNAs targeting ABL1/2 (siABL1/2) or nontargeting control siRNAs (siControl) as indicated for 48 h. Protein extracts were analysed by immunoblotting against GABARAPL1 and tubulin, and a representative result (left panels) is shown. Relative quantification of GABARAPL1 protein abundance normalized to tubulin is presented (right panels, Welch's t test, mean±SD, n=3). (**j**) Additional Western blotting results regarding the data displayed in **Fig. 2e** are presented. (**k**) U2OS cells stably expressing GFP-WIPI1 were treated with 1 µM dasatinib, 10 µM 10058-F4 or 10074-G5 for 48 or 72 h, and the percentages of GFP-WIPI1 (left panels) or WIPI2 (right panels) puncta-positive cells were counted. Two-way ANOVA with Dunnett's post hoc test, mean±SD, up to 869 (GFP-WIPI1) and 867 (WIPI2) analysed cells from n=3. (**l**) Long-term treatment using imatinib. U2OS cells stably expressing GFP-WIPI1 were treated with 10 µM imatinib for 14 days prior to 3 h of imatinib treatment under fed and starved conditions in the presence (+) or absence (-) of bafilomycin A1 (BafA1). High-throughput image acquisition and analysis using the InCell Analyzer 1000 of GFP-WIPI1 was performed, and the results are expressed as GFP-WIPI1 puncta per single cell (upper left panel) or GFP-WIPI1 puncta-positive cells (upper right panel). Threshold-based puncta segmentation. One-way ANOVA with Holm-Sidak post-hoc test, mean±SD, up to 2368 analysed cells from n=4 for each condition. (**m**) Representative images of the results presented in (l) are provided. Scale bar: 20 µm. (**n**) U2OS cells were treated with the indicated amount of DPH or 1 µM dasatinib for 3 or 24 h. Proteins were extracted, and CRKL phosphorylation at Tyr207 was assessed by Western blotting. (**o**) U2OS cells stably expressing GFP-WIPI1 were

treated with 10 µM DPH for 3 h under fed and starved conditions in the presence (+) or absence of 100 nM bafilomycin A1. Single-plane images were taken using a ZEISS LSM 800 microscope (60 images per condition from n=3, 6 cells/image), and the numbers of GFP-WIPI1 puncta per single cell were analysed manually from the images (left panels, One-way ANOVA with Holm-Sidak post-hoc test). Representative image sections are displayed (right panels, scale bar: 10 µm). Supplementary material is available (**Supplementary Data 1**). P values: * $p<0.05$; ** $p<0.01$; *** $p<0.001$; ns=not significant.

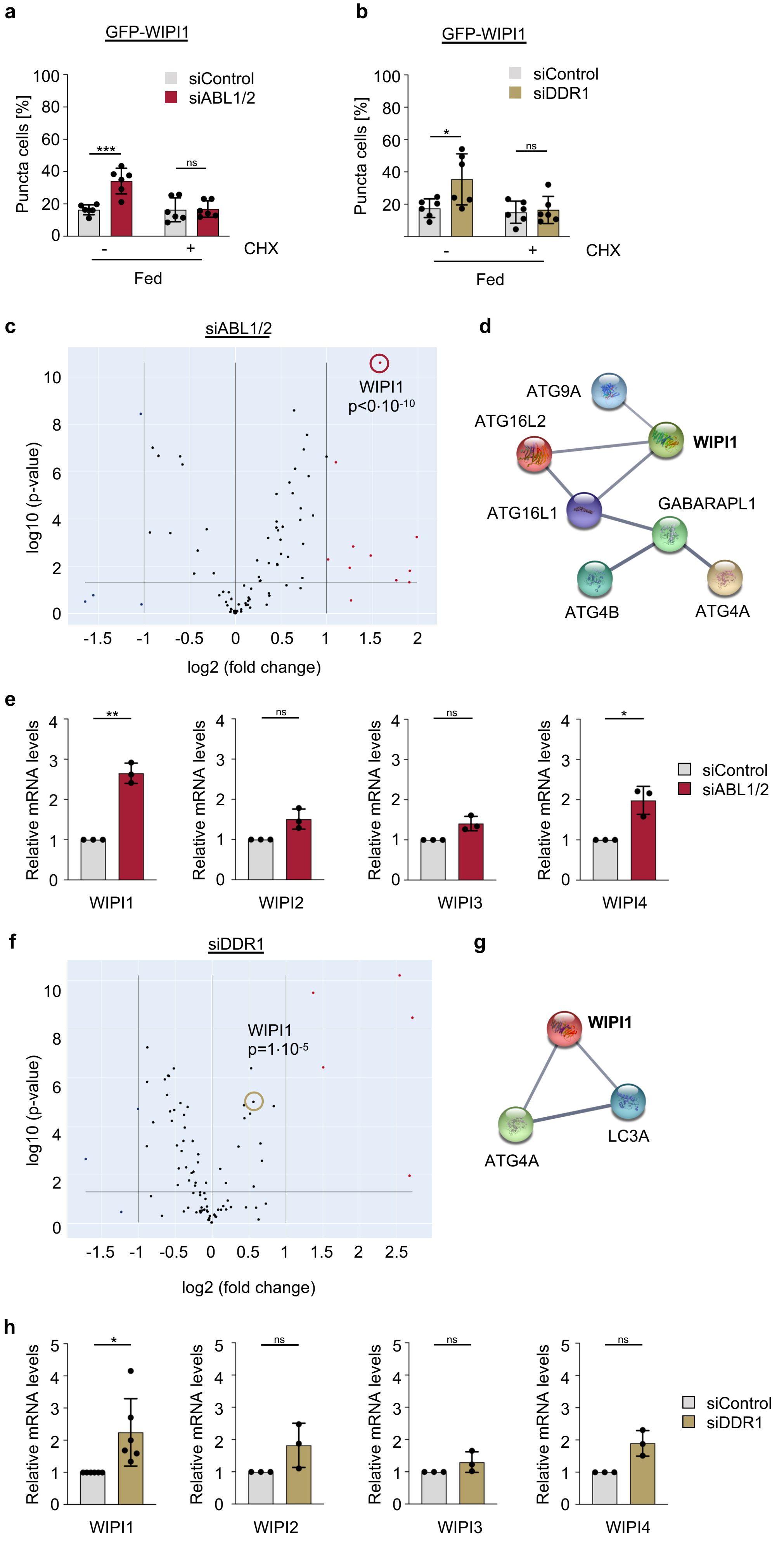
a
GFP-WIPI1
siControl
siABL1/2
Puncta cells [%]
***
ns
CHX
Fed
b
GFP-WIPI1
siControl
siDDR1
Puncta cells [%]
*
ns
CHX
Fed
c
siABL1/2
WIPI1
p<0·10-10
log10 (p-value)
log2 (fold change)
d
ATG9A
ATG16L2
WIPI1
ATG16L1
GABARAPL1
ATG4B
ATG4A
e
Relative mRNA levels
**
ns
ns
*
WIPI1
WIPI2
WIPI3
WIPI4
siControl
siABL1/2
f
siDDR1
WIPI1
p=1·10-5
log10 (p-value)
log2 (fold change)
g
WIPI1
LC3A
ATG4A
h
Relative mRNA levels
*
ns
ns
ns
WIPI1
WIPI2
WIPI3
WIPI4
siControl
siDDR1

Supplementary Fig. 3: ABL1/2 and DDR1 depletion increases WIPI1 mRNA abundance. (**a, b**) U2OS cells stably expressing GFP-WIPI1 were transfected with siRNAs targeting ABL1/2 (siABL1/2), DDR1 (siDDR1) or nontargeting control siRNAs (siControl) for 48 h as indicated. Cells were then incubated in the presence (+) or absence (-) of 10 µg/ml cycloheximide (CHX). High-throughput image acquisition and analysis of GFP-WIPI1 was performed, and the percentage of GFP-WIPI1 puncta-positive cells is given. Two-way ANOVA with Tukey's post-hoc test, mean±SD, up to 3177 (siControl for siABL1/2), 3451 (siABL1/2), 2947 (siControl for siDDR1) and 3019 (siDDR1) analysed cells from n=6 in each condition (+/- of CHX). (**c**) Volcano plot of gene expression array analysis. RNA was extracted from U2OS cells after downregulation of ABL1/2 (siABL1/2) or nontarget control (siControl) for 48 h, and ATG and ATG-related gene expression (84 autophagy genes) was profiled by qRT–PCR. Log(2) (fold change siABL1/2 over siControl) against log(10) (p value) is shown. Fold changes of mRNA >1 and p values <0,05 were considered differentially up- or downregulated (n=3 in technical duplicates). The WIPI1 data point is circled in red. (**d**) ATG genes upregulated upon ABL1/2 knockdown are visualised using the Search Tool for the Retrieval of Interacting Genes/Proteins (STRING) database. (**e**) Validation of WIPI1 upregulation by subsequent TaqMan qPCR. Relative mRNA levels of siControl and siABL1/2 are shown, also including WIPI2, WIPI3 and WIPI4 probes as indicated. Welch's t test, mean±SD, n=3 in triplicates. (**f**) Volcano plot of gene expression array analysis upon DDR1 downregulation, as in (c). (**g**) ATG genes upregulated upon DDR1 knockdown are expressed using the STRING database. (**h**) Confirmation of WIPI1 upregulation in (g), as in (e). Supplementary material is available (**Supplementary Data 1**). P values: * p<0.05; ** p<0.01; *** p<0.001; ns=not significant.

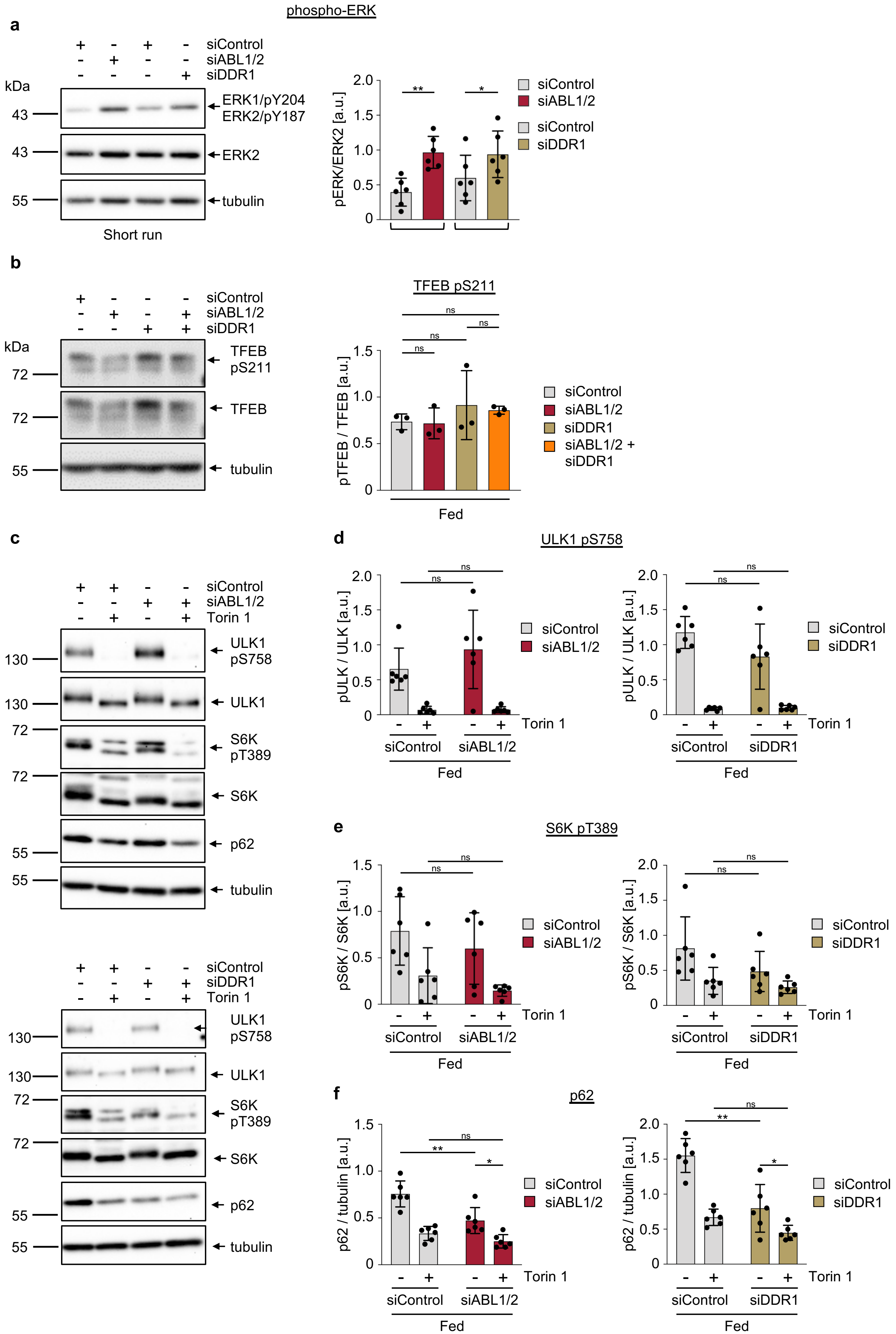

a
phospho-ERK
siControl
siABL1/2
siDDR1
kDa
43
43
55
ERK1/pY204
ERK2/pY187
ERK2
tubulin
Short run
pERK/ERK2 [a.u.]
**
*
siControl
siABL1/2
siControl
siDDR1
b
TFEB pS211
siControl
siABL1/2
siDDR1
kDa
72
72
55
TFEB pS211
TFEB
tubulin
pTFEB / TFEB [a.u.]
ns
siControl
siABL1/2
siDDR1
siABL1/2 + siDDR1
Fed
c
siControl
siABL1/2
Torin 1
130
130
72
72
55
55
ULK1 pS758
ULK1
S6K pT389
S6K
p62
tubulin
siControl
siDDR1
Torin 1
d
ULK1 pS758
pULK / ULK [a.u.]
ns
siControl
siABL1/2
siDDR1
Torin 1
Fed
e
S6K pT389
pS6K / S6K [a.u.]
f
p62
p62 / tubulin [a.u.]
**
*

g

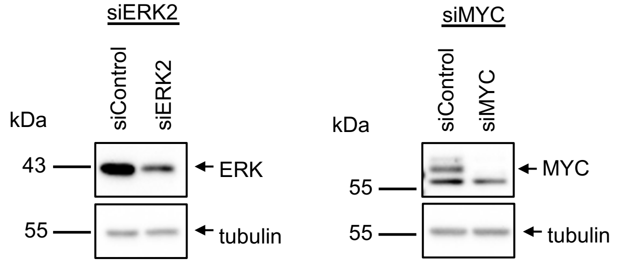

siERK2
siControl
siERK2
kDa
43
ERK
55
tubulin
siMYC
siControl
siMYC
kDa
MYC
55
55
tubulin

h

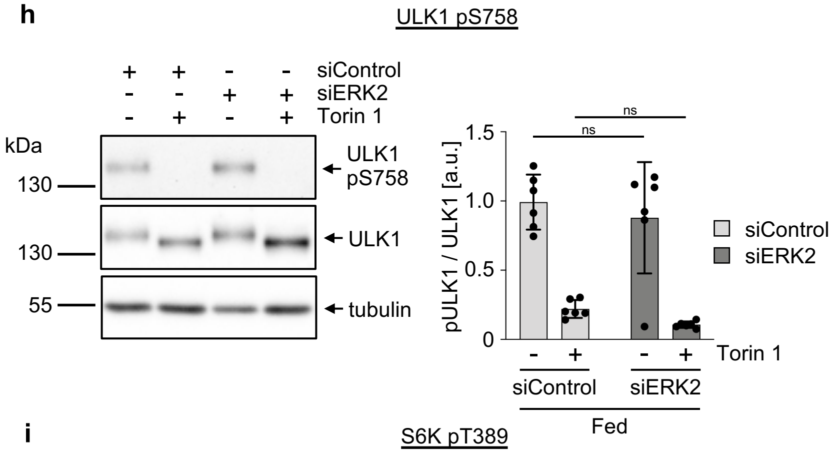

ULK1 pS758
siControl
siERK2
Torin 1
kDa
130
ULK1 pS758
130
ULK1
55
tubulin
pULK1 / ULK1 [a.u.]
1.5
1.0
0.5
0
ns
ns
siControl
siERK2
Torin 1
Fed

i

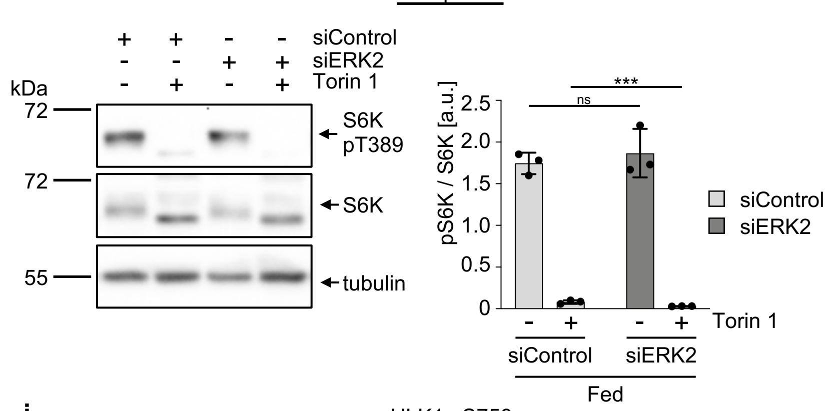

S6K pT389
siControl
siERK2
Torin 1
kDa
72
S6K pT389
72
S6K
55
tubulin
pS6K / S6K [a.u.]
2.5
2.0
1.5
1.0
0.5
0
ns
***
siControl
siERK2
Torin 1
Fed

j

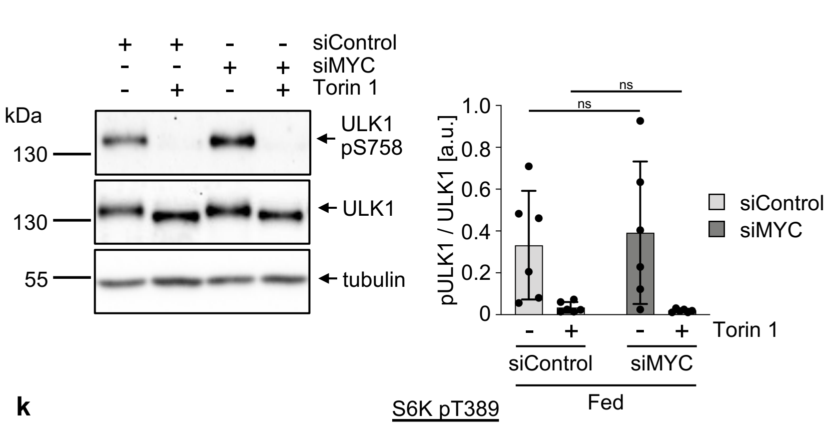

ULK1 pS758
siControl
siMYC
Torin 1
kDa
130
ULK1 pS758
130
ULK1
55
tubulin
pULK1 / ULK1 [a.u.]
1.0
0.8
0.6
0.4
0.2
0
ns
ns
siControl
siMYC
Torin 1
Fed

k

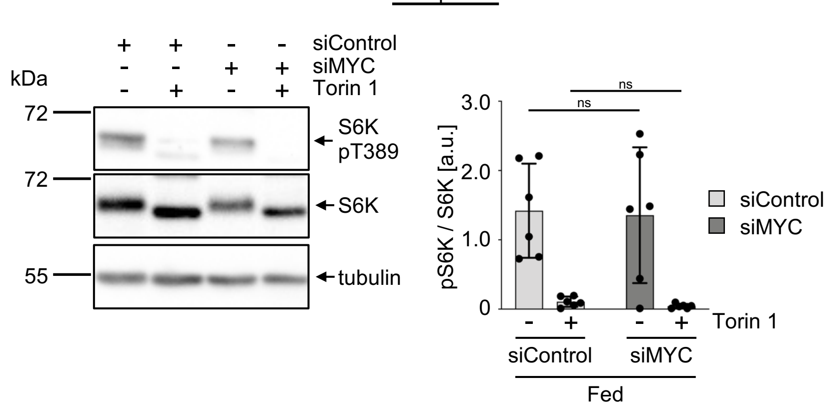

S6K pT389
siControl
siMYC
Torin 1
kDa
72
S6K pT389
72
S6K
55
tubulin
pS6K / S6K [a.u.]
3.0
2.0
1.0
0
ns
ns
siControl
siMYC
Torin 1
Fed

I

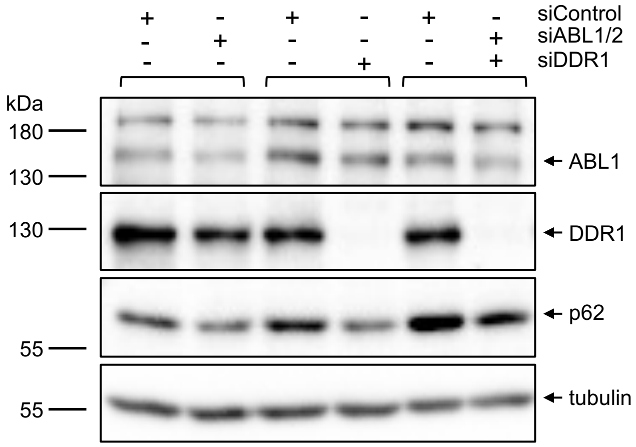
siControl
siABL1/2
siDDR1
+ - + - + -
- + - - - +
- - - + - +
kDa
180
130
130
55
55
ABL1
DDR1
p62
tubulin

Supplementary Fig. 4: The ABL-MYC axis has no impact on mTOR signalling in fed conditions used in this study. (a) U2OS cells were transfected with siABL1/2, siDDR1 or nontargeting siRNA (siControl) for 48 h, followed by quantitative immunoblotting against ERK1/2 pY204/Y187, ERK2 and tubulin (n=6, mean±SD, one-way ANOVA with Holm-Sidak multiple comparisons test). (**b**) U2OS cells were transfected with siABL1/2, siDDR1, or both siABL1/2 and siDDR1, or nontargeting siRNA (siControl) for 48 h, followed by quantitative immunoblotting against TFEB pS211, TFEB and tubulin. (n=3, left panel: representative Western blot, right panel: quantification, One-way ANOVA with Holm-Šídák's multiple comparisons test). (**c-f**). U2OS cells were transfected with siABL1/2, siDDR1 or nontargeting siRNA (siControl) for 48 h, followed by Torin 1 treatments for 3 h in fed conditions as indicated. Subsequently, quantitative immunoblotting against ULK1 pS758/ULK1, S6K pT389/S6K, p62 and tubulin was performed (n=6). Representative Western blots are displayed in (c), ULK1 pS758/ULK1 quantification in (d), S6K pT389/S6K quantification in (e), p62/tubulin quantification in (f). Two-way ANOVA with Tukey's multiple comparisons test. (**g**) U2OS cells were transfected with siERK2, siMYC or nontargeting siRNA (siControl) for 48 h, followed by protein extracts and immunoblotting against the ERK2 (left panels) or c-MYC (right panels). (**h, i**) U2OS cells were transfected with siERK2 or nontargeting siRNA (siControl) for 48 h, followed by Torin 1 treatments for 3 h in fed conditions as indicated. Subsequently, quantitative immunoblotting against ULK1 pS758/ULK1 (h, n=6) or S6K pT389/S6K (I, n=3) was performed. Left panels: representative Western blots, right panels: Two-way ANOVA with Tukey's multiple comparisons test. (**j, k**) As in (h, i) the experiment was conducted by employing siRNAs targeting c-MYC along with non-targeting siRNA controls (n=6). (**l**) U2OS cells were transfected with siABL1/2, siDDR1, siAbl1/2 + siDDR1 or nontargeting siControls for 48h. Immunoblotting was conducted against p62 and tubulin (n=3). Representative Western blot is displayed. Supplementary material is available (**Supplementary Data 1**). P values: * p<0.05; ** p<0.01; *** p<0.001; ns=not significant.

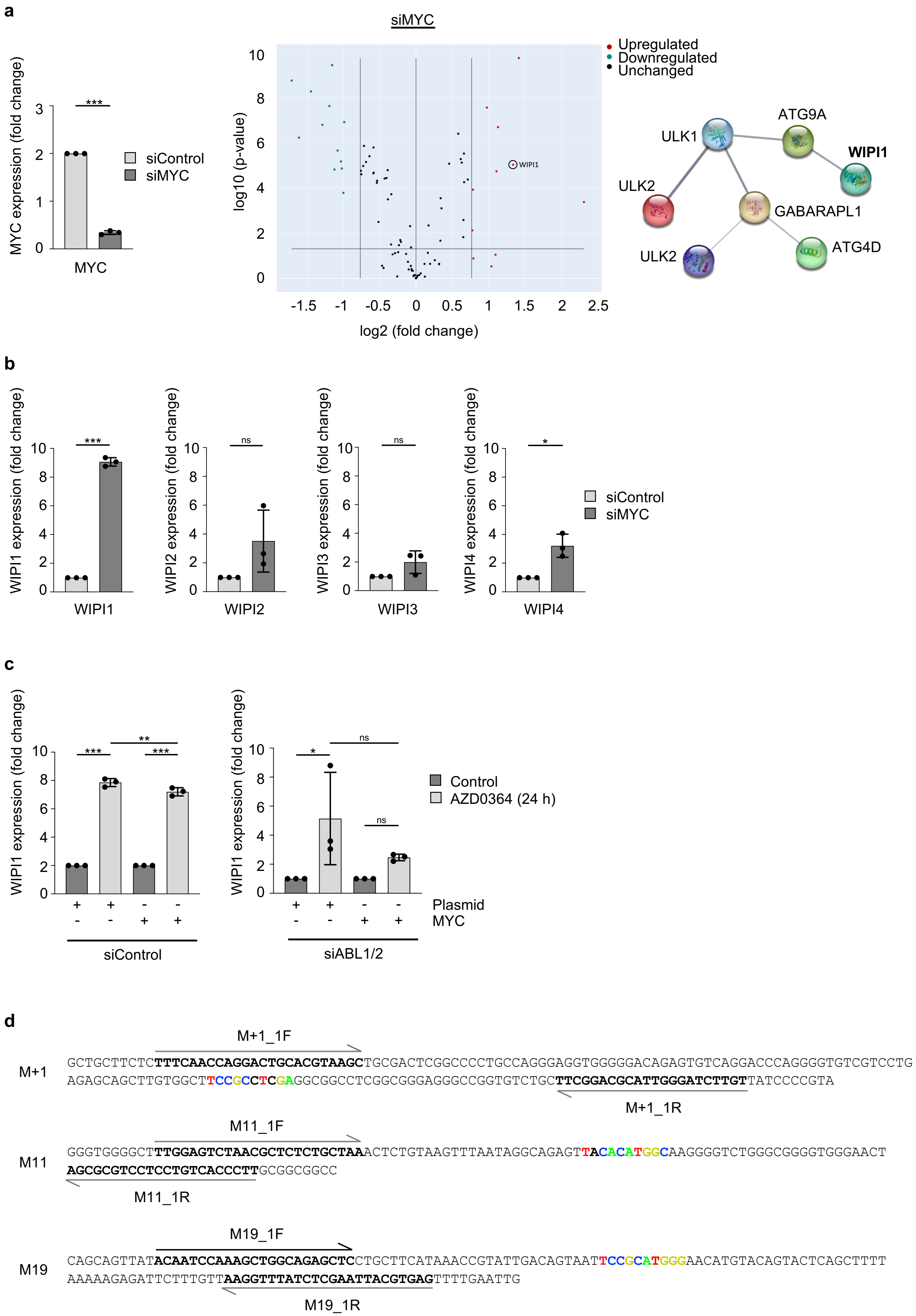

a
MYC expression (fold change)
***
siControl
siMYC
MYC
siMYC
Upregulated
Downregulated
Unchanged
log10 (p-value)
log2 (fold change)
WIPI1
ULK1
ATG9A
WIPI1
ULK2
GABARAPL1
ULK2
ATG4D
b
WIPI1 expression (fold change)
WIPI2 expression (fold change)
WIPI3 expression (fold change)
WIPI4 expression (fold change)
***
ns
ns
*
WIPI1
WIPI2
WIPI3
WIPI4
siControl
siMYC
c
WIPI1 expression (fold change)
***
**
***
*
ns
ns
Control
AZD0364 (24 h)
Plasmid
MYC
siControl
siABL1/2
d
M+1_1F
M+1
GCTGCTTCTCTTTCAACCAGGACTGCACGTAAGCTGCGACTCGGCCCCTGCCAGGGAGGTGGGGGACAGAGTGTCAGGACCCAGGGGTGTCGTCCTG
AGAGCAGCTTGTGGCTTCCGCCTCGAGGCGGCCTCGGCGGGAGGGCCGGTGTCTGCTTCGGACGCATTGGGATCTTGTTATCCCCGTA
M+1_1R
M11_1F
M11
GGGTGGGGCTTTGGAGTCTAACGCTCTCTGCTAAACTCTGTAAGTTTAATAGGCAGAGTTACACATGGCAAGGGGTCTGGGCGGGGTGGGAACT
AGCGCGTCCTCCTGTCACCCTTGCGGCGGCC
M11_1R
M19_1F
M19
CAGCAGTTATACAATCCAAAGCTGGCAGAGCTCCTGCTTCATAAACCGTATTGACAGTAATTCCGCATGGGAACATGTACAGTACTCAGCTTTT
AAAAAGAGATTCTTTGTTAAGGTTTATCTCGAATTACGTGAGTTTTGAATTG
M19_1R

e

M11*

```
human/1-77         1 gggtgacaggaggacgcgctagt-tcccaccccgcccagaccccttgccatgtgtaac57
chimp/1-77         1 gggtgacaggaggacgcgctagt-tcccaccccgcccagaccccttgccatgtgtaac57
gorilla/1-77       1 gggtgacaggactacgcgctagt-tcccaccctgcccagacgccttgccatgtgtaac57
orangutan/1-77     1 gggtgacaggaggacgcgctagt-tcccaccccgcccagaccccttgccatgtgtaac57
rhesus/1-77        1 gcggggcaggaggacgcgctagt-tcccaccccgcccagaccccttgacatgtgtaac57
baboon/1-77        1 gcggggcaggaggacgcgctagt-tcccaccccgcccagaccccttgacatgtgtaac57
marmoset/1-77      1 ggaagacgggaggacgcgctagt-tcccactccgcccaaaccctttgccaagtgtaac57
mouse-lemur/1-62   1 g--ggacagaccgacgcgctagc-tcctactccacccaaacccg-ggcagtgtgtgtc54
bushbaby/1-76      1 gctggacaggcggacgcgctagc-tcctgcttcacccgagctcc-tgccgtgtgcggc56
treeshrew/1-61     1 aggggacgggcggacgcactcgc-tcccgccccacccaacccccc--------------43
mouse/1-48         1 aggaggcgggcggacgcctg------cttccttgccc---------------------31
rat/1-48           1 aggaggcgggtggacgcccg------cttccccgccc---------------------31
kangaroo-rat/1-55  1 tgggaacaggcgtgcgtatgggt-ttcttctccaccc---------------------36
guinea-pig/1-76    1 aggagactggcggacgcgctcgt-tccctccccacccaattctt---taatgtgtgac54
squirrel/1-77      1 -ggacacaggcggacgcgctgct-tcccacccccacccaaacccc-ggtcatgtgcaac55
megabat/1-78       1 aggggactagcggacgcgctggt-tcctactccacccaacccct-cgtcatgtgtacg56
dog/1-79           1 ccagggcgggcggacgcgctcgg-tctcaccccgcccagaccgc-tgtcaccagtagc56
horse/1-74         1 acgggacaggcagacgcgctcgt-taccaccccacccaaagccc-cgtcatgtgtgac56
hedgehog/1-77      1 aggggaccggctgacgcgctcagatcccatcccacccaacccct-agtcatgtgaaa-56
shrew/1-78         1 aggggacggactgacgcgctcct-tcccaccccgcccagatccc-cgtcacgcgtagc56
microbat/1-77      1 aggggacaggcggacgctctcgt-tcctaccccacccagtcgc--cggcatgtgtaac55
dolphin/1-77       1 agcagaccggcgggcgcgctcgt-tccgaccccacccagccccg-tgtcctgggtaac56
rock-hyrax/1-88    1 aggggacaagtggacgcgctcct-t-ccaccccacccaagctgt-tgtcatgtgtga-54
tenrec/1-74        1 agaggacgggcggacgcgcttct-ttccaccccacccaa-ctcc-tgtcctgagtaac55
elephant/1-77      1 agggaacaggcggacgcgctcct-tcccaccccacccaaacccc-tgtcaggtgtga-55
armadillo/1-83     1 ----gacaggttgaagtgcttgt-ttccccgcccccggacccg-tgtcctaagtagc52
sloth/1-91         1 aggggacagccggacgcgcacgt-ttccatcccacccaaacccc-tgtcatgtgtagc56

human/1-77        58 tct--gcctattaaactt-------------acag                       77
chimp/1-77        58 tct--gcctattaaactt-------------acag                       77
gorilla/1-77      58 tct--gcctattaaactt-------------acag                       77
orangutan/1-77    58 tct--gcctattaaactt-------------acag                       77
rhesus/1-77       58 tct--gcctgttaaactt-------------acag                       77
baboon/1-77       58 tct--gcctgttaaactt-------------acag                       77
marmoset/1-77     58 tct--gcctattaaactt-------------acag                       77
mouse-lemur/1-62  55 tct------------cac-------------ag--                       62
bushbaby/1-76     57 tgtctgcctattaaactt-------------ac--                       76
treeshrew/1-61    44 --t--gccatgtacactt-------------acag                       61
mouse/1-48        32 -----gcccgctaagctt-------------acag                       48
rat/1-48          32 -----gcccgctaagcat-------------acag                       48
kangaroo-rat/1-55 37 ---ttactcccgaccggg-------------gcag                       55
guinea-pig/1-76   55 tgtctgccaattaaattt-------------acag                       76
squirrel/1-77     56 cctttgcctattaaactt-------------acgg                       77
megabat/1-78      57 tcttcgtctattaaactc-------------acag                       78
dog/1-79          57 tctctgcctattaaactt------------aacag                       79
horse/1-74        57 ttt----ctattcaacct-------------acag                       74
hedgehog/1-77     57 -ccccgcctgttaaactt-------------acag                       77
shrew/1-78        57 tctccgtttattaaactg-------------acag                       78
microbat/1-77     56 tcttggtctattaaattt-------------acag                       77
dolphin/1-77      57 actccgtctactaaactt-------------aca-                       77
rock-hyrax/1-88   55 -cggtgcgtgtttaacttcttaggggagcgcacag                       88
tenrec/1-74       56 tcgctgcgtaatatgcta------------c----                       74
elephant/1-77     56 -ctctgcatattaagctt------------cgcag                       77
armadillo/1-83    53 tct----ctattgaacttcttaaggaaacagccgg                       83
sloth/1-91        57 tctctgcctattaaacttcttaagggagcaactgg                       91
```

* Orientation: reverse complimentary

f

M19*

```
human/1-102         1 gaatctcttttttaaaagctgagtactgt-acatg---------ttcc-cat-gcg- 43
marmoset/1-102      1 ggatctctttttccaggctaagtactgt-acatg---------tttc-cat-gcg- 43
baboon/1-102        1 ggatctctttttcaagctaagtactgt-acata---------tttc-cat-gtg- 43
chimp/1-102         1 gaatctcttttttaaaagctgagtactgt-acatg---------ttcc-cat-gcg- 43
gorilla/1-102       1 gaatctcttttttaaaagctgagtactgt-acatg---------tttc-cat-gcg- 43
orangutan/1-102     1 gaatctcttttttaaaagctaagtactgt-acatg---------ttcc-cat-gcg- 43
bushbaby/1-101      1 a-atccccttcccgagctaagtattac-actta---------ttgc-cat-ata- 42
mouse-lemur/1-103   1 agatcccctttcccaaggtaagtactgt-acata---------ttgc-cac-ata- 43
treeshrew/1-92      1 ggatctctttccccaagctgaagactgt-gcatgttgccacattgc-cat-act- 51
guinea-pig/1-95     1 gatcttttatccttaggccaa-----at-gcatg---------ttgc-cat-gta- 38
squirrel/1-99       1 gattttttcttccccaggctaa-----at-gtgtg---------ttgcacct-gtat 40
cow/1-94            1 gggtctctcttcccaacctaa-----at-acatg---------ttgc-cat-t--- 36
dolphin/1-95        1 cggtctctcttcccaagatga-----at-acatg---------ttgc-cat-gta- 38
horse/1-95          1 gagtctctctgcccaagctaa-----at-gcatg---------ttgc-cat-gga- 38
dog/1-75            1 ggat----------------------------catg---------ttgc-cat-gta- 18
microbat/1-105      1 gggtctctgttcccaaggtga-----aa-acatgc--------tggc-cat-gta- 39
megabat/1-97        1 gggtcgctcttcccaagctat-----aa-ctatact-------ttgc-cct-gca- 40
hedgehog/1-83       1 -------tcttcccaaatcaa-----atcacata---------ttgc-cgt-gta- 32
shrew/1-90          1 -agtctctcttcccaaggtca-----atcagccg---------tttc-cat-gta- 38
rock-hyrax/1-99     1 ggatttctgttcccaagctaa-----aa-gcctg---------ttg--tat-gta- 37
elephant/1-103      1 ggatttctgttcctaaatcaa-----at-gcata---------ttgc-caa-gta- 38
sloth/1-99          1 ggatttcggttcccaagctaa-----at-acatg---------ttgc-cataata- 39
tenrec/1-98         1 agatttctgttgccaagctaa-----at-gcatg---------ttgc-cgt-gcc- 38

human/1-102        44 -gaatta-ctgtcaa----tacggtttatgaagc----------aggagctctgcc 83
marmoset/1-102     44 -gaatta-ttgtcag----tatggtttatgaatc----------aggagctctgcc 83
baboon/1-102       44 -gaatta-ttgtcaa----tacggtttatgaatc----------aggcgctctgcc 83
chimp/1-102        44 -gaatta-ctgtcaa----tacggtttatgaagc----------aggagctctgcc 83
gorilla/1-102      44 -gaatta-ctgtcaa----tacggtttatgaagc----------aggagctctgcc 83
orangutan/1-102    44 -gaatta-ctgtcaa----tacggtttatgaagc----------aggagctctgcc 83
bushbaby/1-101     43 -gaactg-tcatcga----cacagtttatggacc----------aggagcta-tct 81
mouse-lemur/1-103  44 -gaatta-tcatcaa----cacagtgtatgaatc----------aggagctcttct 83
treeshrew/1-92     52 -gattta-ccgccta----ca-ggcttggggata----------aagagctctgcc 90
guinea-pig/1-95    39 --aatta-ctctc------aacagtttgcgaatt----------aaaagacttact 75
squirrel/1-99      41 ggaatta-ctcttaa----aacgttttgcgaatt----------gaaagttctgcc 81
cow/1-94           37 -ga---a-ttaccaa----cacaacttatgaatc----------agcaggtctact 73
dolphin/1-95       39 -ga---a-ttatcaa----cacaactaatgaatc----------agtagctctgct 75
horse/1-95         39 -ga---c-ttaccca----cacagctgatgaatc----------agtagctctgtt 75
dog/1-75           19 -aa---a-tacccaa----cacagtgtagaaatc----------agtagctctgct 55
microbat/1-105     40 -ga---a-ataccag----cacagtttatgagtcattacctctgtcagctctgct 85
megabat/1-97       41 -ga---a-ttaccgg----tacagcttatgaatc----------gttagctatgct 77
hedgehog/1-83      33 -gaatta-ccatcta----cagaatctatgtatc----------a---------gt 63
shrew/1-90         39 -a----a-tcatcaa----cacagtttatgaatc----------acttac----ct 70
rock-hyrax/1-99    38 -gagttaaccattag----cagagtgtgtaaata----------gctgagttcacc 78
elephant/1-103     39 -gaattcaccatcataaaccagagtttatgaatc----------agtaattctgcc 83
sloth/1-99         40 -gaatta-ctgtcaa----caccgtctatcaatc----------agttgccccacc 79
tenrec/1-98        39 -gaattc-ccaccaa----cacagtttaggaatc----------agtaactccgcc 78

human/1-102        84 a-g-ctttgga-ttgtataact                                   102
marmoset/1-102     84 a-g-ctttgga-ctgtataatt                                   102
baboon/1-102       84 a-g-ctttgga-ttgtataatt                                   102
chimp/1-102        84 a-g-ctttgga-ttgtataact                                   102
gorilla/1-102      84 a-g-ctttgga-ttgtataact                                   102
orangutan/1-102    84 a-g-ctttgga-ttgtataact                                   102
bushbaby/1-101     82 a-g-cttgggatttatgtaatt                                   101
mouse-lemur/1-103  84 a-t-ctttggatttatgtaatg                                   103
treeshrew/1-92     91 a-g-------------------                                   92
guinea-pig/1-95    76 t-c-ctttgaacttcctcactt                                   95
squirrel/1-99      82 a-g-ccttggattta--taatt                                   99
cow/1-94           74 g-gttttagctttatatgatt                                    94
dolphin/1-95       76 a-g-ttttggatgtatataatt                                   95
horse/1-95         76 a-g-ctttggatttatataatt                                   95
dog/1-75           56 g-g-ctttggatttatataatt                                   75
microbat/1-105     86 a-g-ctttagatttatttaatt                                   105
megabat/1-97       78 a-g-ctttgcatttacttaatt                                   97
hedgehog/1-83      64 a-g-tttaggatttataaaatt                                   83
shrew/1-90         71 a-g-tcttgtctatatacaatt                                   90
rock-hyrax/1-99    79 agg-ctttggatttatatgtag                                   99
elephant/1-103     84 a-g-ctttgaatttatatggtg                                   103
sloth/1-99         80 a-g-ctgtgggtttatttaatt                                   99
tenrec/1-98        79 a-g-ctttgaattgacgtgctg                                   98
```

* Orientation: reverse complimentary

g

Plasmid: pGL4.23-WIPI1promotor
WIPI1 promotor sequence with restriction sites

```
EMBOSS
1567 bp DNA

5'NheI highlighted in Red
3'HindIII highlighted in Magenta
M19 highlighted in Blue
M11 highlighted in Green
M+1 highlighted in Yellow

   1 GCTAGCCCAA AGCTGGCAGA GCTCCTGCTT CATAAACCGT ATTGACAGTA ATTCCGCATG
  61 GGAACATGTA CAGTACTCAG CTTTTAAAAA GAGATTCTTT GTTAAGGTTT ATCTCGAATT
 121 ACGTGAGTTT TGAATTGTGC TATATCTTCA AGACGTTACC CCTTTCATAA ACTACACTGC
 181 CCTACACACA CTTTCATTTT TTTCAGCTGC GGATCGGATA GGTCTTGTTT AGATACTTTG
 241 TGCCATTTTT GTAACTTTTC AGATCAAAGG TGCTTCCCCC CACCTTCCAG CTCCGTCAAA
 301 TGCTGCAGTC ATTTCTGTCC TGGTTAGAAA ACCTAGAATC TCGCATGCTA GGTGCTACGT
 361 TGTTTTTGCA AATTACTGTT TAGCAGTTAT TTAGTGCACA GGTCTTGAAA GCAGACATGC
 421 AGACATTCTA CCATTTACTG TTACTTTAGA CAAATTGTTT AGCTTTGGAG CTTCAGTTGC
 481 CTCTTCTGTA TATTGAGCAC AATCGTATTT TCCACACAAG TTTTATAAGA ATTCAAGATG
 541 ACATACATAG TACGGCGCGC TTGGCAAGTA GTGAGGGTTC TCAACCGCTC GGCAATGGAT
 601 AAGATAACTT GCTATCTCTC TGGCTGAGGC AGCCCCATTT CAGGAGCTTC TCTCAATGCG
 661 GCTTGAGGAC TCGACAAAAG GGTGGGGCTT TGGAGTCTAA CGCTCTCTGC TAAACTCTGT
 721 AAGTTTAATA GGCAGAGTTA CACATGGCAA GGGGTCTGGG CGGGGTGGGA ACTAGCGCGT
 781 CCTCCTGTCA CCCTTGCGGC GGCCCCGACG CAGTTGCCGG ACTGAAACCA GCAGATCTGG
 841 CCCCAGCCGC ATTCCTTCTT CCCTCTCCCT CCCAGCTGGG CGGCTCCGGC AATGAAAACA
 901 CCGCCACGTG GGCCTGCTGC CTTTGCGTCA CGCGCCTTTG TCCAATGGAG AGCCGCGTTA
 961 CGAAGCCAGG GGCGGGTCGC CAGCCGGGCG GGTCCGCCCA CAGCTGCCAC GGATTGGCCA
1021 AGGGGGGCGG TGCCCGCCAG CCGTGGCTTG GGCTCGTGGC CAATGGTGGC CGGAGGCAGG
1081 GGCCCCGCCC TATCAGGTGC TCTGGTCCTC GGGACCAGCT GGAGGGGCGA GAAGGGGCGG
1141 GGCGAAGCCG GGGACGCGGC GGGGACGGGG CGGGGTAGGG GCGGGGTGGG GACGGGGCGG
1201 GGTAGGGGCG GGGTGGGGAC GGGGCGGGGT AGGGCGGGGC AGGACCGGCG ACCCGGCCCG
1261 TGGAGCCGGC GCGGGCGGGC TGCTGAGGTG GCTGTCGCCG GCTCCGAGCT GCGGCTTCCC
1321 GGGCCGAGCC CCCGATGGAG GCCGAGGCCG CGGACGCTCC CCCGGGCGGG GTTGAGTCGG
1381 CGCTCAGCTG CTTCTCTTTC AACCAGGACT GCACGTAAGC TGCGACTCGG CCCCTGCCAG
1441 GGAGGTGGGG GACAGAGTGT CAGGACCCAG GGGTGTCGTC CTGAGAGCAG CTTGTGGCTT
1501 CCGCCTCGAG GCGGCCTCGG CGGGAGGGCC GGTGTCTGCT TCGGACGCAT TGGGATCTTG
1561 TAAGCTT
```

h

Plasmid: pGL4.23-5xE-box
5x canonical E-box sequence with restriction sites

```
5'XhoI highlighted in Yellow
3'HindIII highlighted in Magenta
Canonical E-box highlighted in Cyan

EMBOSS
312 bp DNA

   1 CTCGAGAGTT CCCACCCCGC CCAGACCCCT TGCCACGTGC AACTCTGCCT ATTAAACTTA
  61 CAGAGTAGTT CCCACCCCGC CCAGACCCCT TGCCACGTGC AACTCTGCCT ATTAAACTTA
 121 CAGAGTAGTT CCCACCCCGC CCAGACCCCT TGCCACGTGC AACTCTGCCT ATTAAACTTA
 181 CAGAGTAGTT CCCACCCCGC CCAGACCCCT TGCCACGTGC AACTCTGCCT ATTAAACTTA
 241 CAGAGTAGTT CCCACCCCGC CCAGACCCCT TGCCACGTGC AACTCTGCCT ATTAAACTTA
 301 CAGAGTAAGC TT
```

i

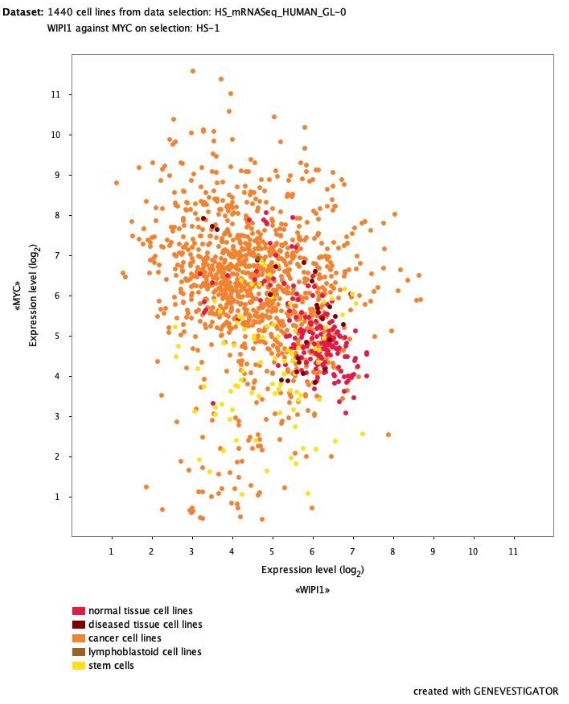

Supplementary Fig. 5: Human WIPI1 promoter analysis. (**a**) Volcano plot of gene expression array analysis. RNA was extracted from U2OS cells after downregulation of c-MYC (siMYC) or nontarget control (siControl) for 48 h (control TaqMan qPCR displayed in the left panels, Welch's t test, mean±SD, n=3), and ATG and ATG-related gene expression (84 autophagy genes) was profiled by qRT–PCR. Log(2) (fold change siDDR1 over siControl) against log(10) (p value) is shown in the middle panel. Fold changes of mRNA >1 and p values <0,05 were considered differentially up- or downregulated (n=3 in technical duplicates). The WIPI1 data point is circled in black. ATG genes upregulated upon c-MYC knockdown are visualised using the Search Tool for the Retrieval of Interacting Genes/Proteins (STRING) database (right panel). (**b**) Validation of WIPI1 upregulation by subsequent TaqMan qPCR. Relative mRNA levels of siControl and siMYC are shown, also including WIPI2, WIPI3 and WIPI4 probes as indicated. Welch's t test, mean±SD, n=3 in triplicates. (**c**) Relative mRNA levels of WIPI1 in U2OS subjected to ABL1/2 downregulation and c-MYC overexpression. U2OS cells were transfected with siRNAs targeting ABL1/2 (siABL1/2) or nontargeting control siRNAs (siControl) for 16h. Cells were subsequently transfected with a plasmid overexpressing c-MYC (pCMV3-c-MYC) or the empty pCMV3 plasmid in the presence or absence of AZD0364 administration for 24h as indicated. RNA was extracted and the expression level of WIPI1 was assessed by TaqMan qPCR (Welch's t test, mean±SD, n=3 in triplicates). **(d)** Primer pairs and human WIPI1 promoter areas analysed by ChIP in **Fig. 3f-h** (upper panels) are displayed. (**e, f**) E-boxes (M11, M19) predicted using ConTra v3 are displayed as multiple nucleotide sequence alignments derived from different species. **(g)** The WIPI1 promotor sequence cloned into pGL4.23[luc2/minP] to generate is pGL4.23-WIPI1promotor for luciferase assays is displayed. (**h**) The sequence habouring 5 canonical E-boxes cloned into pGL4.23[luc2/minP] to generate pGL4.23-5xE-box is shown. (**i**) Genevestigator-derived correlation meta-analysis of WIPI1/MYC gene expression. Displayed are the expression levels (mRNASeq) of MYC (log(2)) and WIPI1 (log(2)) as a 2-Gene-Plot, considering 1440 human cell lines, including normal tissue cell lines (red) and cancer cell lines (orange). Supplementary material is available (**Supplementary Data 1**). P values: * p<0.05; ** p<0.01; *** p<0.001; ns=not significant.

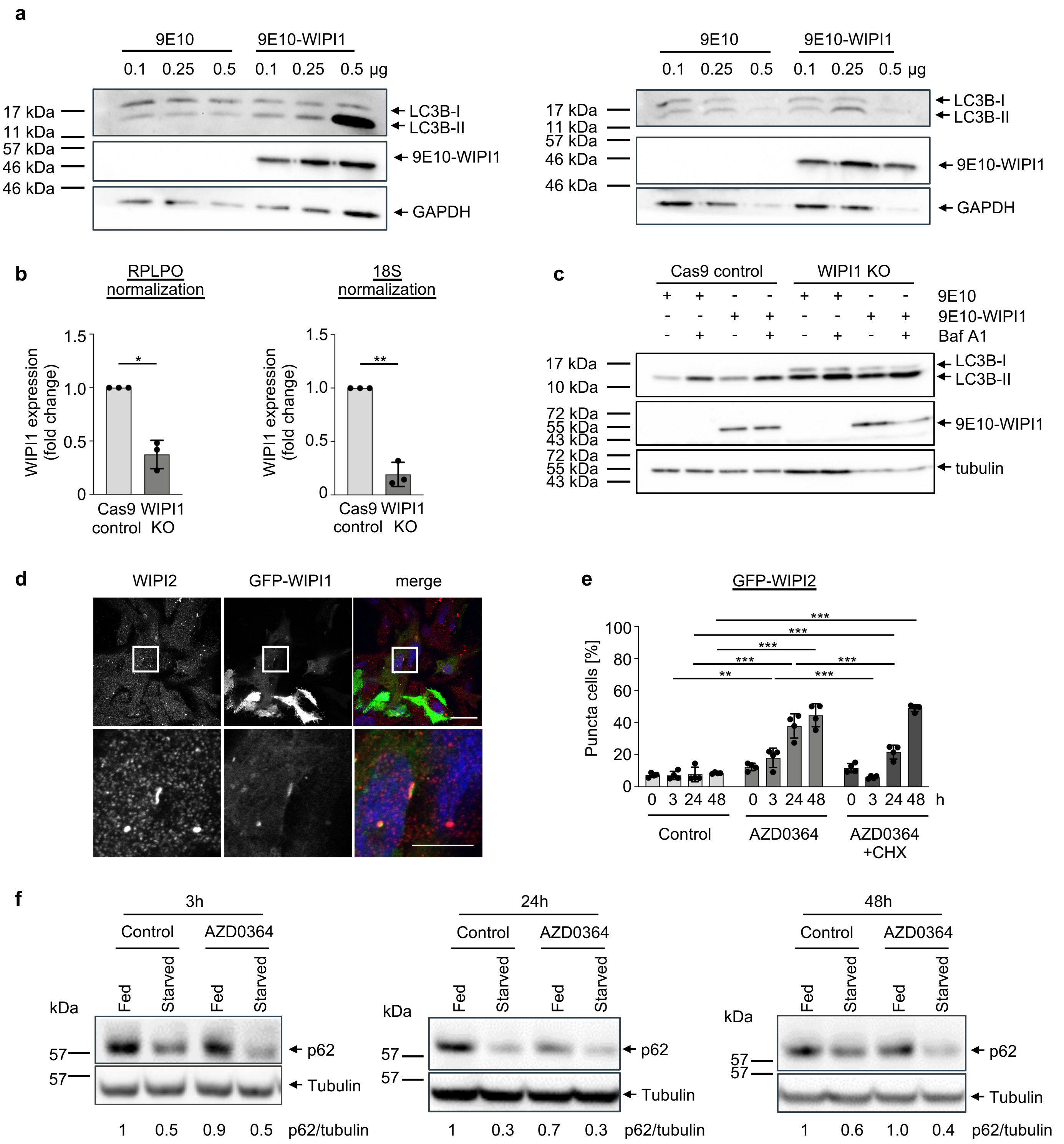
a
9E10
9E10-WIPI1
0.1 0.25 0.5 0.1 0.25 0.5 µg
17 kDa
11 kDa
57 kDa
46 kDa
46 kDa
LC3B-I
LC3B-II
9E10-WIPI1
GAPDH
b
RPLPO normalization
18S normalization
WIPI1 expression (fold change)
Cas9 control
WIPI1 KO
c
Cas9 control
WIPI1 KO
9E10
9E10-WIPI1
Baf A1
10 kDa
72 kDa
55 kDa
43 kDa
tubulin
d
WIPI2
GFP-WIPI1
merge
e
GFP-WIPI2
Puncta cells [%]
0 3 24 48 h
Control
AZD0364
AZD0364 +CHX
f
3h
24h
48h
Control
AZD0364
Fed
Starved
kDa
p62
Tubulin
1 0.5 0.9 0.5 p62/tubulin
1 0.3 0.7 0.3 p62/tubulin
1 0.6 1.0 0.4 p62/tubulin

Supplementary Fig. 6: WIPI1 enhances the autophagic flux. (a) Additional immunoblots regarding the results presented in Fig. 4a are presented. Scale bar: 20 µm. (**b**) Control Taqman qPCR of U2OS Cas9 control and WIPI1 KO cells (Welch's t test, mean±SD, n=3). (**c)** Representative Western blot for results shown in **Fig. 4b**. (d) Extended image display regarding the image section shown in Fig. 4e (right panel). (**e**) WIPI2 puncta formation was assessed in U2OS cells stably expressing GFP-WIPI2 after treatment with the ERK2 inhibitor AZD0364 with or without cycloheximide (CHX) for the indicated times. The numbers of GFP-WIPI2 puncta cells were counted. Two-way ANOVA with Tukey's post hoc test, mean±SD, up to 1366 analysed cells from n=4 for each condition. (**f**) U2OS cells were treated with AZD0364 (or not) for the indicated times, fed or starved for 3 h and subjected to immunoblotting against p62 or tubulin (p62/tubulin ratios are displayed). Supplementary material is available (**Supplementary Data 1**). P values: * $p<0.05$; ** $p<0.01$; *** $p<0.001$; ns=not significant.

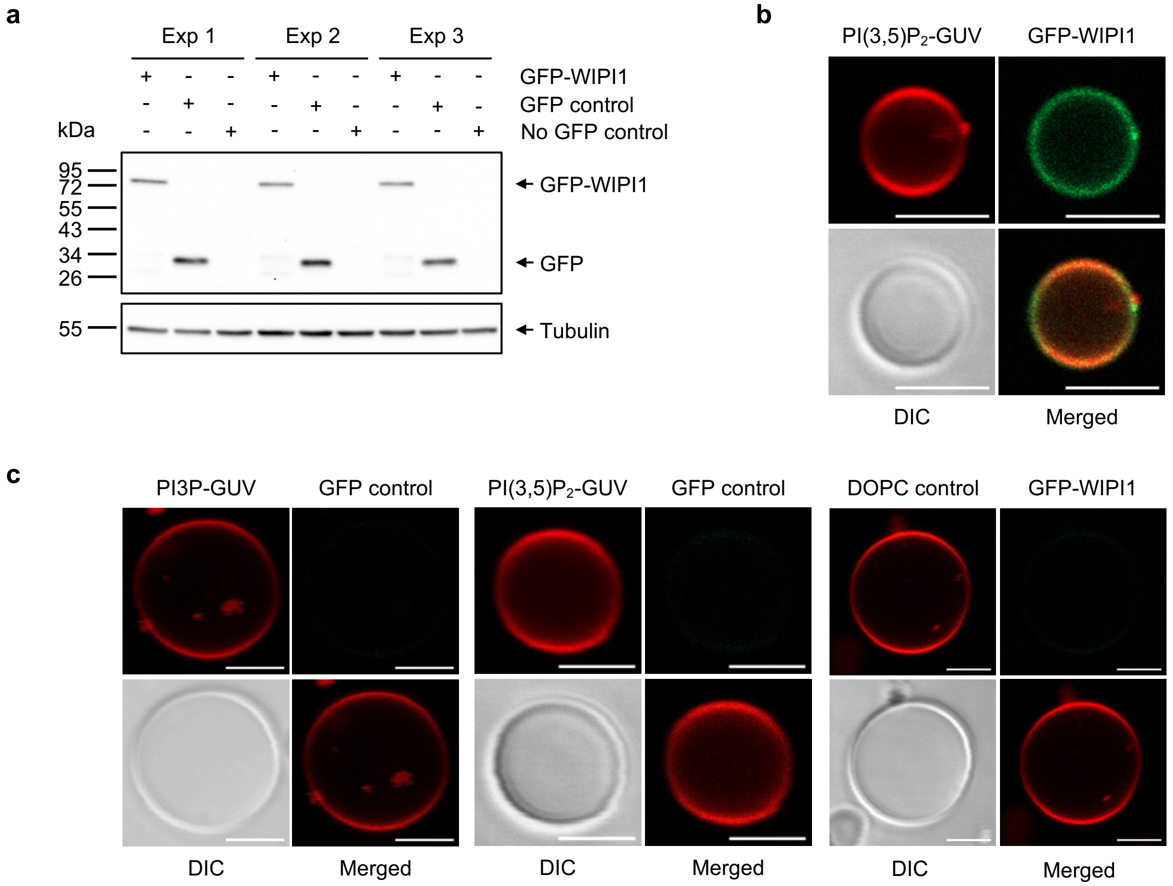
a
Exp 1
Exp 2
Exp 3
GFP-WIPI1
GFP control
No GFP control
kDa
95
72
55
43
34
26
55
GFP-WIPI1
GFP
Tubulin
b
PI(3,5)P2-GUV
GFP-WIPI1
DIC
Merged
c
PI3P-GUV
GFP control
PI(3,5)P2-GUV
GFP control
DOPC control
GFP-WIPI1
DIC
Merged
DIC
Merged
DIC
Merged

Supplementary Fig. 7: Control experiments regarding the results presented in Fig. 5e. (**a**) Aliquots of native cell extracts prepared for GUV experiments were separated by SDS–PAGE followed by anti-GFP ECL (n=3, independent experiments indicated by Exp 1 through Exp 3). (**b**) GUVs containing PI(3,5)P_2 were incubated with native protein extracts from U2OS GFP-WIPI1 (left) or U2OS GFP control cells (right) as indicated. Scale bar: 5 µm (**c**) Control incubations of PI3P-GUVs (left panels) or PI(3,5)P_2-GUVs (middle panels) with native extracts from U2OS GFP control cells are shown. GUVs lacking PI3P and PI(3,5)P_2 and incubated with native protein extracts from U2OS GFP-WIPI1 are also shown (right panels). Scale bar: 5 µm.

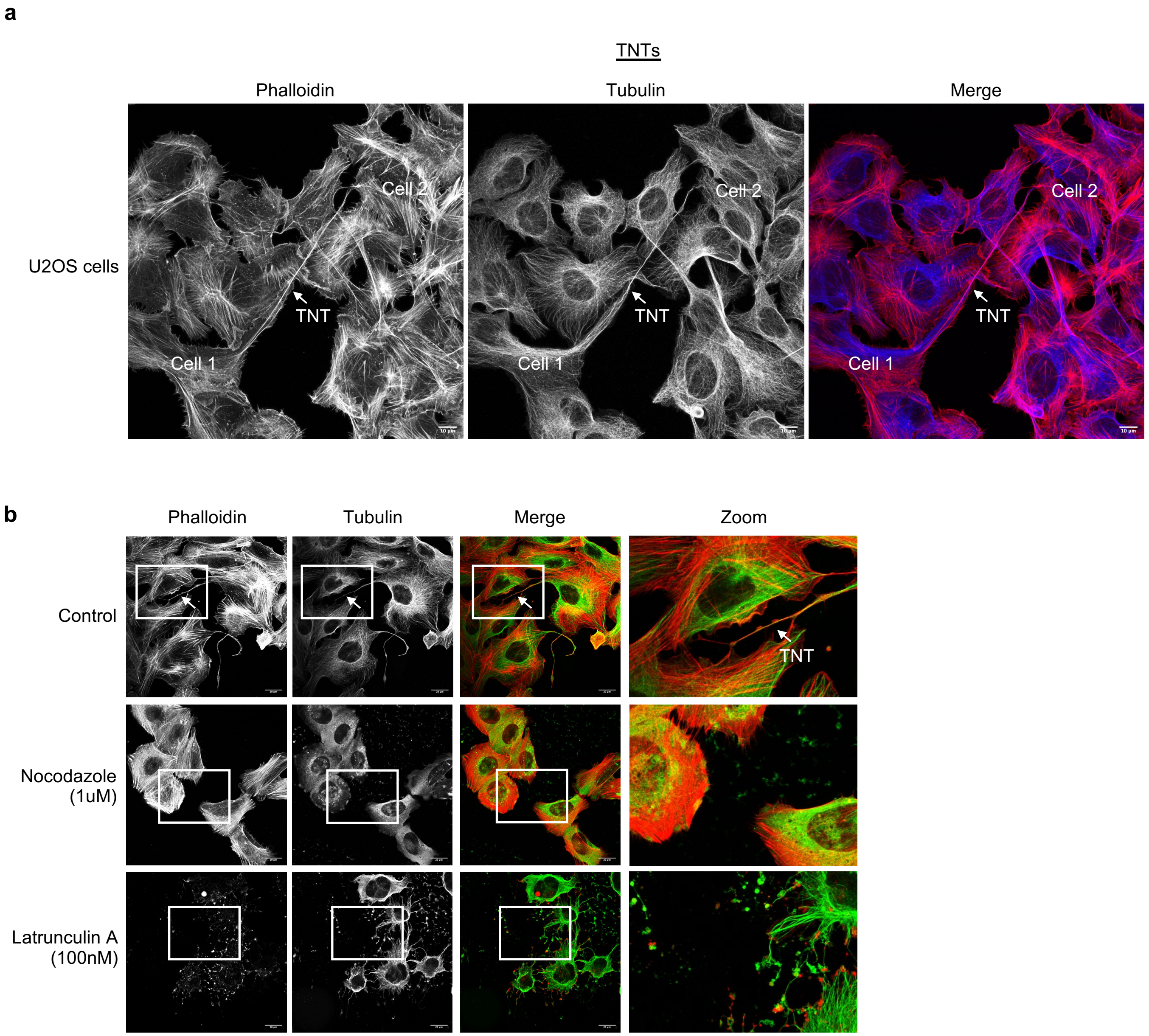

a
TNTs
Phalloidin
Tubulin
Merge
U2OS cells
Cell 1
Cell 2
TNT
b
Phalloidin
Tubulin
Merge
Zoom
Control
TNT
Nocodazole (1uM)
Latrunculin A (100nM)

c

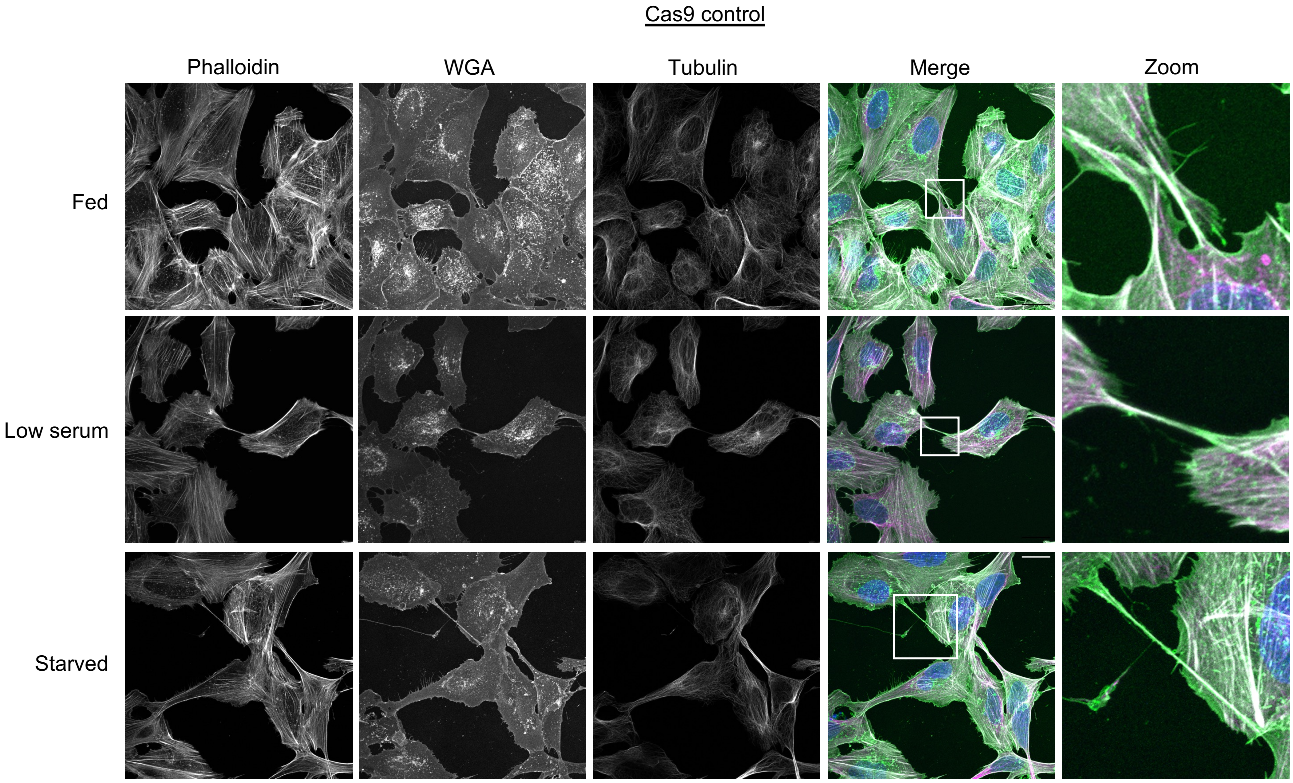

Cas9 control
Phalloidin
WGA
Tubulin
Merge
Zoom
Fed
Low serum
Starved

d

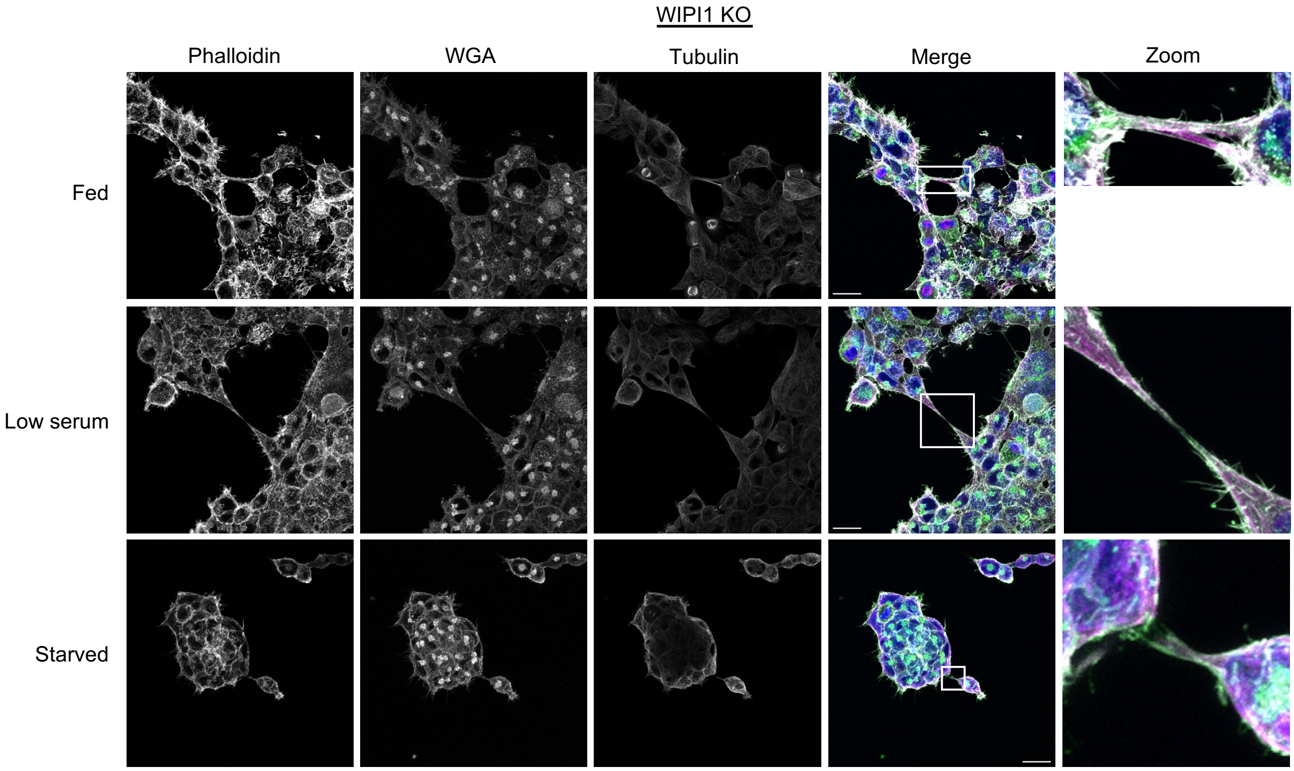

WIPI1 KO
Phalloidin
WGA
Tubulin
Merge
Zoom
Fed
Low serum
Starved

e

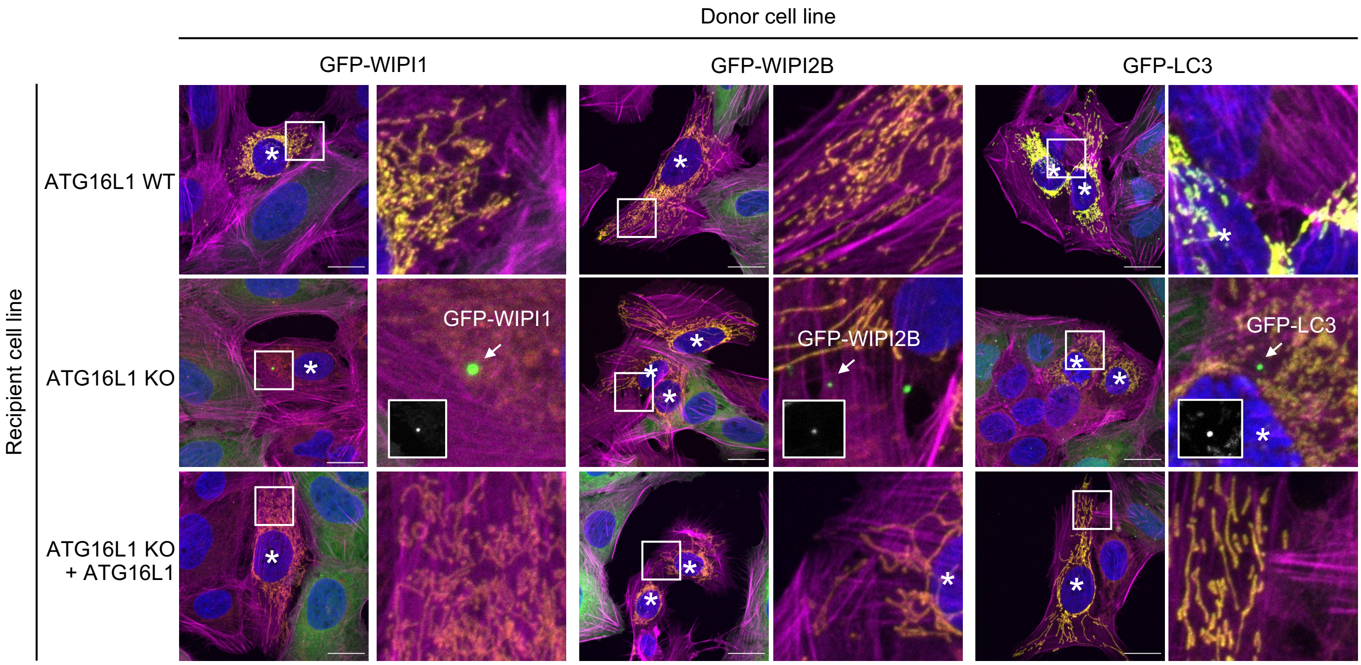
Donor cell line
GFP-WIPI1
GFP-WIPI2B
GFP-LC3
Recipient cell line
ATG16L1 WT
ATG16L1 KO
ATG16L1 KO + ATG16L1
GFP-WIPI1
GFP-WIPI2B
GFP-LC3

Supplementary Fig. 8: U2OS form TNTs and can compensate for ATG16L1 deficiency by transferring GFP-WIPI1, GFP-WIPI2B and GFP-LC3. (**a**) U2OS cells connected through TNT containing F-actin and tubulin. Cells were costained with phalloidin-AF546 for visualization of F-actin, and anti-αTubulin/AF488 allowed visualization of microtubules. Scale bars: 10 µm. (**b**) U2OS cells treated with nocodazole (1 µM) or lantrunculin A (100 nM) for 24 h were no longer able to form TNTs, as observed by costaining with phalloidin-AF546 and anti-αTubulin/AF488. Scale bars: 20 µm. (**c, d**) Representative image gallery related to **Fig. 6f**. (**e**) Extended image gallery related to **Fig. 6k**. Recipient U2OS cell lines expressing ATG16L1, ATG16L1-deficient or ATG16L1-deficient ATG16L1 in an ATG16L1-deficient background and stably expressing MLS-EGFP-mCherry under fed conditions were cocultured with donor U2OS cells stably expressing GFP-WIPI1, GFP-WIPI2B or GFP-LC3. All recipient cell lines received GFP-WIPI1, GFP-WIPI2B or GFP-LC3 puncta derived from the respective donor cell lines. Scale bars: 20 µm.

Figure 1e

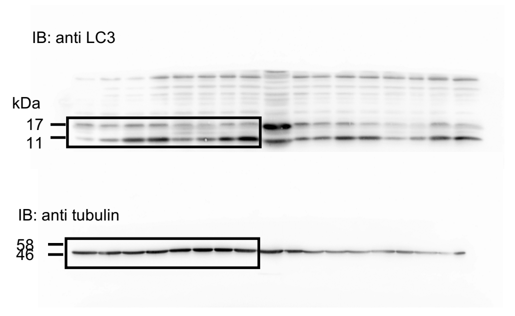

Figure 1f

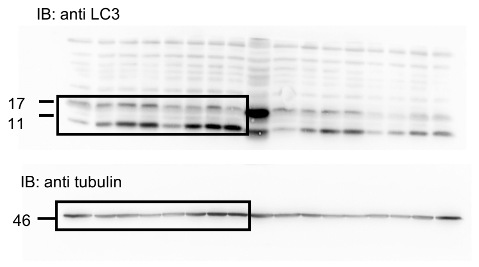

Figure 1g

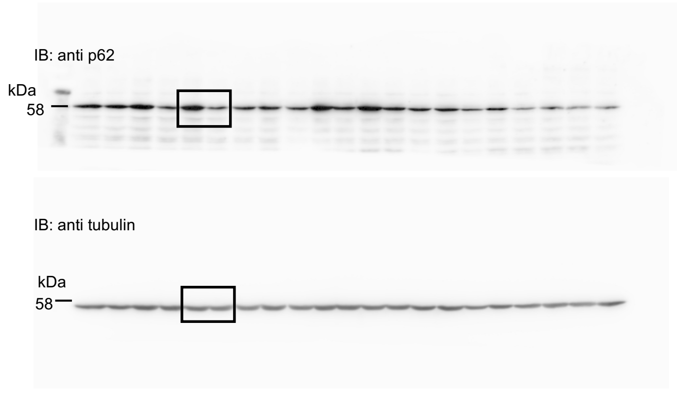

Figure 1h

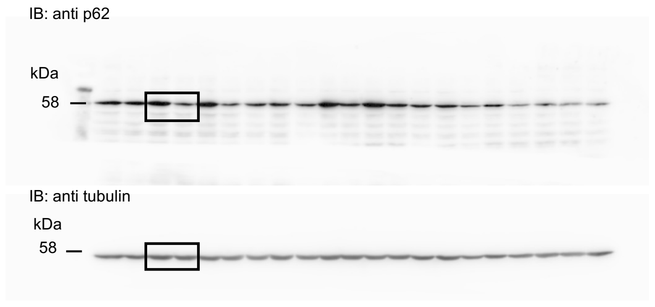

Figure 2a

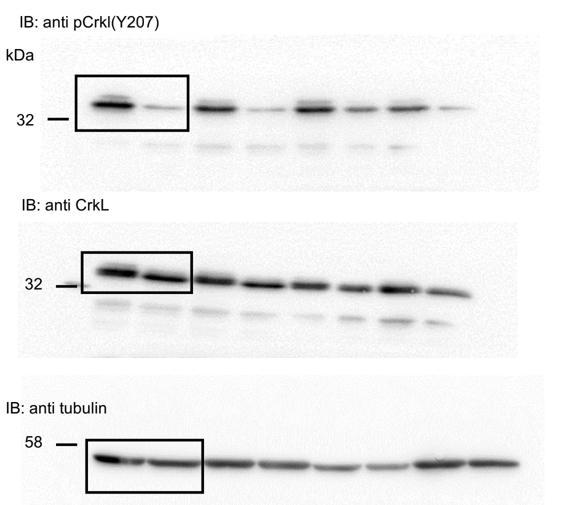

Figure 2b

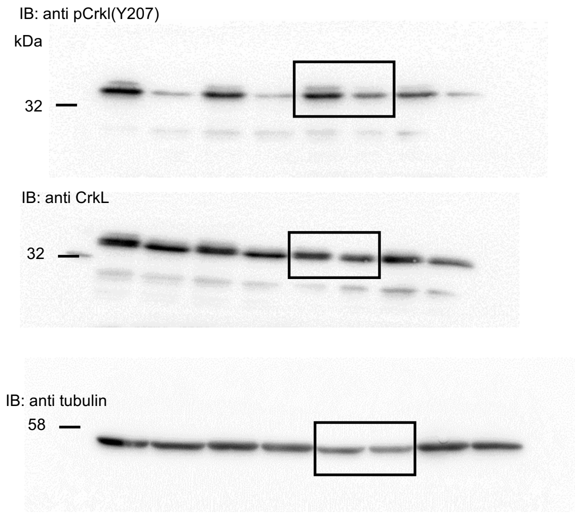

Figure 2e

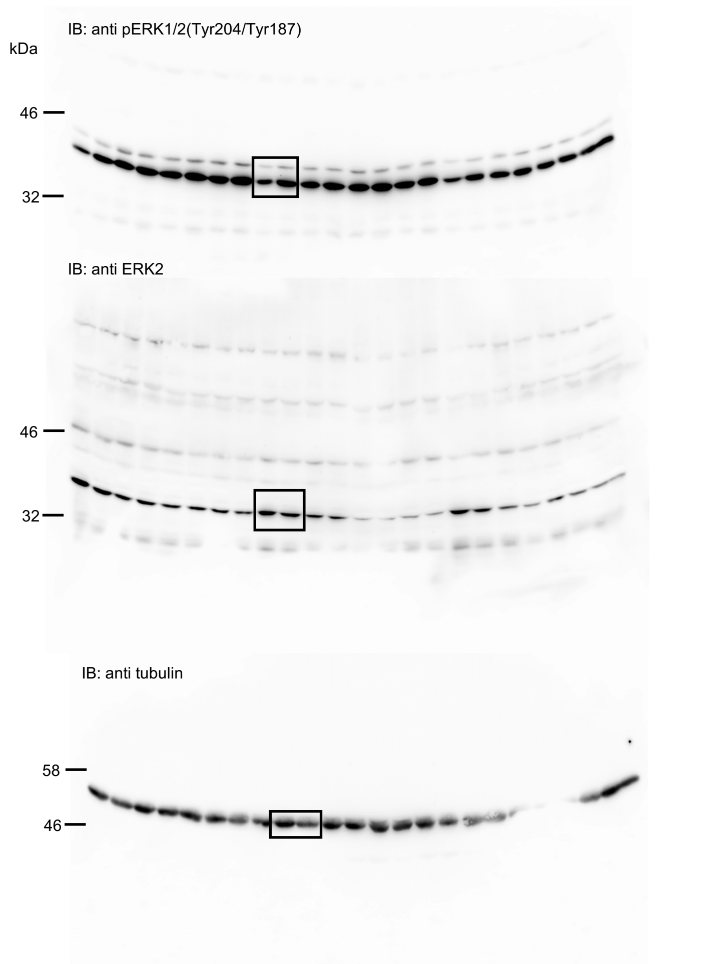

Figure 2f

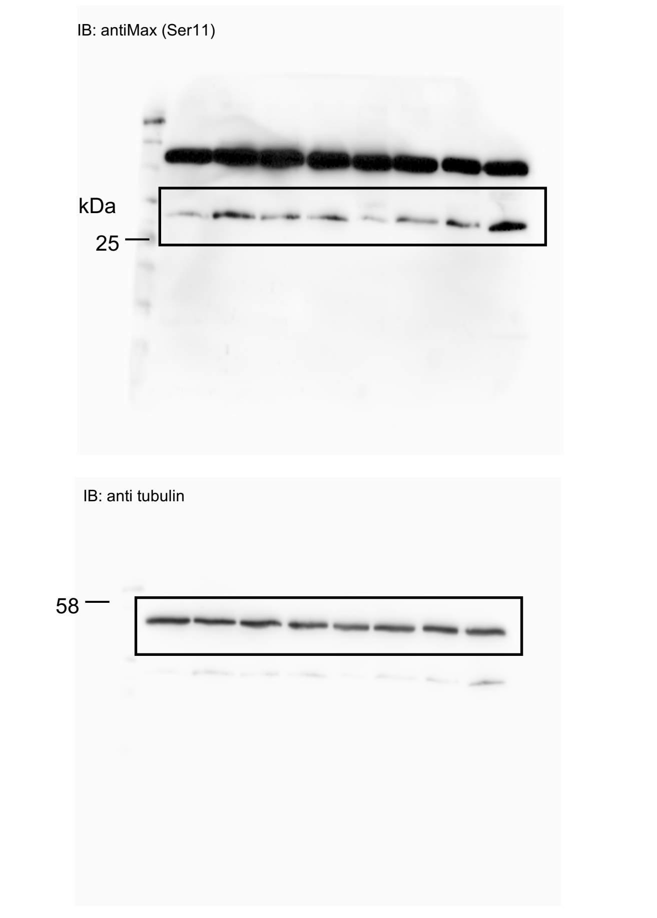

Figure 3b

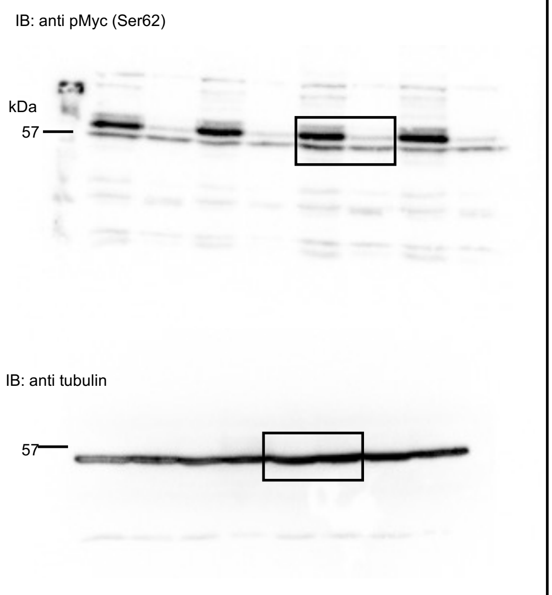

Figure 4a

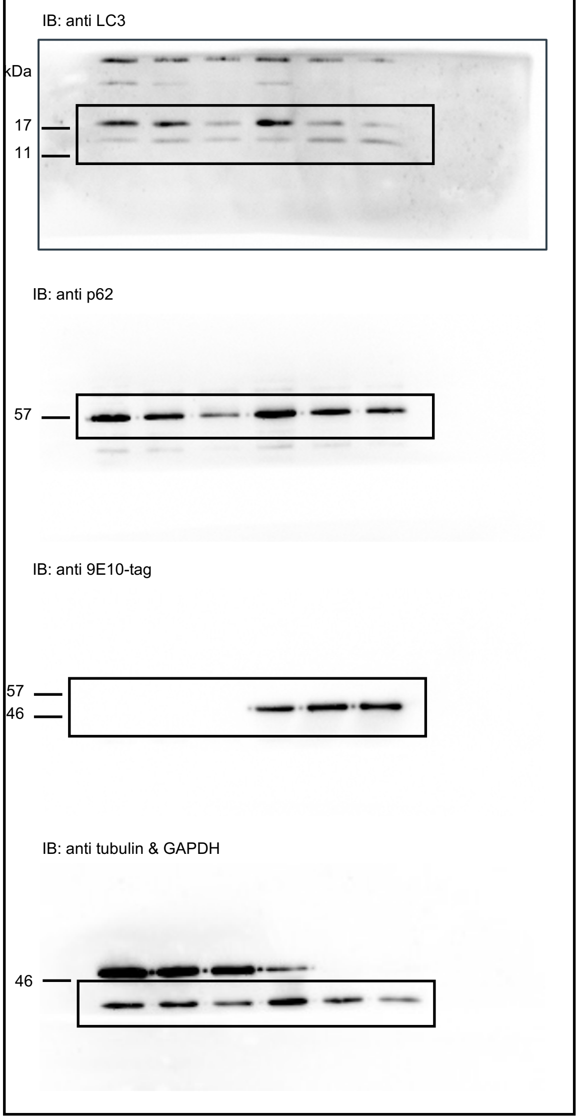
IB: anti LC3
kDa
17
11
IB: anti p62
57
IB: anti 9E10-tag
57
46
IB: anti tubulin & GAPDH
46

Figure 7c

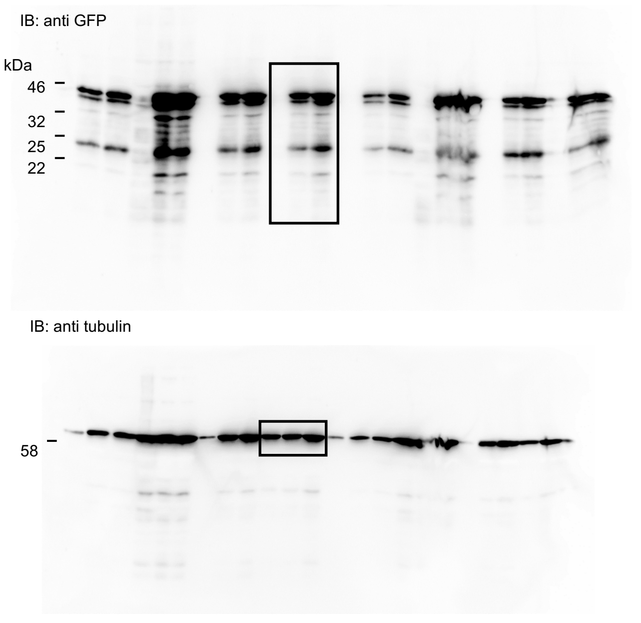

Figure S2b

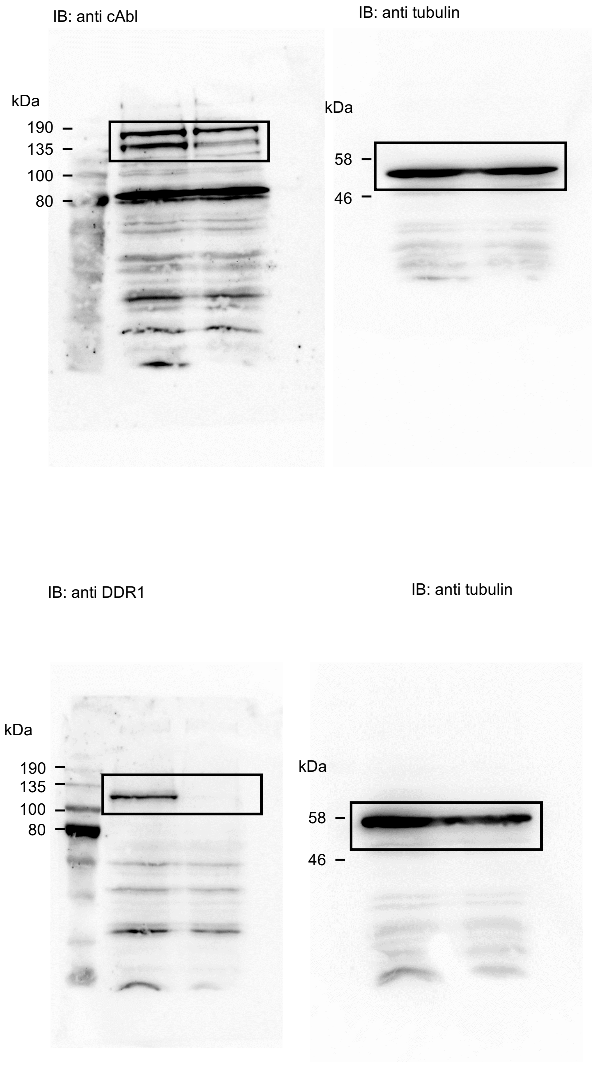
IB: anti cAbl
kDa
190
135
100
80
IB: anti tubulin
kDa
58
46
IB: anti DDR1
kDa
190
135
100
80
IB: anti tubulin
kDa
58
46

Figure S2h

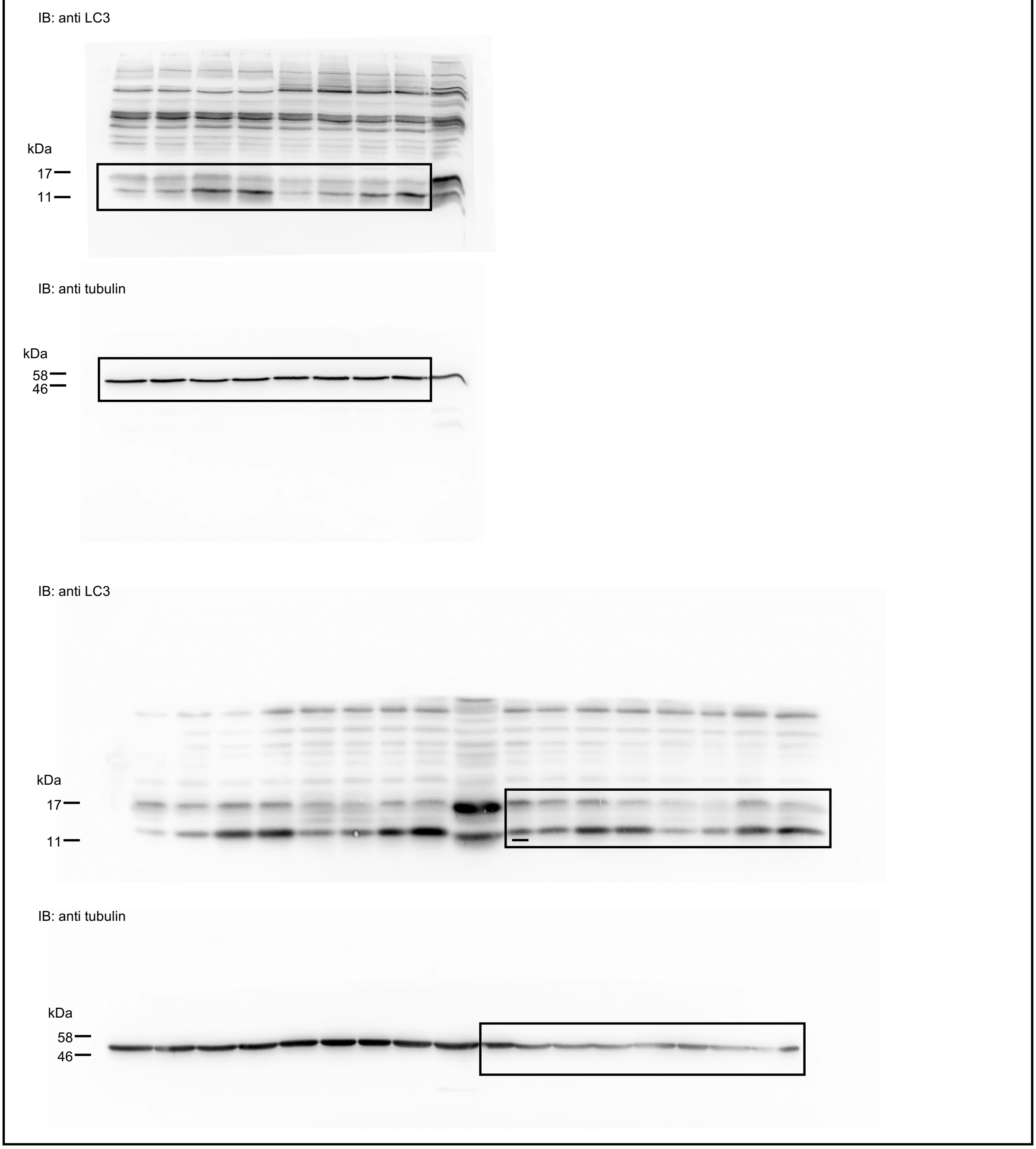

Figure 2i

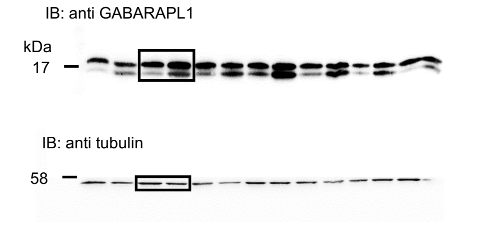

Figure S2j

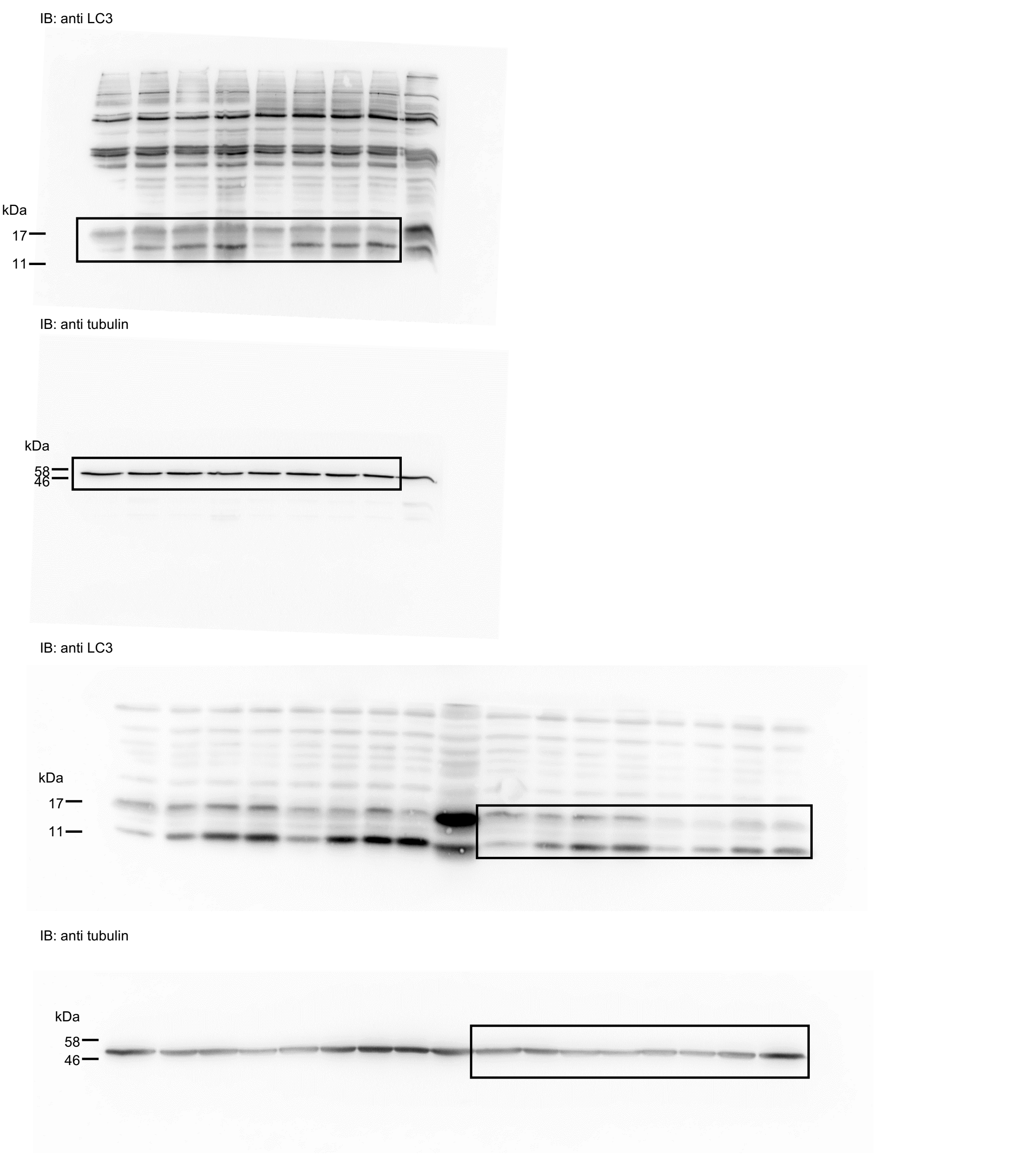

Figure S2n

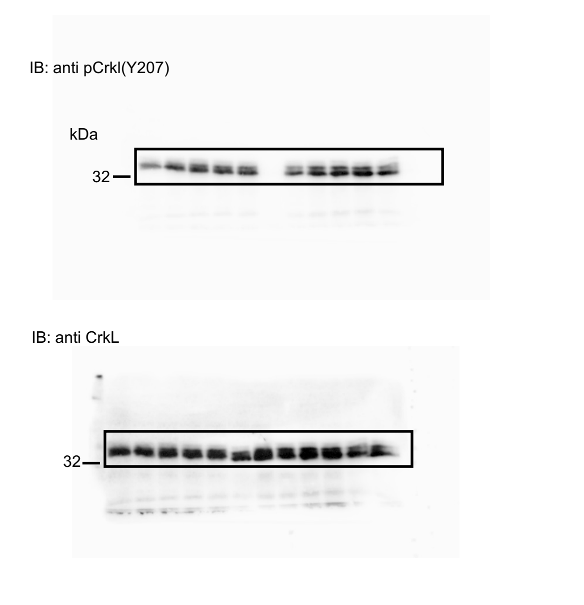

Figure S4a

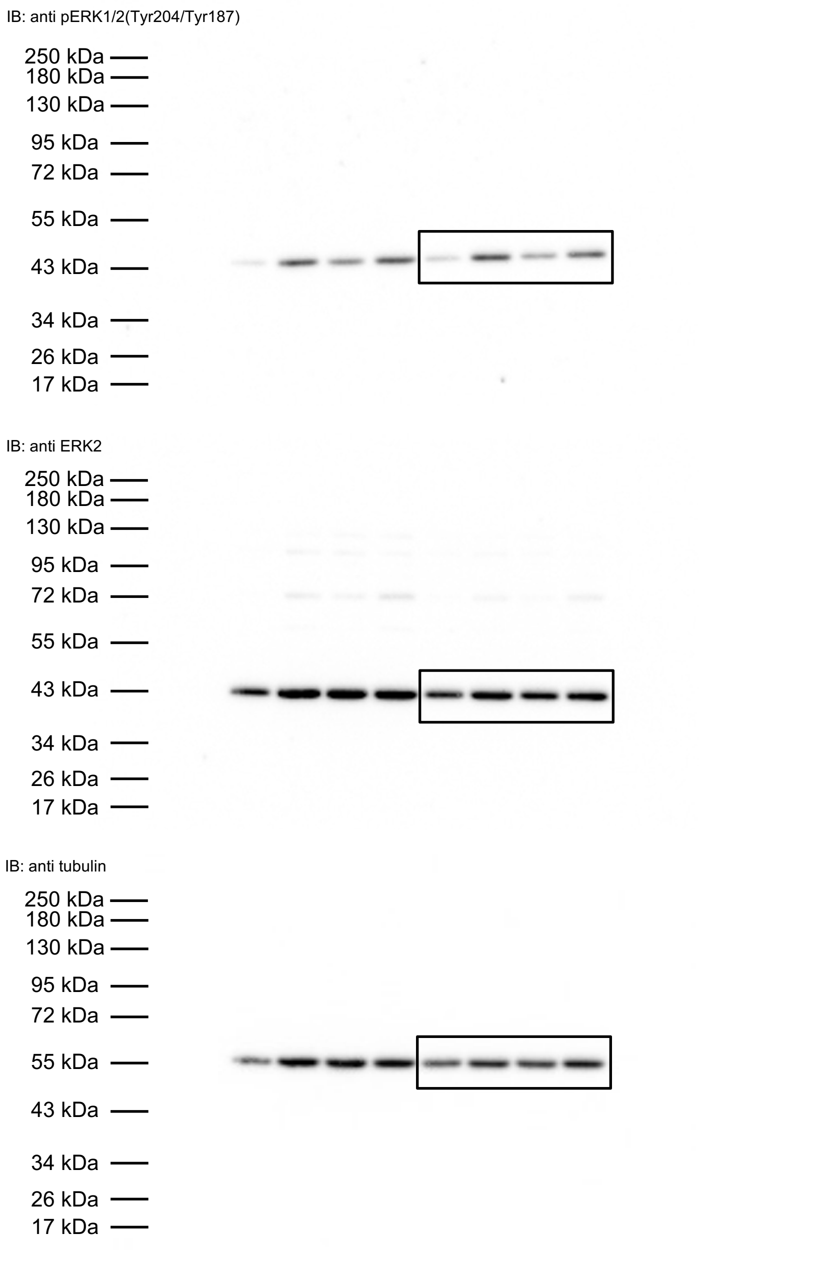
IB: anti pERK1/2(Tyr204/Tyr187)
250 kDa
180 kDa
130 kDa
95 kDa
72 kDa
55 kDa
43 kDa
34 kDa
26 kDa
17 kDa
IB: anti ERK2
250 kDa
180 kDa
130 kDa
95 kDa
72 kDa
55 kDa
43 kDa
34 kDa
26 kDa
17 kDa
IB: anti tubulin
250 kDa
180 kDa
130 kDa
95 kDa
72 kDa
55 kDa
43 kDa
34 kDa
26 kDa
17 kDa

Figure S4b

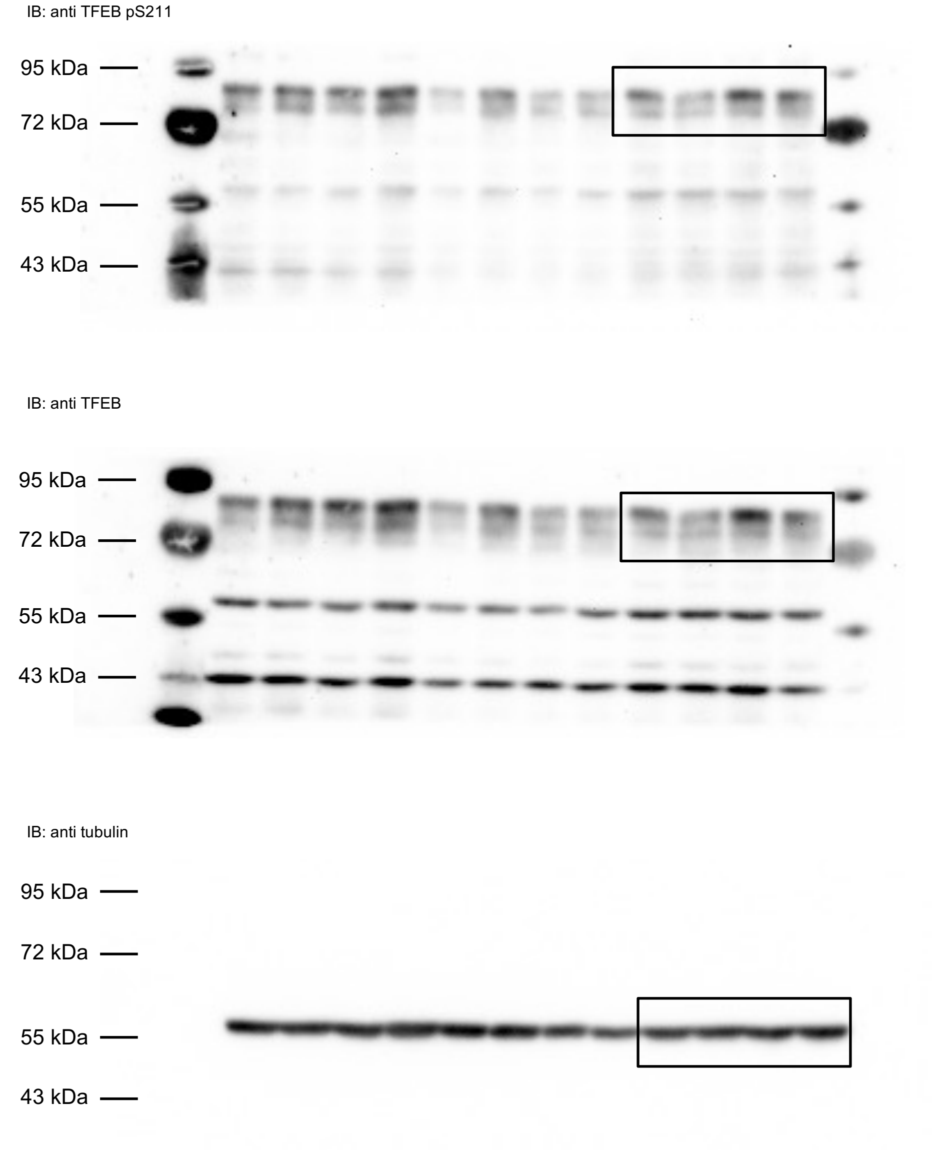
IB: anti TFEB pS211
95 kDa
72 kDa
55 kDa
43 kDa
IB: anti TFEB
95 kDa
72 kDa
55 kDa
43 kDa
IB: anti tubulin
95 kDa
72 kDa
55 kDa
43 kDa

Figure S4c upper part

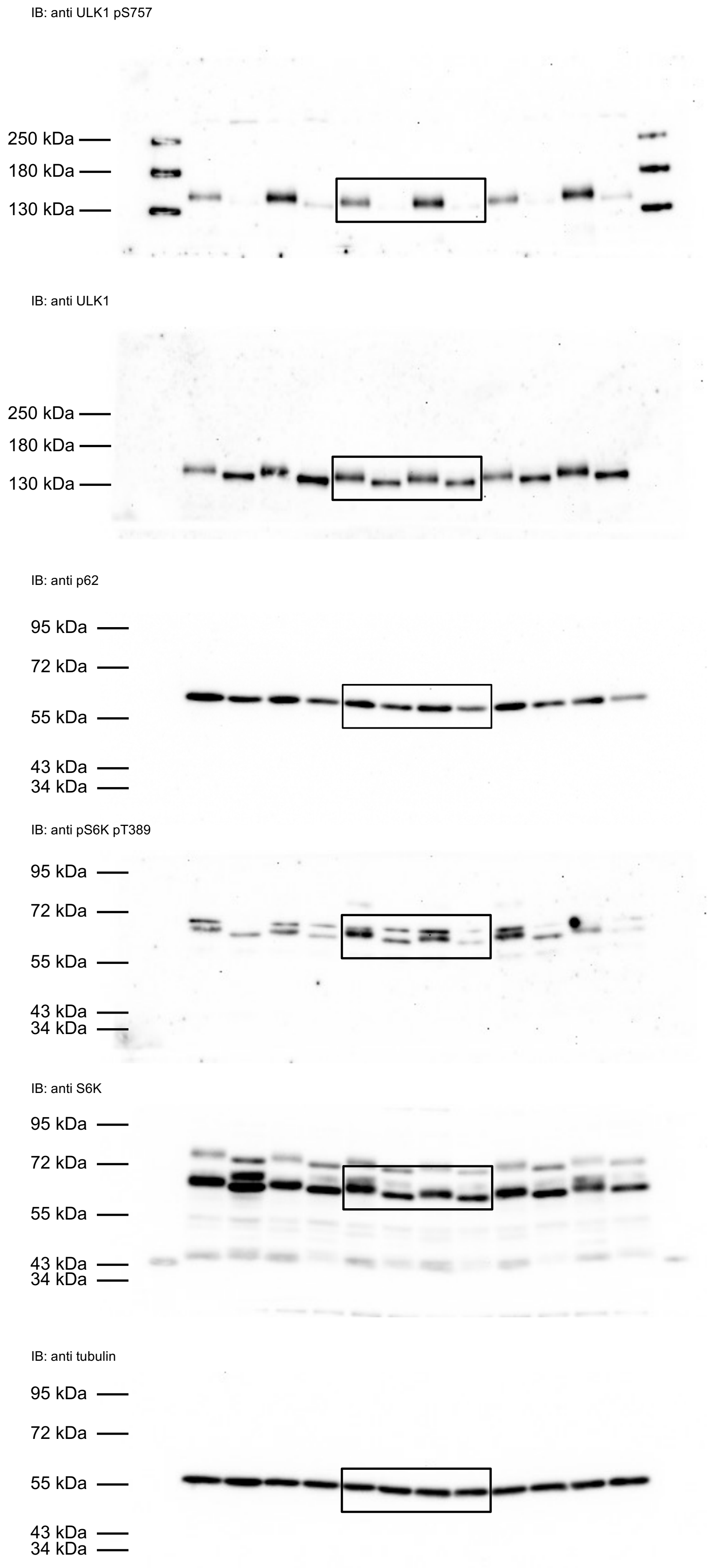

Figure S4c lower part

IB: anti ULK1 pS757

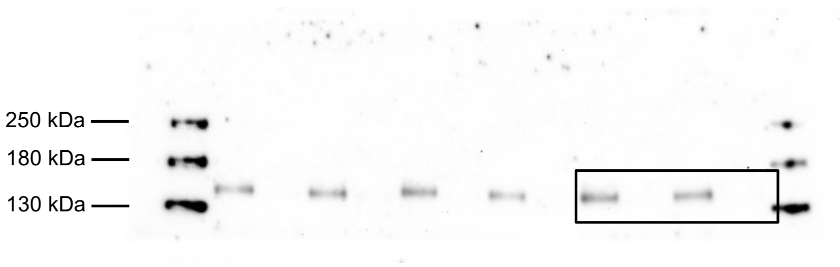

IB: anti ULK1

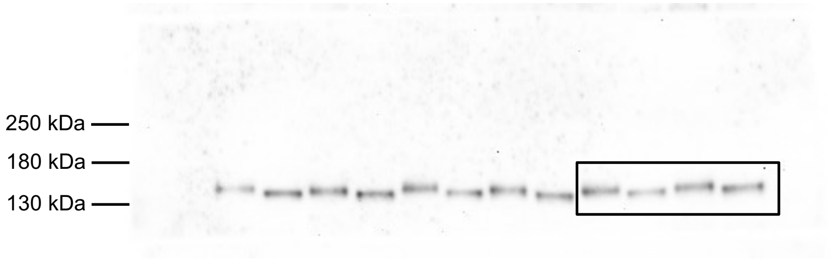

IB: anti p62

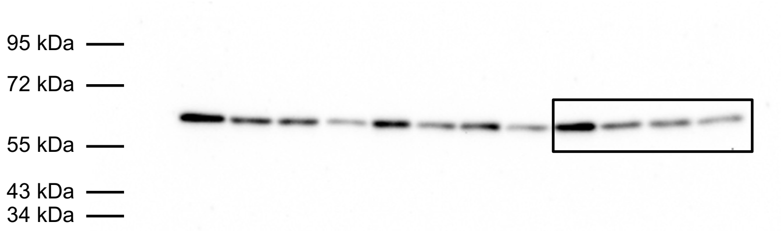

IB: anti pS6K pT389

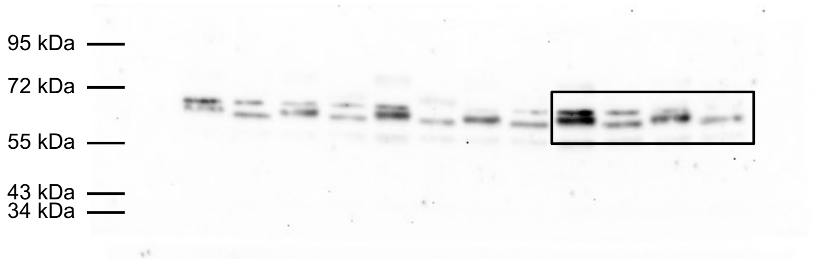

IB: anti S6K

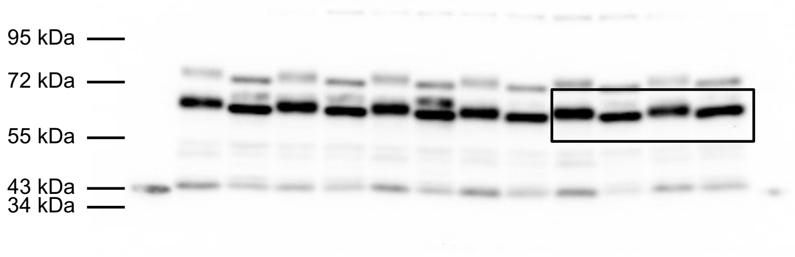

IB: anti tubulin

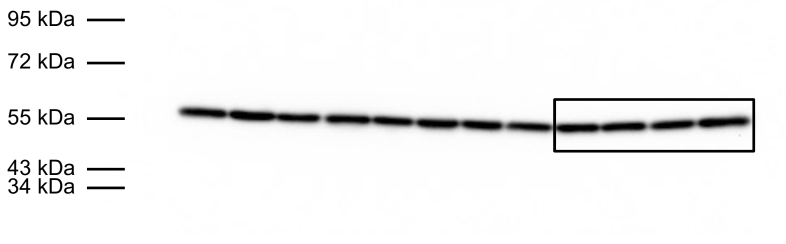

Figure S4g

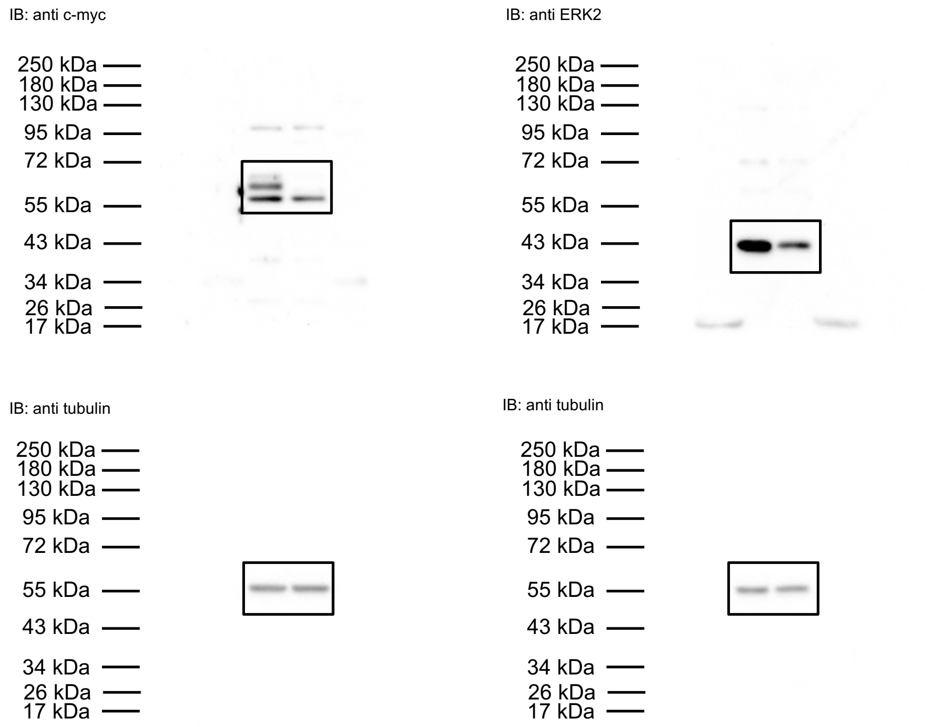

Figure S4h

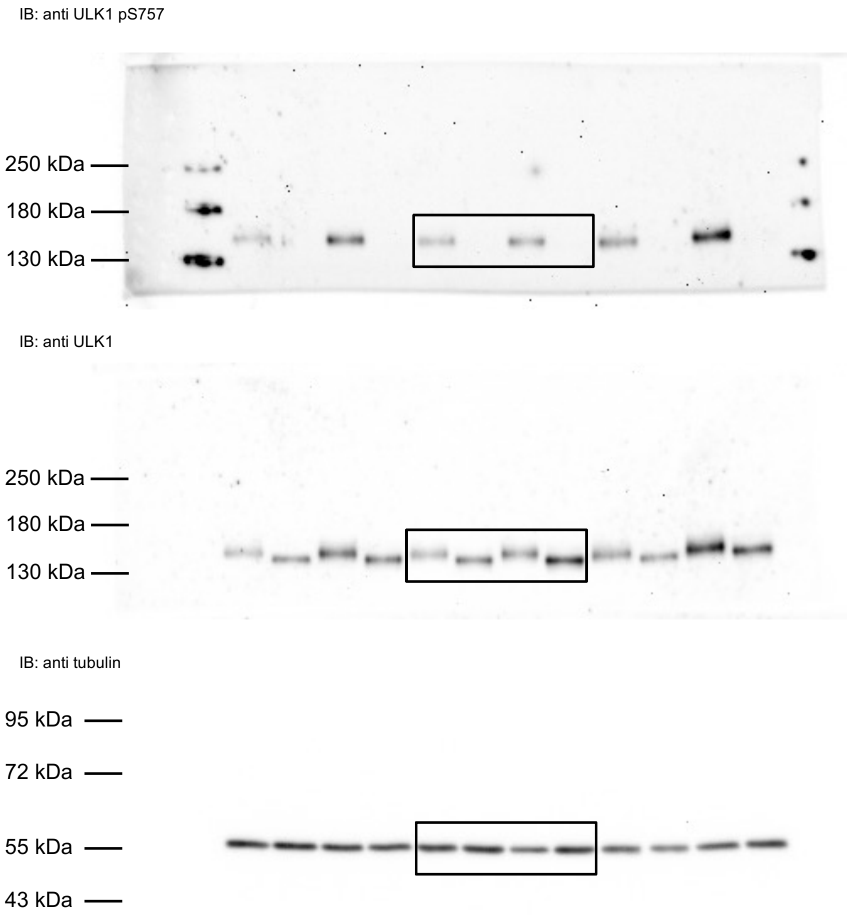

Figure S4i

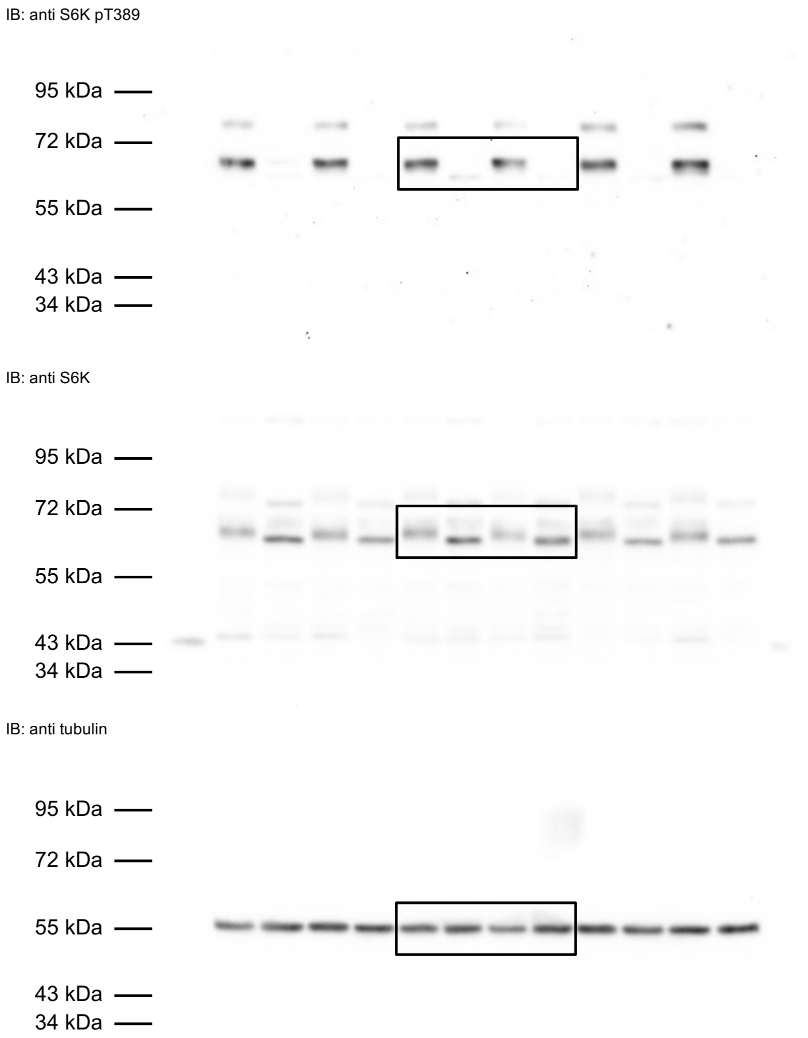

Figure S4j

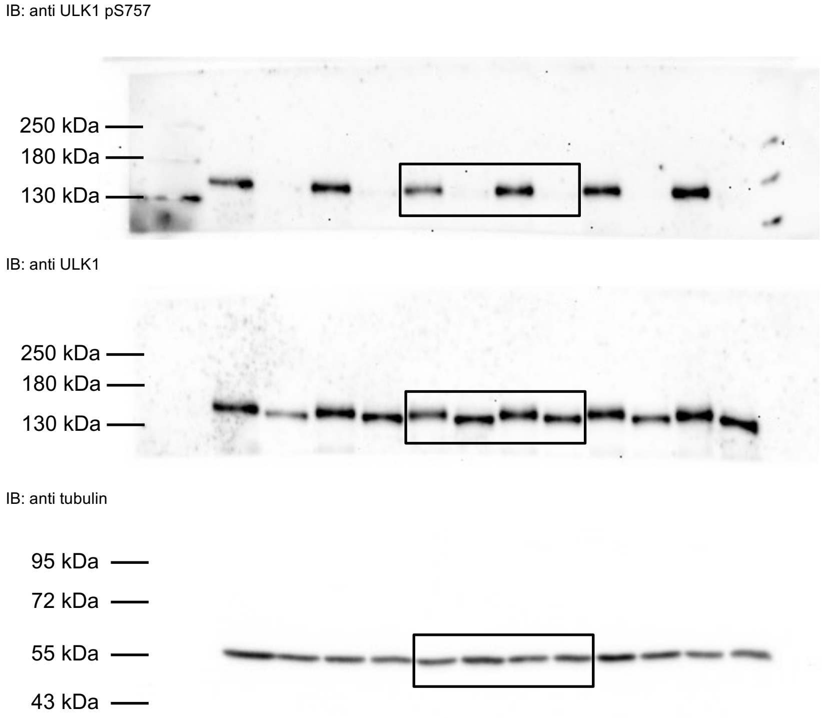

Figure S4k

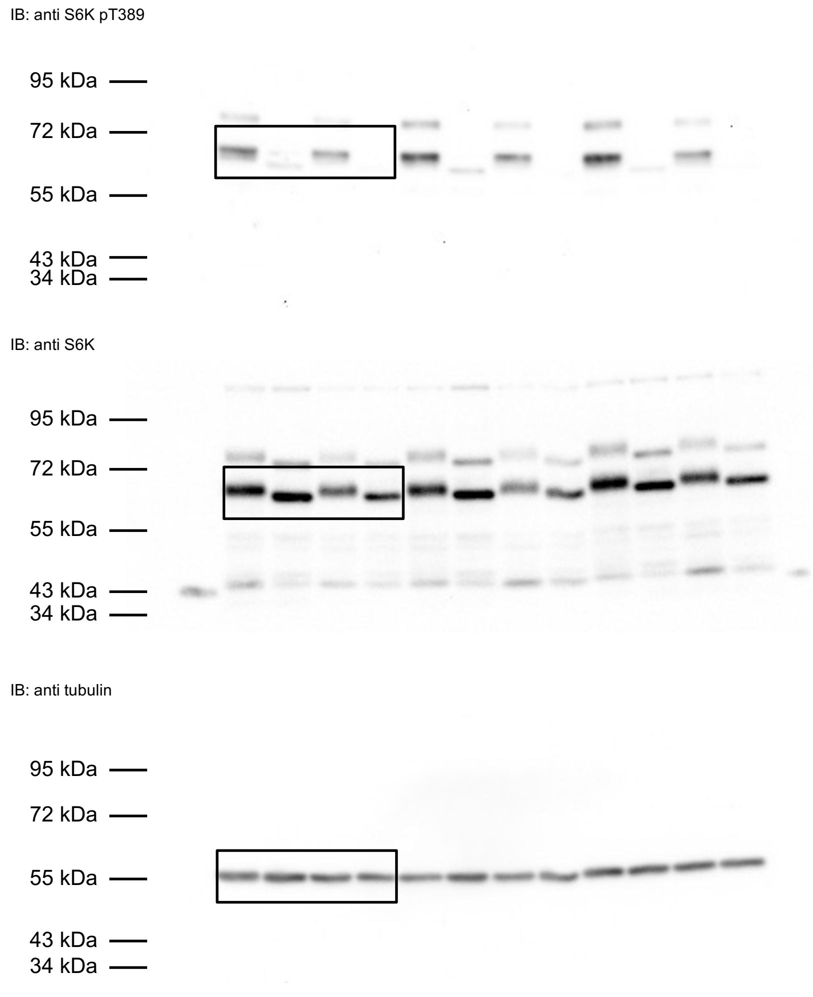
IB: anti S6K pT389
95 kDa
72 kDa
55 kDa
43 kDa
34 kDa
IB: anti S6K
95 kDa
72 kDa
55 kDa
43 kDa
34 kDa
IB: anti tubulin
95 kDa
72 kDa
55 kDa
43 kDa
34 kDa

Figure S4I

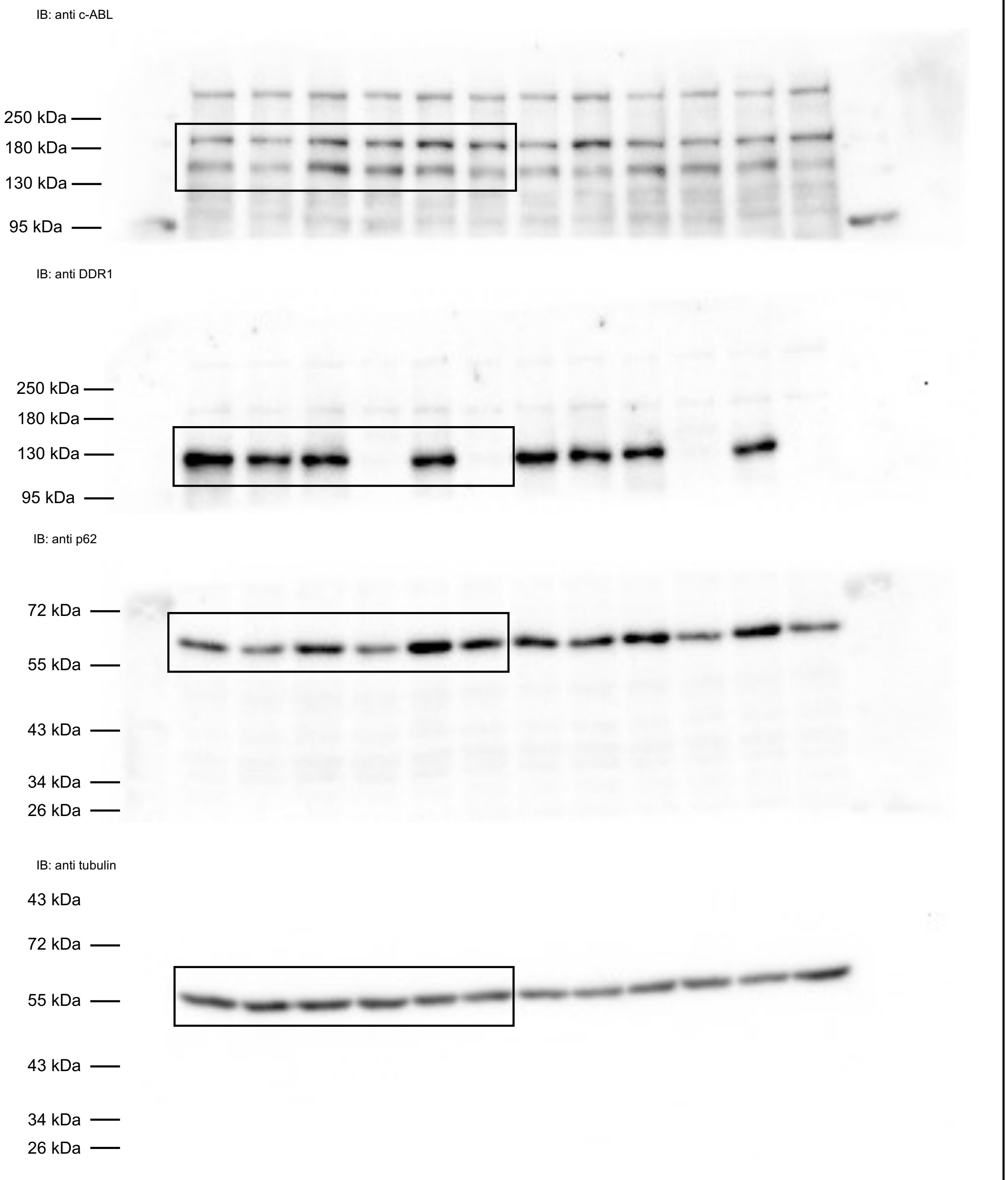
IB: anti c-ABL
250 kDa
180 kDa
130 kDa
95 kDa
IB: anti DDR1
250 kDa
180 kDa
130 kDa
95 kDa
IB: anti p62
72 kDa
55 kDa
43 kDa
34 kDa
26 kDa
IB: anti tubulin
43 kDa
72 kDa
55 kDa
43 kDa
34 kDa
26 kDa

Figure S4k

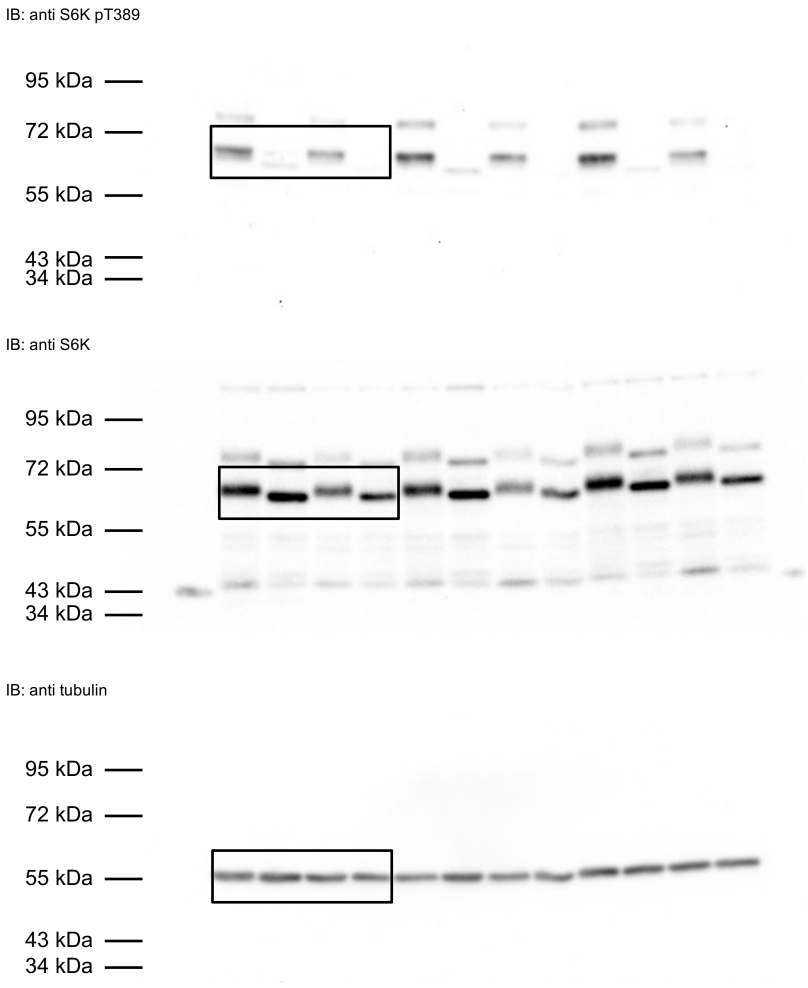
IB: anti S6K pT389
95 kDa
72 kDa
55 kDa
43 kDa
34 kDa
IB: anti S6K
95 kDa
72 kDa
55 kDa
43 kDa
34 kDa
IB: anti tubulin
95 kDa
72 kDa
55 kDa
43 kDa
34 kDa

Figure S5a

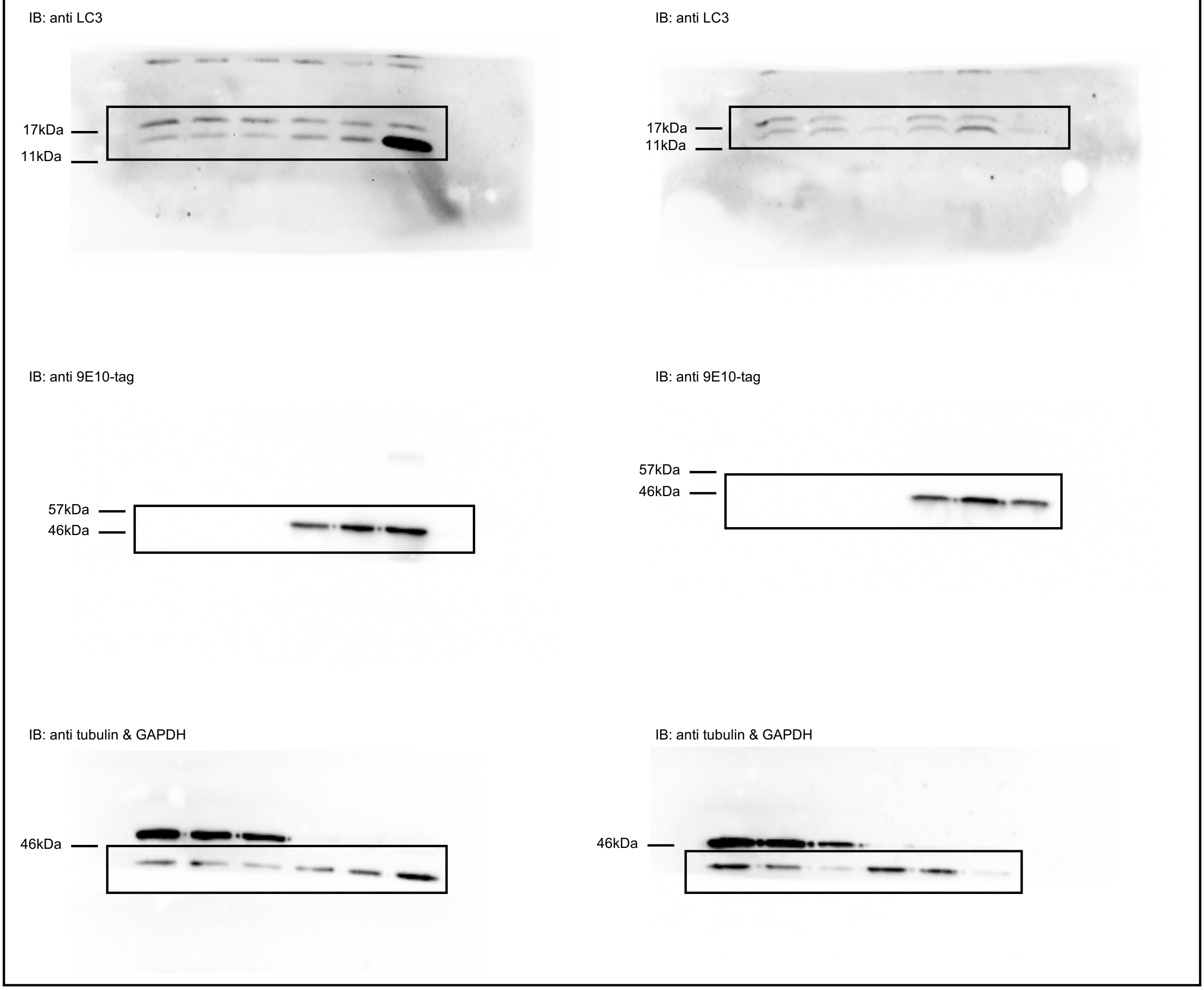
IB: anti LC3
17kDa
11kDa
IB: anti LC3
17kDa
11kDa
IB: anti 9E10-tag
57kDa
46kDa
IB: anti 9E10-tag
57kDa
46kDa
IB: anti tubulin & GAPDH
46kDa
IB: anti tubulin & GAPDH
46kDa

Figure S6f

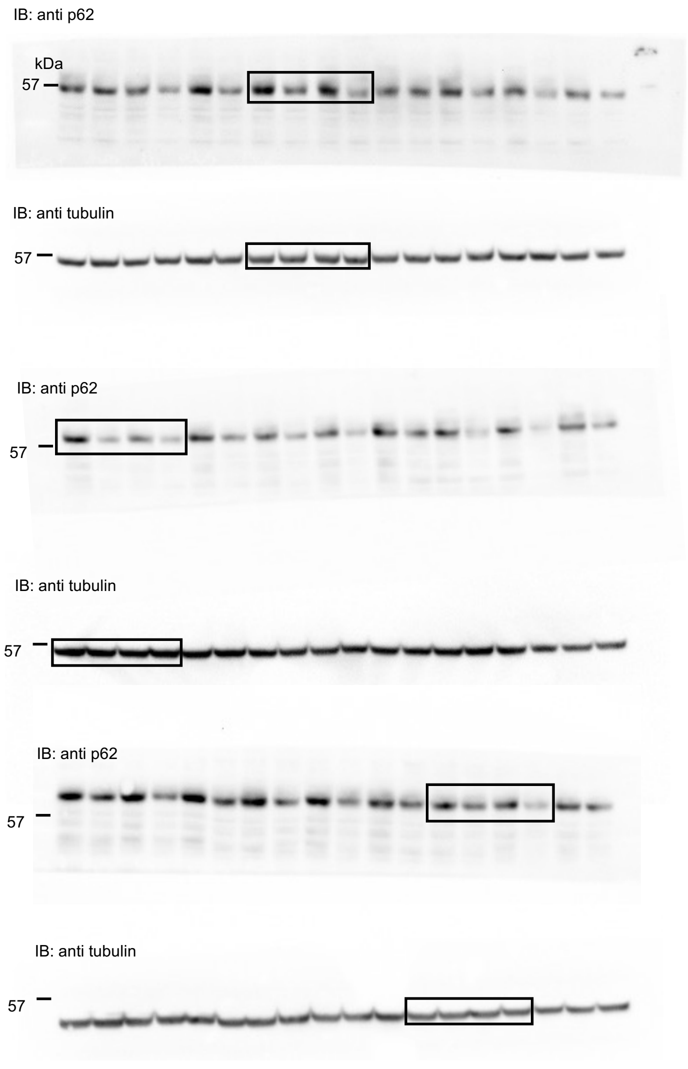

Figure S7a

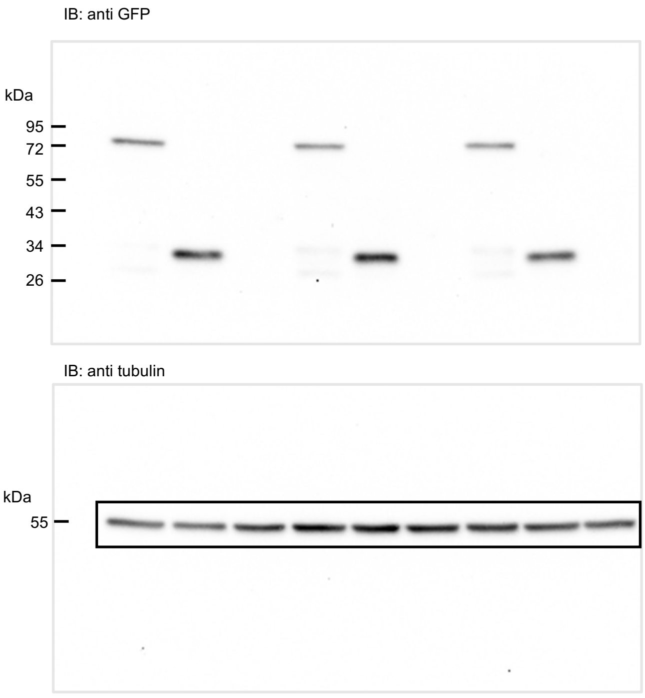

Figure S6c

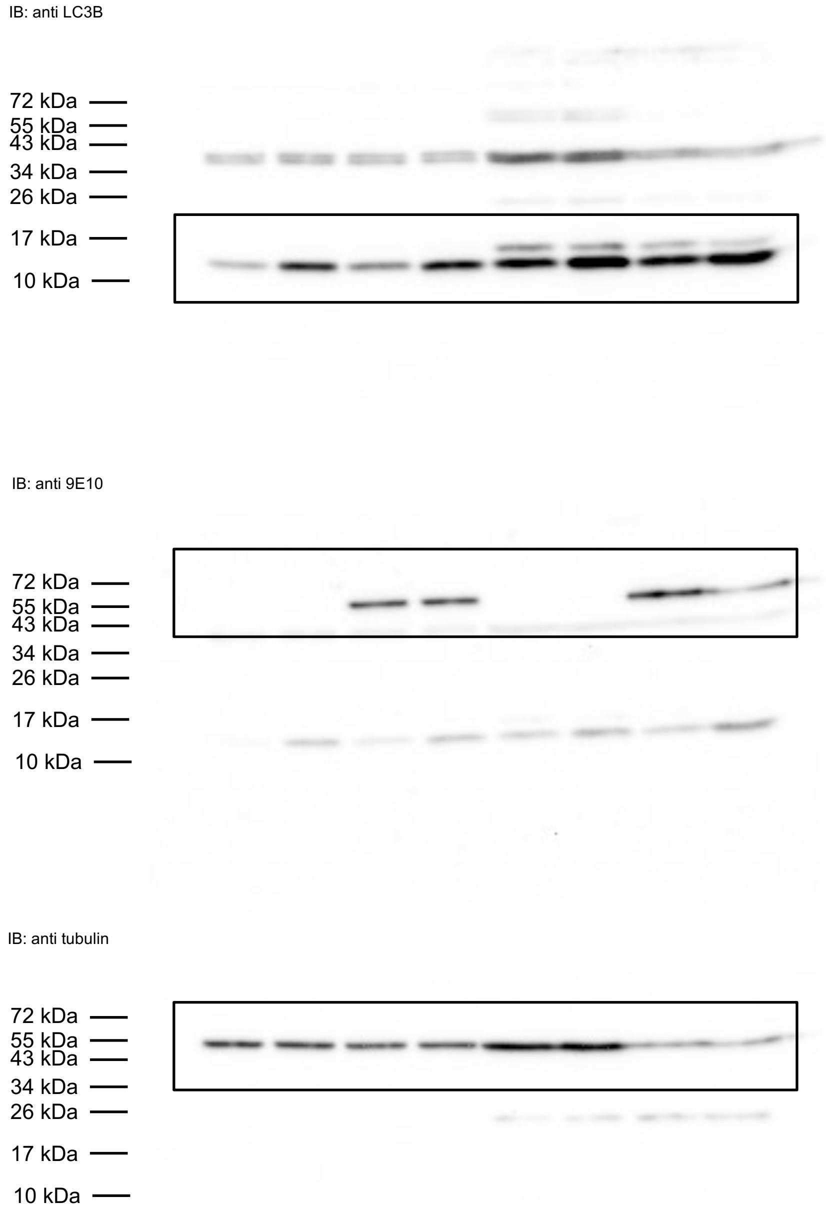
IB: anti LC3B
72 kDa
55 kDa
43 kDa
34 kDa
26 kDa
17 kDa
10 kDa
IB: anti 9E10
72 kDa
55 kDa
43 kDa
34 kDa
26 kDa
17 kDa
10 kDa
IB: anti tubulin
72 kDa
55 kDa
43 kDa
34 kDa
26 kDa
17 kDa
10 kDa

Supplementary Fig. 9: Uncropped Western blots. Image sections that are displayed in main and supplementary figures are indicated for each subfigure.
