# Supplementary material for: The ABL-MYC axis controls WIPI1-enhanced autophagy in lifespan extension
Source: Commun Biol. 2023 Aug 24;6:872. doi: 10.1038/s42003-023-05236-9 (PMC10449903; doi:10.1038/s42003-023-05236-9)
Supplement: Supplementary file 3 — Description of Additional Supplementary Files [file 42003_2023_5236_MOESM3_ESM.pdf]

Description of Additional Supplementary Files

File name: Supplementary Data 1
Description: The source data behind the graphs in the paper.

File name: Supplementary Video 1
Description: GFP-WIPI-1 decorated, dynamic omegasomes in starved U2OS cells. Stable U2OS GFP-WIPI-1 cells were starved by amino acid and serum deprivation (EBSS) and analysed by live-cell microscopy. Images were acquired 7 h and 28 min after starvation induction, and still images of selected time points are displayed in Fig. 5g.

File name: Supplementary Video 2
Description: WIPI1-positive autophagic membranes are present in TNTs. U2OS GFP-WIPI1 cells use TNTs to transfer GFP-WIPI1 puncta from one cell to the other. Z-stacks were acquired through confocal laser-scanning microscopy and used for 3D video presentation. A still image from this video is displayed in Fig. 6c (left panel).

File name: Supplementary Video 3
Description: LC3-positive autophagic membranes are present in TNTs. A video of 3D reconstructed z-stacks from confocal laser-scanning microscopy shows a TNT connecting U2OS cells stably expressing RFP-GFP-LC3 during intercellular transfer of autophagosomes. A still image from this video is displayed in Fig. 6c (right panel).

File name: Supplementary Video 4
Description: Live-cell microscopy of WIPI1-positive autophagic membranes in TNTs. Live time-series microscopy of U2OS cells stably expressing GFP-WIPI1 was performed for approximately 30 min while combining Airyscan superresolution of GFP fluorescence and brightfield imaging. Video presentation of GFP-WIPI1 puncta moving through a TNT connecting two cells. Scale bar: 20 µm. A still image from this video is displayed in Fig. 5f (left panel).

File name: Supplementary Video 5
Description: Tracking WIPI1-positive autophagic membranes in TNTs. Live time-series microscopy-derived video of GFP-WIPI1 (Video 4) was employed for tracking GFP-WIPI1 puncta using the plug-in MTrackJ in Fiji. Tracks of different GFP-WIPI1 puncta are displayed in distinct colours: 1 in red, 2 in violet, 3 in purple, 4 in green and 5 in yellow. Still images from this video are displayed in Fig. 5f (right panels).
