# Supplementary material for: The ABL-MYC axis controls WIPI1-enhanced autophagy in lifespan extension
Source: Commun Biol. 2023 Aug 24;6:872. doi: 10.1038/s42003-023-05236-9 (PMC10449903; doi:10.1038/s42003-023-05236-9)
Supplement: Supplementary file 10 — Reporting Summary [file 42003_2023_5236_MOESM10_ESM.pdf]

nature portfolio

Corresponding author(s): Tassula Proikas-Cezanne

Last updated by author(s): August 11, 2023

Reporting Summary

Nature Portfolio wishes to improve the reproducibility of the work that we publish. This form provides structure for consistency and transparency in reporting. For further information on Nature Portfolio policies, see our Editorial Policies and the Editorial Policy Checklist.

Statistics

For all statistical analyses, confirm that the following items are present in the figure legend, table legend, main text, or Methods section.

n/a	Confirmed	
☐	☒	The exact sample size (*n*) for each experimental group/condition, given as a discrete number and unit of measurement
☐	☒	A statement on whether measurements were taken from distinct samples or whether the same sample was measured repeatedly
☐	☒	The statistical test(s) used AND whether they are one- or two-sided<br>*Only common tests should be described solely by name; describe more complex techniques in the Methods section.*
☐	☒	A description of all covariates tested
☐	☒	A description of any assumptions or corrections, such as tests of normality and adjustment for multiple comparisons
☐	☒	A full description of the statistical parameters including central tendency (e.g. means) or other basic estimates (e.g. regression coefficient) AND variation (e.g. standard deviation) or associated estimates of uncertainty (e.g. confidence intervals)
☐	☒	For null hypothesis testing, the test statistic (e.g. *F*, *t*, *r*) with confidence intervals, effect sizes, degrees of freedom and *P* value noted<br>*Give P values as exact values whenever suitable.*
☒	☐	For Bayesian analysis, information on the choice of priors and Markov chain Monte Carlo settings
☒	☐	For hierarchical and complex designs, identification of the appropriate level for tests and full reporting of outcomes
☒	☐	Estimates of effect sizes (e.g. Cohen's *d*, Pearson's *r*), indicating how they were calculated

Our web collection on statistics for biologists contains articles on many of the points above.

Software and code

Policy information about availability of computer code

Data collection	IN Cell Analyser 1000 (GE Healthcare) workstation for lentiviral shRNA screen; Easy-nLC 1200 system coupled to a Q Exactive HF mass spectrometer (both Thermo Fisher Scientific) for Phospho-SILAC analysis.
Data analysis	IN Cell Analyser Workstation 3.4 software (GE Healthcare) for lentiviral shRNA screen; ConTra v3 for predicting transcription factor binding sites; MaxQuant software (version 1.5.2.8), and R (v 3.5.1) for Phospho-SILAC analysis; Webportal at Qiagen.com/geneglobe for autophagy pathway-focused gene expression analysis using qPCR arrays; R (version 3.6.0) package gprofiler2 (version 0.1.8) with a hypergeometric test and the default gSCS method for multiple testing correction, and clusterProfiler (version 3.12.0) R package for pathway enrichement analysis; Fusion Capt advance software (Vilber Lourmat) for quantitative Western blotting; ZEN software (ZEISS), Fiji Plug-in MTrackJ, and CellProfiler (Version 4.2.4) for fluorescence microscopy; OASIS 2 for C. elegans survival quantifications.

For manuscripts utilizing custom algorithms or software that are central to the research but not yet described in published literature, software must be made available to editors and reviewers. We strongly encourage code deposition in a community repository (e.g. GitHub). See the Nature Portfolio guidelines for submitting code & software for further information.

Data

Policy information about availability of data

All manuscripts must include a data availability statement. This statement should provide the following information, where applicable:

- Accession codes, unique identifiers, or web links for publicly available datasets
- A description of any restrictions on data availability
- For clinical datasets or third party data, please ensure that the statement adheres to our policy

Source data for Figures and Supplementary Figures are provided as Supplementary Data File with the article. The mass spectrometry proteomics data have been deposited to the ProteomeXchange Consortium via the PRIDE partner repository with the dataset identifier PXD023146. All other relevant data supporting the findings of this study are available on request.

Human research participants

Policy information about studies involving human research participants and Sex and Gender in Research.

Reporting on sex and gender	*Use the terms sex (biological attribute) and gender (shaped by social and cultural circumstances) carefully in order to avoid confusing both terms. Indicate if findings apply to only one sex or gender; describe whether sex and gender were considered in study design whether sex and/or gender was determined based on self-reporting or assigned and methods used. Provide in the source data disaggregated sex and gender data where this information has been collected, and consent has been obtained for sharing of individual-level data; provide overall numbers in this Reporting Summary. Please state if this information has not been collected. Report sex- and gender-based analyses where performed, justify reasons for lack of sex- and gender-based analysis.*
Population characteristics	*Describe the covariate-relevant population characteristics of the human research participants (e.g. age, genotypic information, past and current diagnosis and treatment categories). If you filled out the behavioural & social sciences study design questions and have nothing to add here, write "See above."*
Recruitment	*Describe how participants were recruited. Outline any potential self-selection bias or other biases that may be present and how these are likely to impact results.*
Ethics oversight	*Identify the organization(s) that approved the study protocol.*

Note that full information on the approval of the study protocol must also be provided in the manuscript.

Field-specific reporting

Please select the one below that is the best fit for your research. If you are not sure, read the appropriate sections before making your selection.

☒ Life sciences ☐ Behavioural & social sciences ☐ Ecological, evolutionary & environmental sciences

For a reference copy of the document with all sections, see nature.com/documents/nr-reporting-summary-flat.pdf

Life sciences study design

All studies must disclose on these points even when the disclosure is negative.

Sample size	No statistical method was used to predetermine sample size.
Data exclusions	Criteria are reported in Methods section for lentiviral shRNA screening procedures, phospho-SILAC analysis, and autophagy pathway-focused gene expression analysis using qPCR array.
Replication	The number of independent experiments performed is indicated in the figure legends. In general quantified experiments were repeated at least three times.
Randomization	In this study, automated procedures were chosen (such as image acquisition, image analysis). If this was not the case, no randomization was performed. However, the co-authors of this study were trained to be unbiased when making manual counts. Additionally, all raw data generated is open to all team members in a transparent manner and healthy, transparent discussion takes place to counteract bias.
Blinding	Most experiments were conducted independently by several coauthors or colleagues, and if possible, automated assessments have been employed. For standard FL or LSM no blinding occoured.

Reporting for specific materials, systems and methods

We require information from authors about some types of materials, experimental systems and methods used in many studies. Here, indicate whether each material, system or method listed is relevant to your study. If you are not sure if a list item applies to your research, read the appropriate section before selecting a response.

Materials & experimental systems

n/a	Involved in the study
☐	☒ Antibodies
☐	☒ Eukaryotic cell lines
☒	☐ Palaeontology and archaeology
☐	☒ Animals and other organisms
☒	☐ Clinical data
☒	☐ Dual use research of concern

Methods

n/a	Involved in the study
☒	☐ ChIP-seq
☒	☐ Flow cytometry
☒	☐ MRI-based neuroimaging

Antibodies

Antibodies used

The following description is taken from the Method section.

Primary antibodies
The following primary antibodies were used: c-Abl (Cell Signaling Technologies, 2862; WB: 1:1000), CrkL (Santa Cruz Biotechnologies, sc-319; WB: 1:1000), DDR1 (Cell Signaling Technologies, 5583; WB: 1:1000), ERK2 (Cell Signaling Technologies, 9108; WB: 1:1000), GAPDH (Santa Cruz Biotechnologies, sc-47724; WB: 1:1000), GABARAPL1 (Cell Signaling Technologies, 26632; WB: 1:1000), GFP (Roche, 11814460001; WB: 1:1000), LC3 (nanoTools, 0231-100/LC3-5F10; WB: 1:1000), c-myc (9E10) (Santa Cruz Biotechnologies, sc-40; WB: 1:1000), c-myc (Cell Signaling Technologies, 9402S; WB: 1:500), p62 (Santa Cruz Biotechnologies, sc-28359; WB: 1:1000, IF: 1:50), p62 (Medical and Biological Laboratories, PM045; WB: 1:500-1:1000), S6K (Cell Signaling Technologies, 2708, WB: 1:500-1:1000), TFEB (Cell Signaling Technologies, 37785, WB: 1:500), α-Tubulin (Sigma–Aldrich, T5168; WB: 1:50000, IF 1:2000) and ULK1 (Cell Signaling Technologies, 8054; WB: 1:500-1:1000).
The following phospho-specific primary antibodies were used: phospho-CrkL (Tyr207) (Cell Signaling Technologies, 3181S; WB: 1:1000), phospho-p44/42 ERK1/ERK2 (Y204/Y187) (Cell Signaling Technologies, 5726; WB: 1:1000), phospho-MAX (S11) (Thermo Fisher, PA5-97346; WB: 1:500), phospho-c-Myc (Ser62) (Cell Signaling Technologies, 13748; WB: 1:500), phospho-S6K (Cell Signaling Technologies, 9234, WB: 1:500-1:1000), phospho-TFEB (Ser211) (Cell Signaling Technologies, 37681, WB: 1:500) and phospho-ULK1 (Ser757) (Cell Signaling Technologies, 6888, WB: 1:500-1:1000).

Secondary antibodies
The following secondary antibodies were used: Alexa Fluor 488 goat anti-rabbit IgG (Life Technologies, A-11008, IF 1:200), anti-mouse IgG HRP-linked (Cell Signaling, 7,076; WB 1:5000-1:10000), and anti-rabbit IgG HRP-linked (Cell Signaling, 7,074; WB 1:5000-10000).

Validation

All primary antibodies were used according to the manufacturer's instructions (manufacturers, catalog numbers and dilutions used in the study are given in the Method section).

Eukaryotic cell lines

Policy information about cell lines and Sex and Gender in Research

Cell line source(s)

Human U2OS osteosarcoma cells (ATCC, HTB-96), human G361 malignant melanoma cells (ATCC; CRL-1424).

Authentication

U2OS and G361 cell lines were purchased from ATCC and not additionally authenticated.

Mycoplasma contamination

Cell lines tested negative for mycoplasma contaminations..Testing occured by using reagents from PromoKine (PK-CA91) and InvivoGen (MycoStrip™ Mycoplasma Detection Kit).

Commonly misidentified lines
(See ICLAC register)

Name any commonly misidentified cell lines used in the study and provide a rationale for their use.

Animals and other research organisms

Policy information about studies involving animals; ARRIVE guidelines recommended for reporting animal research, and Sex and Gender in Research

Laboratory animals

The following C. elegans strains were used in this study and provided by the CGC: N2 (wild-type/WT), VC893: atg-18(gk378), XR1: abl-1(ok171), DA2123: lgg-1::GFP+rol-6(su1006) and E. coli OP50. The abl-1(ok171);atg-18(gk378) and abl-1(ok171);gfp::lgg-1 strains were generated in this study.

Wild animals

Provide details on animals observed in or captured in the field; report species and age where possible. Describe how animals were caught and transported and what happened to captive animals after the study (if killed, explain why and describe method; if released, say where and when) OR state that the study did not involve wild animals.

Reporting on sex

Hermaphrodites were used in this study.

Field-collected samples	*For laboratory work with field-collected samples, describe all relevant parameters such as housing, maintenance, temperature, photoperiod and end-of-experiment protocol OR state that the study did not involve samples collected from the field.*
Ethics oversight	Work with C. elegans strains is not classified as animal testing.

Note that full information on the approval of the study protocol must also be provided in the manuscript.
