# Supplementary material for: The ABL-MYC axis controls WIPI1-enhanced autophagy in lifespan extension
Source: Commun Biol. 2023 Aug 24;6:872. doi: 10.1038/s42003-023-05236-9 (PMC10449903; doi:10.1038/s42003-023-05236-9)
Supplement: Supplementary file 1 — Peer Review File [file 42003_2023_5236_MOESM1_ESM.pdf]

nature portfolio

Peer Review File

The ABL-MYC axis controls WIPI1-enhanced autophagy in lifespan extension

Open Access This file is licensed under a Creative Commons Attribution 4.0 International License, which permits use, sharing, adaptation, distribution and reproduction in any medium or format, as long as you give appropriate credit to the original author(s) and the source, provide a link to the Creative Commons license, and indicate if changes were made. In the cases where the authors are anonymous, such as is the case for the reports of anonymous peer reviewers, author attribution should be to 'Anonymous Referee' followed by a clear attribution to the source work. The images or other third party material in this file are included in the article's Creative Commons license, unless indicated otherwise in a credit line to the material. If material is not included in the article's Creative Commons license and your intended use is not permitted by statutory regulation or exceeds the permitted use, you will need to obtain permission directly from the copyright holder. To view a copy of this license, visit http://creativecommons.org/licenses/by/4.0/.

Reviewers' comments:

Reviewer #1 (Remarks to the Author):

In the current study, Sporbeck et al., identified ABL1-ERK-MYC signaling axis is the novel negative regulator of WIPI1 expression and autophagy. They found that MYC directly binds to promoter of WIPI1 and repress it's expression. Inhibition of ABL1-ERK-MYC signaling increases WIPI1 and thus autophagy flux. WIPI1 overexpression suffices to induce autophagy. They also showed that the transfer of autophagosomes from autophagy-competent cells to autophagy-deficient cells. Moreover, authors showed that knockdown of abl-1 extends C. elegans lifespan through increasing atg-18 expression and autophagy, highlighting an evolutionally conserved role of ABL1/ABL-1-WIPI1/ATG-18 axis. Overall, this is a well conducted study with clear and novel results. There are some comments that should be addressed before the manuscript can be recommended for publication.

Major points
1. ABL1-ERK-MYC signaling axis regulates autophagy independently of mTORC1 pathway based on the phosphoproteomics? It is better to show knockdown of ABL1-ERK-MYC signaling indeed does not affect phosphorylation of mTORC1 substrates such asS6K, ULK1 TFEB(Ser211). In this case, Torin1 treatment further increases autophagy activity in ABL1 and/or DDR1 KD cells?

2. Authors need to show siABL1/2+siDDR1 is not additive with a single suppression of each to conclude that ABL and DDR1 function in the same pathway.

3. In Fig.2e, authors need to show the expression levels of ERK2 pY187 in siDDR1 conditions as well as siABL1.

4. Authors need to address whether MYC only affects WIPII or other ATGs including WIPI3, 4 as well.

5. In Fig.5, the connection between WIPI1 and TNT is not clear. Does increasing WIPI1 plasmid increase numbers of TNT and transferred autophagosomes?

6. In Fig.6, it is essential to show if longevity of abl-1 depletion is abolished by RNAi knockdown of other ATGs such as unc-51, atg-7 to conclude the true autophagy dependency.

7. In Fig.6e, authors need to show the comparison between WT vs abl-1 mutant to show atg-18 is indeed upregulated in abl-1 compared to WT. How about a WIPI3/4 homolog, epg-6? Is there conserved E-box in the promoter region of atg-18? Is C. elegans MYC homolog mml-1 work downstream of abl-1?

Minor points
1. In Fig. 3a, 'siMAX+siMAX' should be siMYC+siMAX ?

2. Authors mention that there is a significant increase in WIPI1 mRNA abundance with siDDR1, but no asterisk is marked in Fig. S3h.

3. In Fig.5J, GFP::WIPI1 dots are difficult to see.

4. GFP::LGG-1 dots are indeed increased in abl-1 mutants?

5. It is curious to know if overexpression of abl-1 shortens lifespan.

6. In Fig.6b, 'revovery' should be recovery.

Reviewer #2 (Remarks to the Author):

The beta-propellor protein Atg18 is a key component of autophagy machinery identified from yeast genetic screens. In mammals, Atg18 has four homologs, including of WIPI1-4. The function of WIPI2 and WIPI3/4 have been well-studied, and all of them are essential for autophagy and involved at different steps. However, the specific role and regulation of WIPI1 in autophagy are still undetermined. In this study, Sporbeck and his collaborators designed an shRNA screen targeting human kinases to identify potential regulators of WIPI1 puncta formation and discovered ABL1-ERK2-MYC axis as an inhibitory pathway to control WIPI1 expression. The authors further demonstrated that ATG-18, the worm homolog of WIPI1, was also negatively regulated by ABL1, and ABL1 deficiency extended the life span of C. elegans through increasing ATG-18 levels. Another intriguing finding is that overexpression of WIPI1 facilitated phagophore biogenesis, as well as intercellular tunneling nanotube formation to mediate autophagic membrane transport to neighboring autophagy-insufficient cells. This is an interesting paper which provides us more understandings about the function of WIPI1. However, there are some issues needed to be addressed before acceptance.

Major concerns:
1. In C.elegans, yeast Atg18 has only two homologs, ATG-18 and EPG-6, corresponding to mammalian WIPI1/2 and WIPI3/4, respectively. Hence, C.elegans is not an appropriate model to study the specific regulation of WIPI1 in vivo. The authors should use mammalian systems to address this question.
2. Does the elevated autophagy flux in ABL KD and DDR1 KD cells depend on inactivation of ERK2? Can ectopic expression of active ERK reversed the increased WIPI1 and LC3 puncta in ABL KD and DDR1 KD cells?
3. Since the authors claimed that Myc is the downstream factor of ERK inactivation, does overexpression of Myc reduce autophagy activity in WT, ABLKD or DDR1 KD cells?
4. Although the authors showed that Myc bound to the E-boxes and G9a sites in the WIPI1 promotor, further experiments, like luciferase assays, are needed to verify if the binding suppresses the transcription of WIPI1.
5. Most experiments were done using WIPI1-expressing cells, which were quite artificial. WIPI1 KO should be involved to verify the role of WIPI1 at endogenous levels. Does WIPI1 KO inhibit the autophagy induction induced by siABL or siDDR1? Does WIPI1 KO affect WIPI2 puncta formation under starvation conditions? Does WIPI1 KO inhibit the TNT formation?

Minor points:
1. As ABL2 KD also significantly increased GFP-WIPI1 puncta, why ABL2 was not shown in the screen list as ABL1? In some of the experiments, such as WIPI1 and LC3 puncta formation, the authors only showed the ABL1 or ABL2 single KD data, while in the other experiments, only ABL1 and ABL2 double KD were used. This inconsistency is quite confusing. The authors need to provide both single KD and double KD results side by side to show if ABL1 and ABL2 are indeed involved in autophagy regulation redundantly.
2. In Figure 3h, why didn’t G9A bind to the G9A-binding site?
3. In Figure 4a, the label should be 0.1, 0.25 and 0.5.

Reviewer #3 (Remarks to the Author):

The work of Sporbeck et. al. characterizes several aspects of the function of WIPI1, a protein whose role in the regulation of autophagy is not clearly established. First, they show that WIPI1 expression is controlled by the ABL1-ERK-MYC signaling pathway. They propose that increased WIPI1 gene expression potentiates autophagy and that autophagic structures can travel between cells through tunneling nanotubes. Finally they show that ABL1 deficiency in C. elegans increases WIPI1 gene expression and prolongs lifespan in this model. These results are interesting and well supported by

data, but some conclusions and functional connections are overstated with the available data and should be nuanced and discussed more clearly. Data presentation and statistical analysis should also be revised.

1. Many of the experiments rely on puncta counts. Sometimes the data is presented as "% of cells with puncta" and others as "number of puncta per cell". In the first case, does it mean % of cells with at least one puncta? or there is a threshold of puncta (or signal) from which a cell is considered "with puncta"? The data would be more consistent and comparable if the same measurement were shown. If this is not possible at least describe clearly what is shown, how the measurement was performed, as well as the number of experiments and/or the number of cells used per experiment.

2. In this regard, the statistical tests used in puncta count experiments should be revised in each experiment. For example in Figure S2d: the significance of the analysis of GFP-LC3 puncta/cell has been analyzed using two-tailed heteroscedastic t test, which should be applied for data following a normal (parametric) distribution. Number of puncta per cell is likely to follow a non-normal distribution and therefore it would be appropriate to use non-parametric tests as the Kruskal -Wallis used for example in Fig 4c or FigS2n, where individual counts are displayed.

3. More in this regard, Fig. 4C is difficult to understand. It seems that % of cells with puncta is being measured. If so what are the individual points? these individual points range from 100% to 0% ??The axis should be puncta/cells?

Also, for all figures where puncta/cell is displayed (for example Fig S2f), the individual data count (as in Fig S2n) would be more appropriate than bars, and the number of cells used for quantification should be indicated. In the case of Fig S2f is not clear what n=6 means, only six cells analyzed?

4. Several experiments are shown that support the idea that increased expression of WIPI1 enhances autophagy. In my opinion, the data only support an increased number of puncta and the formation of large perinuclear autophagosomal structures, but no data on autophagy flux are provided. The presence of abnormal elongated structures is not evidence that autophagy is physiologically increased. The 4h experiment does not distinguish between the disassembly of this aberrant structure or the actual progression of autophagosomes. Perhaps the use of a WIPI1 KO cell line into which a WIPI1 expression plasmid can be introduced may provide clearer information on whether or not WIPI1 potentiates autophagy flux physiologically. At the very least, the data need to be discussed and the conclusions nuanced.

5. The authors attempt to connect the function of WIPI1 to tunneling nanotube (TNT) activity. It is stated in the abstract that "increased expression of the WIPI1 gene enhances the formation of autophagic membranes capable of migrating through tunneling nanotubes to neighboring cells with low autophagic activity". In my opinion this sentence is misleading as it leads to the idea that WIPI1 is required to generate a specific structures capable of trafficking between cells. These aspects may only be connected phenomenologically (just because they coincide in a cell). Many different types of vesicles, including autophagosomes, are expected to be able to travel across TNTs. To support a specific functional connection of this phenomenon to WIPI1 function, a number of questions need to be addressed: for example, does the presence of TNTs depend on WIPI1? Is there preferential transport of WIPI1-containing structures? The authors should clearly discuss the limits of this observation and qualify their conclusions.

6. In the same line: Figure 6f. The longevity experiments have been performed in C. elegans, not in mammalian models or humans. Therefore, the human symbol should be changed to that of a worm.

Point-to-point response to reviews on the manuscript

First and foremost, we would like to sincerely thank the editor and all three reviewers for their time and expertise in offering important and insightful comments, questions, and suggestions for improvements regarding our manuscript.

Following the recommendations of the editor and the reviewers' guidance, we performed an extensive set of additional experiments that enabled us to significantly improve our manuscript.

We now present here a revised version of our original manuscript that includes important experimental additions and improvements. Accordingly, we have highlighted in yellow the descriptions of the new results in the revised manuscript text.

All the points raised by the reviewers are addressed below.

Reviewer #1 (Remarks to the Author):

In the current study, Sporbeck et al., identified ABL1-ERK-MYC signaling axis is the novel negative regulator of WIPI1 expression and autophagy. They found that MYC directly binds to promoter of WIPI1 and repress it's expression. Inhibition of ABL1-ERK-MYC signaling increases WIPI1 and thus autophagy flux. WIPI1 overexpression suffices to induce autophagy. They also showed that the transfer of autophagosomes from autophagy-competent cells to autophagy-deficient cells. Moreover, authors showed that knockdown of abl-1 extends *C. elegans* lifespan through increasing atg-18 expression and autophagy, highlighting an evolutionally conserved role of ABL1/ABL-1-WIPI1/ATG-18 axis. Overall, this is a well conducted study with clear and novel results. There are some comments that should be addressed before the manuscript can be recommended for publication.

We are deeply grateful to the reviewer for the encouraging comments on the quality of our manuscript. This evaluation gave us a lot of inspiration and the reviewers' guidance prompted us to set up a series of important new experiments, which have led us to improve our study based on the advice offered. In the following we provide details on the approaches we used to meet the reviewer's requests.

Major points

1. ABL1-ERK-MYC signaling axis regulates autophagy independently of mTORC1 pathway based on the phosphoproteomics? It is better to show knockdown of ABL1-ERK-MYC signaling indeed does not affect phosphorylation of mTORC1 substrates such asS6K, ULK1 TFEB(Ser211). In this case, Torin1 treatment further increases autophagy activity in ABL1 and/or DDR1 KD cells?

We absolutely agree with the reviewer's important point. As suggested, we have devoted ourselves intensively to this important point and carried out quantitative Western blotting regarding the phosphorylation of mTORC1 substrates, summarized in the new Figure S4b-l and detailed in the following:

- TFEB pS211 was unchanged in ABL1/2KD and DDR1 KD settings, and ABL1/2+DDR1 KD had no additive effect either (**new Fig. S4b**).

- ULK1 p758 was unchanged in ABL1/2 KD and DDR1 KD settings, in both presence and absence of Torin 1 (**new Fig. S4c, d**).
- S6K pT389 was unchanged in ABL1/2 KD and DDR1 KD settings, in both presence and absence of Torin 1 (**new Fig. S4c, e**)
- In support, decreased p62 levels in ABL1/2 KD and DDR1 KD settings were further reduced upon Torin 1 administration (**new Fig. S4f**)

We paralleled this assessment by downregulating either ERK2 or MYC and show that:

- ULK1 p758 (Fig. S4h) and S6K pT389 (**new Fig. S4i**) were unchanged in ERK2 KD, in both presence and absence of Torin 1.
- ULK1 p758 (Fig. S4j) and S6K pT389 (**new Fig. S4k**) were unchanged in MYC KD, in both presence and absence of Torin 1.

Our new results are consistent with our original phosphoproteomics analysis and strengthen the notion that altered mTORC1 signaling does not account for changes in WIPI1-enhanced autophagy. Nevertheless, it was essential to provide a comprehensive answer to this question and we are happy to have been given the opportunity during this revision.

In addition to the display in the above-stated figures, our new results are described in the revised result section (**page 8-9**) to read as follows (new text highlighted in yellow in the revised manuscript text):

*In addition, although our phospho-proteomics analysis showed no change in the TORC1-TFEB route, we analysed several mTOR targets by quantitative Western blotting, including TFEB, in the settings ABL1/2 KD, DDR1 KD, ERK2 KD and MYC KD (**Fig. S4 b-l**). Down-regulation of neither ABL1/2 nor DDR1 altered TFEB phosphorylation at the mTOR target site S211 (**Fig. S4b**), and ABL/DDR1 KD had no additive effect on TFEB pS211 either (**Fig. S4b**). It is also worth noting in this context that ABL1/2 KD and DDR1 KD also had no effect on the mTOR target site ULK1 pS758, which serves as a switch for autophagy initiation, while treatment with the specific mTOR inhibitor Torin 1, as expected, resulted in a significantly reduced level of ULK1 pS758 (**Fig. S4c, d**). Likewise, the phosphostatus of S6K pT389 remained unchanged in the ABL1/2 KD and DDR1 KD settings (**Fig. S4c, e**). In line with these results, downregulation of neither ERK2 nor c-MYC altered the phosphostatus of ULK1 pS758 or S6K pT389 (**Fig. S4g-k**). The significant changes in the ABL-ERK-MYC axis detected by our phosphoproteome analysis are therefore not to be classified in the context of the TORC1 signaling pathway and therefore the following experiment is also to be understood in full agreement with this hypothesis. When we downregulated ABL1/2 or DDR1, we showed that the autophagic flux was increased, as indicated by decreased p62 levels (**Fig. 1g, h**). Now, when we performed this experiment in the presence of Torin 1, p62 levels decreased significantly further (**Fig. S4f**), suggesting that ABL-ERK-MYC and TORC1 signalling are additive pathways in controlling the autophagic flux. In this context, we confirmed that ABL1/2 and DDR1 do not display an additive effect on p62 degradation (**Fig. S4l**).*

2. Authors need to show siABL1/2+siDDR1 is not additive with a single suppression of each to conclude that ABL and DDR1 function in the same pathway.

We thank the reviewer very much to for bringing this critical point to our attention. In the context of responding to major point 1, described in the above, we demonstrate that siABL1/2+siDDR1 was not additive regarding TFEB pS211 (**new Fig. S4b**). In addition, we conducted p62 Western blotting and provide a representative result demonstrating that there is no additive effect either (**new Fig. S4m**). These results are described in the revised result section (page 8-9) and are provided here in the above paragraph (*red letters in the above paragraph*).

3. In Fig.2e, authors need to show the expression levels of ERK2 pY187 in siDDR1 conditions as well as siABL1.

We thank the reviewer for pointing out this lack of confirmation. Accordingly, we repeated the confirmation of SILAC-based quantitative proteomics, as shown for ABL1/2 KD (original Fig. 2e). With our new results, in which we used both ABL KD and DDR1 KD settings, we show that ERK phosphorylation is also significantly increased in DDR1 KD settings (**new Fig S4a**).

We recorded this in the revised version of the manuscript as follows (**page 7**, new text highlighted in yellow in the manuscript text):

*Notably, we also confirmed a significant increase in ERK2 Y187 phosphorylation by Western blotting in the ABL1 KD setting (**Fig. 2e**) as well as in DDR1 KD settings (**Fig. S4a**).*

4. Authors need to address whether MYC only affects WIPII or other ATGs including WIPI3, 4 as well.

We fully agree with the reviewer on this statement and have therefore approached this question first in an unbiased fashion. As used for ABL KD and DDR1 KD settings, we used the human autophagy-focused gene expression array (n=3 in duplicates), now in MYC KD settings. This revealed that WIPI1 was again significantly upregulated (**new Fig. S5a**) like in ABL KD (original Fig. S3c) and DDR1 KD (original Fig. S3f) settings. In addition, as already shown for settings ABL KD (original Fig. S3d) and DDR1 KD (original Fig. S3g), additional ATG genes were upregulated (**new Fig. S5a**). Since only WIPI1 was included in the autophagy-focused gene expression array, we also tested all four WIPIs with regard to their gene expression, in MYC KD settings (**new Fig. S5b**), as well as in ABL KD (**new Fig. S3e**) and DDR1 KD (**new Fig. S3h**) settings. While WIPI2 and WIPI3 were unaffected in all settings, WIPI4 gene expression was also increased in the ABL KD and MYC KD settings, but to a lesser extent, particularly in MYC KD settings (**new Fig. S5b**).

As shown in the context of other transcription factors (e.g. FOXO, TFEB), the ABL-MYC axis may also represent a network-like control of autophagy, which will be of special interest for further future studies. Nevertheless, since - overlapping in all conditions - DDR1 KD, ABL KD, and MYC KD, WIPI1 turned out to be significantly increased in our unbiased approach, we concentrated on the further characterization of WIPI1 in this study.

Our new results are described in the revised result section (**page 9**) to read as follows (new text highlighted in yellow in the manuscript text):

*Furthermore, we also pursued this question in an unbiased manner using the human autophagy pathway-focused gene expression array with 84 genes containing WIPI1 that we already used for ABL1/2 KD and DDR1 KD settings (**Fig. S3**). Again, WIPI1 mRNA was significantly up-regulated and scored as a positive hit on MYC KD (**Fig. S5a**). Likewise, as in ABL1/2 KD and DDR1 KD settings, additional ATG genes were upregulated after downregulation of MYC (**Fig. S5a**), and in overlap with the ABL1/2 KD phenotype, WIPI4 was upregulated, albeit to a much lesser degree compared to WIPI1 (**Fig. S3e, S5b**). As in the ABL1/2 KD and DDR1 KD settings, WIPI2 and WIPI3 are not significantly upregulated (**Fig. S3e, h; S5b**). Since the upregulation of WIPI1 was an overlapping result after ABL1/2 KD, DDR1 KD and MYC KD (**Fig. S3; 3a; S5a, b**), and this being most pronounced compared to the other WIPI genes (**Fig. S3e, h; S5b)**, we subsequently focused on further characterizing WIPI1 gene expression in the context of the ABL-MYC signalling axis.*

5. In Fig.5, the connection between WIPI1 and TNT is not clear. Does increasing WIPI1 plasmid increase numbers of TNT and transferred autophagosomes?

We can only underline that the reviewer has formulated a very important remark, for which we thank sincerely. Accordingly, we have transfected U2OS cells with increasing WIPI1 plasmid concentrations (0.1, 0.25 μg) and quantified the numbers of TNTs formed (TNT index). While the lower concentration (0.1 μg) of transfected WIPI1 plasmid had no effect on THT formation, indeed, we observed a significant increase of TNTs when we introduced 0.25 μg of the WIPI1 plasmid (**new Fig. 6e**). In line with this finding, we further employed

WIPI1 KO cells that we generated during this revision (see also later in the text), and found that in the absence of WIPI1, TNT formation was significantly reduced (**new Fig. 6f**). Combined these results suggest that WIPI1 impacts TNT formation.

Motivated by this result, and based on the reviewer's important request, we transfected a coculture of donor cells expressing GFP-LC3 and recipient cells with NLS-mScarlet marked nuclei with increasing WIPI1 plasmid concentrations (0.2, 0.4, 0.6 μg). We then counted the number of recipient cells harbouring GFP-LC3 puncta and observed that increasing WIPI1 plasmid concentrations (0.6 μg) promoted autophagosome transfer (**new Fig. 6l**). This observation now links enhanced WIPI1 expression with enhanced autophagic flux (see also later in the text) and with an increase in transfer of autophagic membranes through TNTs.

These results are described in the revised result section (**page 13-14**) to read as follows (new text highlighted in yellow in the manuscript text):

*Furthermore, we found that the presence or absence of WIPI1 affects TNT formation (**Fig. 6e, f**). Transient overexpression of GFP-WIPI1 significantly increased the number of TNTs (**Fig. 6e**), while WIPI1 deficiency significantly decreased the number of TNTs and starvation-induced TNT formation was significantly attenuated in U2OS cells (**Fig. 6f; S8c, d**).*

*In this context, it is conceivable that an enhancing role of WIPI1 in the formation of autophagic membranes should therefore also influence the extent of autophagic membrane transport by TNTs. This assumption turned out to be relevant since the transfer of GFP-LC3 positive autophagosomes was significantly increased when WIPI1 was overexpressed (**Fig. 6l, m**).*

6. In Fig.6, it is essential to show if longevity of abl-1 depletion is abolished by RNAi knockdown of other ATGs such as unc-51, atg-7 to conclude the true autophagy dependency.

An important question has been raised and we set out to respond based on the following considerations.

As first evidenced by the Levine laboratory, *atg-18* mutant nematodes are short-lived (Meléndez A, Tallóczy Z, Seaman M, Eskelinen EL, Hall DH, Levine B. Autophagy genes are essential for dauer development and life-span extension in *C. elegans*. Science. 2003; 301(5638):1387-91). In fact, ATG-18-deficient nematodes appeared to affect lifespan more than *unc-51* mutant nematodes in the study by Toth and colleagues (Tóth ML, Sigmond T, Borsos E, Barna J, Erdélyi P, Takács-Vellai K, Orosz L, Kovács AL, Csikós G, Sass M, Vellai T. Longevity pathways converge on autophagy genes to regulate life span in *Caenorhabditis elegans*. Autophagy. 2008; 4(3):330-8). As for ATG-7 in *C. elegans*, deficiency had no impact on the lifespan of wild type nematodes (N2), but was associated with dietary restriction (Jia K, Levine B. Autophagy is required for dietary restriction-mediated life span extension in *C. elegans*. Autophagy. 2007; 3(6):597-9).

Based on these considerations, we applied RNAi targeting unc-51 in ABL-deficient nematodes and could show that the long-lived phenotype of ABL-1-deficient nematodes was indeed significantly reduced (**new Fig. 8b, new Fig. S9b**). This new result is consistent with our previous result showing ATG-18 dependence for the long-lived phenotype of ABL-1-deficient nematodes (Fig. 8a).

Our new result on UNC-51 is described in the revised result section (**page 15**) to read as follows (new text highlighted in yellow in the manuscript text):

*We have also confirmed that lifespan extension due to ABL-1 deficiency is dependent on autophagy, since depleting UNC-51, the ULK homologue in C. elegans, in the ABL-1 deficient strain by RNA interference (abl-1(ok171); unc-51 (RNAi)) counteracted lifespan extension (**Fig. 8b; S9b**).*

7. In Fig.6e, authors need to show the comparison between WT vs abl-1 mutant to show atg-18 is indeed upregulated in abl-1 compared to WT.

We thank you very much for this important objection and agree with the reviewer that this result was presented with insufficient clarity in the original manuscript.

Therefore, we repeated and extended this time course to three time points for assessing atg-18 expression in adult nematodes (day 1, day 6, day 11) using wild type (N2) and ABL-1 deficient mutants. We demonstrate that atg-18 expression is not increased over time in wild type nematodes (**new Fig. 7e, left panel**), while it is the case when comparing day 1 with day 6 or with day 11 of adulthood in ABL-1 deficient mutants (**new Fig. 7e, middle panel**). The direct comparison between wild type and ABL-1 deficient mutants of day 11, confirms that on day 11, atg-18 expression is significantly elevated in ABL-1 deficient mutants when directly compared to wild type nematodes (**new Fig. 7e, right panel**).

Therefore, we repeated this time course and extended it to three time points to assess atg-18 expression in adult nematodes (day 1, day 6, day 11) using wild-type (N2) and ABL-1-deficient mutants. We show that atg-18 expression does not increase over time in wild-type nematodes (**new Fig. 7e, left panel**). However, this was the case for ABL-1-deficient mutants, which exhibit increasing atg-18 expression over time (**new Fig. 7e, middle panel**). Direct comparison between wild-type and ABL-1-deficient mutants at day 11 confirms that atg-18 expression is indeed significantly increased in ABL-1-deficient mutants (**new Fig. 7e, right panel**).

This new comparison is described in the revised result section (**page 15**) to read as follows (new text highlighted in yellow in the manuscript text):

*Further, to determine whether we could observe a difference in ATG-18 gene expression in ABL-1-deficient nematodes, as we did for WIPI1 in human cells, we examined ATG-18 mRNA levels at three different time points in the lifespan of C. elegans, day 1, day 6 and day 11 of adulthood. Indeed, we observed a significant increase in ATG-18 mRNA levels from day 1 to day 6 as well as to day 11 in adult nematodes lacking ABL-1 function (**Fig. 7e, middle panel**), such increase was not observed in wild-type nematodes (**Fig. 7e, left and right panels**).*

How about a WIPI3/4 homolog, epg-6?

We thank the reviewer for this important question. We previously compared the effect on lifespan of ATG-18 and EPG-6 deficient adult nematodes and found that EPG-6 deficiency actually leads to prolonged lifespan in nematodes (Takacs Z, Sporbeck K, Stoeckle J, Prado Carvajal MJ, Grimmel M, Proikas-Cezanne T. ATG-18 and EPG-6 are Both Required for Autophagy but Differentially Contribute to Lifespan Control in *Caenorhabditis elegans*. Cells. 2019; 8(3):236). In this study, we proposed that EPG-6 may have an additional, presumably autophagy-independent, function that controls lifespan.

While this is surprising, it has been reported that the deficiency of some ATGs in nematodes leads to an extended lifespan (Hashimoto Y, Ookuma S, Nishida E. Lifespan extension by suppression of autophagy genes in *Caenorhabditis elegans*. Genes Cells. 2009; 14(6):717-26). In this context, it is worth mentioning that in the current revision carried out on the basis of the reviewer's suggestion, we found that ABL1/2 deficiency also increased WIPI4 expression in human cells. This association now provides an interesting basis for investigating the link between ABL and WIPI4 or EPG-6 in follow-up studies.

Is there conserved E-box in the promoter region of atg-18?

We have pursued this interesting question in the course of the revision and asked whether we can find in the putative atg-18 promoter homologous sequences to the E-boxes (M+1, M11, M19) that we have identified in the WIPI1 promoter.

Indeed, as shown below, we have identified two promising sites in the atg-18 promoter that may share functional similarity to the E-boxes in the WIPI1 promoter. However, we have refrained from including such speculation in the text of our revised version of the manuscript and hope the reviewer will appreciate that this point would require initiating a thorough characterization of the atg-18 promoter, which is beyond the scope of this study.

Nevertheless, the homology found between the E-boxes (see below) underlines that the ABL signalling pathway, which regulates WIPI1 or atg-18 expression, should be conserved in human cells and in *C. elegans*. However, since there is no clear homologue to human MYC in *C. elegans* (see next section) a new study is required to characterize this signalling axis in *C. elegans*.

```
ATG-18    AACGTTTTTGCGCGCGCCACCAACGTTTGCGCATGTGTTACTTGAGGCGGGTAGTCGACT  -1800
M19       ---------------------------TCCGCATGGG-----------------------     10
M11       ---------------------------TACACATGGC-----------------------     10
M+1       ------------------------------------------------------------

ATG-18    AACAATTCGGACACACAAAACCATCATACTGCAGTGAAGTTAAAAGTCCACCTCGAGGTG   -660
M19       ------------------------------------------------------------
M11       ------------------------------------------------------------
M+1       ----------------------------------------------TCCGCCTCGA----     10
```

Is *C. elegans* MYC homolog mml-1 work downstream of abl-1?

We are also very grateful to the reviewer for raising the question of a MYC homologue in *C. elegans*. In fact, in the course of our study, we had dealt with this question and noted the following connection in the literature (**referenced in the revised version of our manuscript**):

- Pickett CL, Breen KT, Ayer DE. A C. elegans Myc-like network cooperates with semaphorin and Wnt signaling pathways to control cell migration. Dev Biol. 2007 Oct 15;310(2):226-39.
- McFerrin LG, Atchley WR. Evolution of the Max and Mlx networks in animals. Genome Biol Evol. 2011;3:915-37.
- Johnson DW, Llop JR, Farrell SF, et al. The *Caenorhabditis elegans* Myc-Mondo/Mad complexes integrate diverse longevity signals. PLoS Genet. 2014 Apr;10(4):e1004278.

These studies describe that the MML-1 signaling pathway overlaps only in part with MYC functions in humans, and MML-1 appears to have additional functions. Due to unanswered questions about the function of MML-1 as a MYC homologue, we initially refrained from studying the MML-1 signaling pathway in *C. elegans*.

However, motivated by the reviewer, we now applied RNAi targeting mml-1 in wild type nematodes and found that lifespan was significantly increased, but the effect was nevertheless minor (**new Fig. 8b**). This circumstance is actually not surprising, since MML-1 exerts only partial MYC functions in *C. elegans*. Since the MML-1 signalling pathway in *C. elegans* still raises many open questions, we have not yet made the connection to ABL-1 in the study presented here, because we would have to include a larger number of factors, in addition to MML-1, in such an analysis. We hope that the reviewer can accept our reasoning that such an analysis exceeds the scope of our study.

Nevertheless, our data displaying a slight increase in life span in MML-1 deficient nematodes are compatible with the conclusion of our study here, and our new result is described on **page 15/16** to read as follows (new text highlighted in yellow in the manuscript text):

*Furthermore, we show by RNA interference in wild-type nematodes that the lack of MML-1, which shares some functional similarities with mammalian MYC [78-80], also contributes to an extended lifespan, but to a much lesser extent than ABL-1-deficient C. elegans (**Fig. 8b; S9b**).*

Minor points

1. In Fig. 3a, 'siMAX+siMAX' should be siMYC+siMAX ?

We thank the reviewer for this comment. We have corrected this point in the revised version of our manuscript (original Fig. 3a). Indeed, the data mentioned showed siMYC + siMAX.

2. Authors mention that there is a significant increase in WIPI1 mRNA abundance with siDDR1, but no asterisk is marked in Fig. S3h.

We have extended the assessment of WIPI gene expression to include WIPI1, WIPI2, WIPI3 and WIPI4 in DDR1 KD settings, and show that indeed, WIPI1 levels go significantly up, while WIPI2, WIPI3 and WIPI4 levels are unaffected (**new Fig. S3h**). The asterisk is now included.

3. In Fig.5J, GFP::WIPI1 dots are difficult to see.

We fully agree that in the original image GFP-WIPI1 was difficult to see. We have replaced this image with another representative image taken from the same experiment (**new image pane**l in Fig. 6k and Fig. S8e).

4. GFP::LGG-1 dots are indeed increased in abl-1 mutants?

We feel grateful for this question as it motivated us to perform a quantitative image-based GFP::LGG1 puncta analysis. For this purpose we chose the same experimental setup that we already used for the quantitative Western blotting of GFP::LGG-1 (Fig. 7c). In fact, we found that both GFP::LGG1 puncta numbers and size increased significantly in ABL-1 deficient nematodes (**new Fig. 7d**). This result supports our finding that the autophagic flux is increased in the absence of ABL-1 in *C. elegans* (Fig. 7c).

This result is described in the revised result section (**page 15**) to read as follows (new text highlighted in yellow in the manuscript text):

*In line with this result, both the number and size of GFP-LGG1 puncta increased significantly in abl-1(ok171) nematodes carrying a GFP::LGG1 reporter transgene (**Fig. 7d**).*

5. It is curious to know if overexpression of abl-1 shortens lifespan.

We wholeheartedly agree on this to be an important issue. However, during this revision, we have focused more on extending the evaluation of the consequences of WIPI1 overexpression regarding enhancement of autophagy and TNT transfer in human cells during this revision. Furthermore, we offer new and extensive experiments in *C. elegans*, which are shown in the **new Fig. 7d, 7e and 8b**. We hope the reviewer can accept, that while we were unable to approach this point experimentally in the course of this study, we have in fact been able to respond to most points raised collectively by all three reviewers.

6. In Fig.6b, 'revovery' should be recovery.

We thank the reviewer for this correction, which we have made in the revised version of the caption of Fig. 6b.

Reviewer #2 (Remarks to the Author):

The beta-propellor protein Atg18 is a key component of autophagy machinery identified from yeast genetic screens. In mammals, Atg18 has four homologs, including of WIPI1-4. The function of WIPI2 and WIPI3/4 have been well-studied, and all of them are essential for autophagy and involved at different steps. However, the specific role and regulation

of WIPI1 in autophagy are still undetermined. In this study, Sporbeck and his collaborators designed an shRNA screen targeting human kinases to identify potential regulators of WIPI1 puncta formation and discovered ABL1-ERK2-MYC axis as an inhibitory pathway to control WIPI1 expression. The authors further demonstrated that ATG-18, the worm homolog of WIPI1, was also negatively regulated by ABL1, and ABL1 deficiency extended the life span of C. elegans through increasing ATG-18 levels. Another intriguing finding is that overexpression of WIPI1 facilitated phagophore biogenesis, as well as intercellular tunneling nanotube formation to mediate autophagic membrane transport to neighboring autophagy-insufficient cells. This is an interesting paper which provides us more understandings about the function of WIPI1. However, there are some issues needed to be addressed before acceptance.

We would like to thank the reviewer extensively for the important review and for the positive classification of our study in the context of the role of WIPI proteins in autophagy. We are thankful for the reviewer's insightful suggestions, which led us to set up new types of experiments on specific issues. We will now detail our experimental developments based on the questions raised.

Major concerns:

1. In *C. elegans*, yeast Atg18 has only two homologs, ATG-18 and EPG-6, corresponding to mammalian WIPI1/2 and WIPI3/4, respectively. Hence, *C. elegans* is not an appropriate model to study the specific regulation of WIPI1 *in vivo*. The authors should use mammalian systems to address this question.

We are truly grateful to the reviewer for pointing out that functional relationships between yeast (Atg18, Atg21, Hsv2), *C. elegans* (ATG-18, EPG-6) and mammalian systems (WIPI1 through WIPI4) regarding the PROPPIN family of proteins is complex. This complex relationship became apparent immediately after the cloning of the human WIPI members (Proikas-Cezanne et al., Oncogene 2004) and has accompanied us ever since. Naturally, we would be very excited to apply our newly acquired knowledge about the role of WIPI1 to the mammalian system.

However, as the Editor pointed out in an advisory capacity, such an approach is beyond the scope of the present study. We therefore took up this point and followed the Editor's recommendation to remark on the principal limitations of use with the model organism *C. elegans*. This reads as follows in the revised version of our manuscript on **page 17/18** (new text highlighted in yellow in the manuscript text):

However, it should be noted that our presented lifespan data in the context of the ABL-MYC-WIPI1 axis, was carried out in the model organism C. elegans. Hence the question arises as to whether the outcome can be transferred to the organismic complexity of the aging process in humans.

Nevertheless, we strongly feel that we can learn from our approach in *C. elegans* that the ABL-1 axis controlling ATG-18 expression is relevant for the regulation of autophagy and lifespan in a physiological context. Our new data using *C. elegans* at the request of reviewer #1 underline that the principle of this axis may in fact be conserved (**new Fig. 7d, e; new Fig. 8b**).

2. Does the elevated autophagy flux in ABL KD and DDR1 KD cells depend on inactivation of ERK2? Can ectopic expression of active ERK reversed the increased WIPI1 and LC3 puncta in ABL KD and DDR1 KD cells?

We are grateful to the reviewer for raising this important point. We have addressed this issue using automated GFP-WIPI1 image analysis and can now show that the presence of active ERK2 in ABL1/2 KD and DDR1 KD settings significantly reduces the number of WIPI1 puncta per single cell.

This new data set is described in the revised result section (**page 8**) to read as follows (new text highlighted in yellow in the manuscript text):

*Consistent with this result, in both ABL1/2 KD and DDR1 settings, GFP-WIPI1 puncta counts decreased significantly when the ERK2-MEK1 fusion protein was overexpressed, as shown in automated single-cell image analysis (**Fig. 2i**).*

In fact, the effect was significant regarding GFP-WIPI1 cells, yet not evident in GFP-LC3 cells. We would like to point out in this context that the introduction of constitutively active ERK2 engages a network of diverse signaling pathways, including lysosomal signaling pathways associated with TORC1 activity. This scenario is different from settings of ERK2 deficiency. However, in our main subject of our study, WIPI1, we could indeed provide evidence of an association between ABL1/2 KD and DDR1 KD and ERK2 inactivation.

3. Since the authors claimed that Myc is the downstream factor of ERK inactivation, does overexpression of Myc reduce autophagy activity in WT, ABLKD or DDR1 KD cells?

Again, we would like to thank the reviewer for this important comment. We took up this critical point and considered how we can most directly check the MYC effect on WIPI1. Therefore we decided to focus on WIPI1 expression. As we have shown before, MYC binds to the WIPI1 promoter and represses expression (Fig. 3). This can be counteracted if we inhibit upstream ERK2 by administration of AZD0364 (Fig. 3c). We have now recapitulated this situation but have additionally used siControl or siABL1/2 settings in the presence or absence of transiently overexpressed MYC (**new Fig. S5c**). Here we found that overexpression of MYC significantly reduced the increase in AZD0364-mediated WIPI1 expression (left panel, siControl). If we carried out this experiment in the absence of ABL1/2 (right panel, siABL1/2) we found no significant increase in WIPI1 expression upon overexpression of MYC anymore.

This new data set is described in the revised result section (**page 9**) to read as follows (new text highlighted in yellow in the manuscript text):

*Furthermore, the AZD0364-mediated increase in WIPI1 gene expression was significantly reduced when c-MYC was simultaneously overexpressed in the siControl, and in siABL1/2 settings the AZD0364-mediated increase in WIPI1 expression was attenuated (**Fig. S5c**).*

4. Although the authors showed that Myc bound to the E-boxes and G9a sites in the WIPI1 promotor, further experiments, like luciferase assays, are needed to verify if the binding suppresses the transcription of WIPI1.

We thank the reviewer very much for this important suggestion, which we followed immediately. For this aim we employed the dual-luciferase reporter assay system (Promega) for the detection of both firefly and *Renilla* luciferase activities, whereby *Renilla* luciferase served as internal control for normalisation purposes. We used the pGL4 luciferase reporter system and cloned the WIPI1 promotor into pGL4.23[luc2/minP] upstream of a minimal promotor driving firefly luciferase. As a positive control we also cloned 5x canonical E-boxes into pGL4.23[luc2/minP]. We transfected U2OS cells, conducted luciferase reporter assays and report that indeed, the WIPI1 promotor suppressed luciferase activity, while in our positive control, 5x canonical E-boxes promoted luciferase activity, as expected. This result is in line with our suggestion that the sequence of the WIPI1 promotor that contains the three E-boxes (M+1, M11, M19) suppresses transcription.

Our new results are described in the revised result section (**page 10**) to read as follows (new text highlighted in yellow in the manuscript text):

*Based on these results, we performed luciferase reporter assays and confirmed that a partial WIPI1 promoter sequence, including E-boxes M19, M11, and M+1 (**Fig. S5g**), represses the minimal promoter activity driving firefly luciferase (**Fig. 3i, upper panel**). As*

*a positive control for MYC-mediated activation, we used a sequence with five canonical E-boxes (**Fig. S5h**), which activated minimal promoter activity on firefly luciferase (**Fig. 3i, lower panel**).*

5. Most experiments were done using WIPI1-expressing cells, which were quite artificial. WIPI1 KO should be involved to verify the role of WIPI1 at endogenous levels.

We fully agree with the reviewer's important point that additional experiments with WIPI KO cells would be crucial to investigate the role of WIPI1 as an enhancer in autophagy. Accordingly, we generated WIPI1 KO U2OS cells (**new Fig. S6b**) and are able to report that these new sets of results confirm our initial observation that WIPI1 serves as autophagy enhancer by impacting the autophagic flux:

- By quantitative Western blotting we show that WIPI1 overexpression elevates the autophagic flux in fed conditions, as scored by LC3 lipidation in both presence and absence of lysosomal inhibition (**new. Fig. 4b, Cas9 control**)
- WIPI1 KO cells display reduced basal autophagic flux since LC3-II level did not significantly increase upon lysosomal inhibition (**new Fig. S4b, WIPI1 KO**)
- When WII1 was reintroduced into WIPI1 KO cells, the autophagic flux was restored (**new Fig. S4b, WIPI1 KO**)

These new results are described in the revised result section (**page 11**) to read as follows (new text highlighted in yellow in the manuscript text):

*Consistent with this result, quantification of LC3 lipidation in fed conditions in the presence or absence of lysosomal inhibitor upon overexpression of 9E10-WIPI1 demonstrated significantly increased autophagic flux (**Fig. 4b, Cas9 control**). The autophagic flux was less pronounced in WIPI1 KO cells (**Fig. 4b; Fig. S6b, c**) as seen under fed conditions comparing minus and plus BafA1 settings, when overexpressing the empty 9E10 vector control (**Fig. 4b**). However, this deficiency was rescued by overexpression of 9E10-WIPI1 (**Fig. 4b**).*

Does WIPI1 KO inhibit the autophagy induction induced by siABL or siDDR1? Does WIPI1 KO affect WIPI2 puncta formation under starvation conditions?

We addressed this important question by using WIPI1 KO cells in both ABL KD and DDR1 KD settings in fed and starved conditions and conducted automated single cell imaging scoring for endogenous WIPI2 puncta per cell. We show in the **new Fig. 4d** that WIPI2 puncta are significantly reduced in WIPI1 KO cells in starved conditions in all settings.

We described this new result on **page 11** to read as follows (new text highlighted in yellow in the manuscript text):

*Likewise, we also found that the number of GFP-WIPI2B puncta cells increased significantly when mCherry-WIPI1 was overexpressed (**Fig. 4c**), and conversely, that the absence of WIPI1 decreased the number of endogenous WIPI2 puncta in both fed and starved conditions, as shown by automated single-cell image analysis (**Fig. 4d**).*

Does WIPI1 KO inhibit the TNT formation?

We fully agree with the reviewer that this is a key experiment in terms of investigating the relationship between the role of WIPI1 as an autophagy enhancer and transport of autophagic membranes by TNTs. We have expanded the use of WIPI1-KO cells to answer this intriguing and highly interesting question from the reviewer. Indeed, TNT formation was significantly reduced in WIPI1-KO settings, and WIPI1-KO cells did not respond with TNT formation as a starvation response either (**new Fig. 6f; S8c, d**).

This new set of data is described in the revised result section (**page 13**) to read as follows (new text highlighted in yellow in the manuscript text):

*Furthermore, we found that the presence or absence of WIPI1 affects TNT formation (**Fig. 6e, f**). Transient overexpression of GFP-WIPI1 cells significantly increased the number of TNTs (**Fig. 6e**), while WIPI1 deficiency significantly decreased the number of TNTs and starvation-induced TNT formation was significantly attenuated in U2OS cells (**Fig. 6f; S8c, d**).*

Minor points:

1. As ABL2 KD also significantly increased GFP-WIPI1 puncta, why ABL2 was not shown in the screen list as ABL1?

Indeed, shRNAs targeting ABL2 have not been included in the initial kinome screening presented here. To clarify this important point in the manuscript, we added the following sentence to **page 5** of the revised version of the manuscript:

It is noted here that shRNAs targeting ABL2 were absent in the initial kinome screen.

In some of the experiments, such as WIPI1 and LC3 puncta formation, the authors only showed the ABL1 or ABL2 single KD data, while in the other experiments, only ABL1 and ABL2 double KD were used. This inconsistency is quite confusing. The authors need to provide both single KD and double KD results side by side to show if ABL1 and ABL2 are indeed involved in autophagy regulation redundantly.

We are extremely grateful to the reviewer for bringing this point to our attention. We decided that after identifying the ABL1 candidate, we should also include ABL2 in subsequent studies, as ABL1 heterodimerizes with ABL2 and functions in concert with ABL2 (see below). The reason for the initial use of single ABL1-KD and ABL2-KD settings in Fig. 1d (GFP-WIPI1) and Fig. S2d (GFP-LC3), while subsequently using double ABL1/2-KD settings, has now been explained in the revised version more clearly (see below). We also changed the labelling in some figures to more clearly indicate that we used ABL1/2 KD throughout the study (horizontal instead of vertical labelling).

Based on the reviewer's important advice, we provide new automated imaging data on puncta numbers in single cells in the **new Fig. 1e (GFP-WIPI1)** and the **new Fig. S2f (GFP-LC3)**. We confirm that both ABL1 KD as well as in ABL2 KD settings provoked a significant increase of puncta numbers in single cells. Moreover, this effect was elevated in double ABL1/2 KD settings. Our new results are, in fact, in line with preliminary results we generated previously but had not included in the original submission. Furthermore, the new results led us to conduct follow-up experiments using double ABL1/2 KD settings, according to the study published by the Pendergast laboratory (referenced in the text below).

This new set of data is described and discussed in the revised result section (**page 5**) to read as follows (new text highlighted in yellow in the manuscript text):

*Since ABL1 and ABL2 have been shown to heterodimerize to regulate overlapping cellular processes but also to have distinguishable cellular functions [45], we compared single ABL1 KD and ABL2-KD settings to dual ABL1/2-KD settings in relation to autophagy (**Fig 1d**). Using automated single-cell analysis to assess GFP-WIPI1 puncta per cell, we confirmed that both ABL kinases are involved in autophagy regulation and that their influence on autophagy inhibition is stronger when both ABL1/2 are down-regulated (**Fig 1d**). Based on this result, which suggests a non-redundant effect of ABL1 and ABL2 on autophagy, we downregulated ABL1 and ABL2 together in follow-up experiments. This is consistent with the approach taken by Pendergast's study, which shows that the late stages of autophagy control in the course of lysosomal acquisition of hydrolytic enzymes depend on both ABL1 and ABL2 [31].*

2. In Figure 3h, why didn't G9A bind to the G9A-binding site?

G9a, a lysine methyltransferase, modifies chromatin by targeting the histone H3-lysine 9 (H3K9me1 and H3K9me2). In fact, since G9a performs this function by binding to DNA in

a sequence-independent manner, we did not necessarily expect to detect G9a itself by ChIP. However, we agree with the relevant publication that this site should be targeted by G9a, as we found it to be marked by H3K9me2, the product of G9a activity (Fig. 3h, lower panel). Within the stretch of DNA targeted by G9a, we had found a putative E-box, which we now refer to as M+1 instead of calling it the G9a site. In this context, we have detailed the positioning of the E-boxes in the WIPI1 promoter more clearly by improving the cartoon (**improved Fig. 3e**).

3. In Figure 4a, the label should be 0.1, 0.25 and 0.5.

We thank the reviewer for discovering this issue and have corrected this error accordingly.

Reviewer #3 (Remarks to the Author):

The work of Sporbeck et. al. characterizes several aspects of the function of WIPI1, a protein whose role in the regulation of autophagy is not clearly established. First, they show that WIPI1 expression is controlled by the ABL1-ERK-MYC signaling pathway. They propose that increased WIPI1 gene expression potentiates autophagy and that autophagic structures can travel between cells through tunneling nanotubes. Finally they show that ABL1 deficiency in *C. elegans* increases WIPI1 gene expression and prolongs lifespan in this model. These results are interesting and well supported by data, but some conclusions and functional connections are overstated with the available data and should be nuanced and discussed more clearly. Data presentation and statistical analysis should also be revised.

We are deeply grateful to the reviewer for the detailed evaluation and are very appreciative for the reviewer's positive words and constructive suggestions. Based on the insightful comments raised by the reviewer, we have completely and thoroughly revised the statistical analysis throughout the manuscript. We also provide more experimental reason for our statements, and stated limitations in the revised manuscript text. Below we detail each of the important points raised by the reviewer.

1. Many of the experiments rely on puncta counts. Sometimes the data is presented as "% of cells with puncta" and others as "number of puncta per cell". In the first case, does it mean % of cells with at least one puncta? or there is a threshold of puncta (or signal) from which a cell is considered "with puncta"? The data would be more consistent and comparable if the same measurement were shown. If this is not possible at least describe clearly what is shown, how the measurement was performed, as well as the number of experiments and/or the number of cells used per experiment.

We thank the reviewer for this important point. Accordingly, following the reviewer's advice, we now provide all necessary details to describe how the measurements were performed in more detail (see also below). In fact, using WIPI1, a reliable measure for e.g. high-throughput screening purposes or manual counting is to express the results as a percentage of cells exhibiting puncta, now uniformly indicated as "Puncta cells [%]" in the y-axis. In addition, we have detailed such analysis for single cell assessments, with the results expressed as the number of puncta per single cell, now uniformly referred to as "Puncta / cell" in the y-axis.

Furthermore, we provide all raw data, including the number of independent experiments performed as well as the number of cells used for each experiment in the 'Supplemental Data' Excel spreadsheet. In the revised version of our manuscript, and at the important request of the reviewer, we now include the number of independent experiments and the total number of cells analysed additionally in the figure legends.

Below we present an example of a figure legend (**new Fig. 1d**) that includes these important additions (new text in the revised version of the manuscript is highlighted in yellow).

*(**d**) Using CellProfiler-based single cell analysis of images acquired with automated confocal laser-scanning microscopy (LSM), the number of GFP-WIPI1 puncta per cell (threshold-based puncta segmentation) was determined with single knockdown of either ABL1 or ABL2, or double knockdown of ABL1/2 in fed conditions in the presence or absence of BafA1 as indicated. A two-way ANOVA with Tukey's multiple comparisons test was performed (up to 1241 cells from n=3 for each condition) and error bars show the mean±SD deviation.*

2. In this regard, the statistical tests used in puncta count experiments should be revised in each experiment. For example in Figure S2d: the significance of the analysis of GFP-LC3 puncta/cell has been analyzed using two-tailed heteroscedastic t test, which should be applied for data following a normal (parametric) distribution. Number of puncta per cell is likely to follow a non-normal distribution and therefore it would be appropriate to use non-parametric tests as the Kruskal -Wallis used for example in Fig 4c or FigS2n, where individual counts are displayed.

We absolutely agree with the reviewer´s judgement. Based on this exceptionally important point we completely revised and unified the statistical analysis for all measurements. Each data point is now displayed in each graph and statistical assessments are described on **page 32/33** in the revised version of our manuscript (new text highlighted in yellow in the manuscript text).

Statistical analysis
The statistical analysis methods used in this study are indicated in the legend for each subfigure. Statistical tests and graphs were performed with GraphPad Prism 9.2.0. Data are presented as mean ± standard deviation (SD) in bar graphs or scatter plots. Normal distribution of the data sets was assessed using normality tests and QQ plots. The following parametric tests were applied to data with normal distribution. Two-tailed heteroscedastic t-test (Welch's t test) was used for pairwise comparisons. One-way ANOVA was used for multiple comparisons with one factor, followed by the Dunnett's or Holm-Sidak post-hoc testing. Two-way ANOVA with Dunnett's or Tukey's post hoc test was used for multiple comparisons with more than one factor. When datasets were not normally distributed, they were logarithmically transformed (X log (Y) or x log (Y+1)) and when they fit a Gaussian distribution, parametric tests were run using the transformed data. In this case, the graphs presented in this study display original data with statistical analysis resulting from transformed data [102]. If Gaussian distribution was not achieved through logarithmic transformation, Kruskal-Wallis testing was conducted for multiple comparisons.
C. elegans lifespan assays were quantified by employing OASIS 2.
*The following P values were considered significant: * p<0.05; ** p<0.01; *** p<0.001.*

3. More in this regard, Fig. 4C is difficult to understand. It seems that % of cells with puncta is being measured. If so what are the individual points? these individual points range from 100% to 0% ??The axis should be puncta/cells?

We thank the reviewer very much for pointing out any ambiguities at this point. We have now detailed the description in the appropriate figure legend (**new Fig. 4e**) on **page 44** as follows (new text highlighted in yellow in the manuscript text).

*(**e**) G361 cells were transfected with plasmids encoding GFP or GFP-WIPI1 and were fed or starved for 3 h in the presence or absence of bafilomycin A1, followed by anti-WIPI2/AF546 immunofluorescence staining. Confocal LSM stacks were acquired, and the numbers of WIPI2 puncta-positive cells per acquired image (individual data points represent the result derived from each image) were counted (left panel: two-way ANOVA with Tukey's post-hoc test, mean±SD, up to 215 single cells from n=3 for each condition). Indicative of the presence of overexpressed GFP-WIPI1 are elongated, perinuclear*

autophagic membranes found to colocalize with WIPI2 (right panel: Scale bar: 5 µm, extended image presentation in Fig. S5b).

Also, for all figures where puncta/cell is displayed (for example Fig S2f), the individual data count (as in Fig S2n) would be more appropriate than bars, and the number of cells used for quantification should be indicated.

We also very much agree with the reviewer´s request. As stated in the above regarding our edited version of the statistical analysis, individual data points are presented in each graph. Additionally, the number of cells used are indicated in the figure legend and further detailed in the 'Supplemental Data' Excel sheet.

In the case of Fig S2f is not clear what n=6 means, only six cells analyzed?

We apologize for this lack of clarity. Following the reviewer's important question, we present informative extensions in the figure legend for Fig. S2f (**new Fig. S2g**) in the revised version of our manuscript as follows (new text highlighted in yellow in the manuscript text).

*(**g**) U2OS cells stably expressing RFP-GFP-LC3 were transfected with siRNAs targeting ABL1/2 (siABL1/2) (left panel) or DDR1 (siDDR1) (right panel) along with nontargeting control siRNAs (siControl) for 48 h. Prior to automated image acquisition and analysis using the InCell Analyzer 1000 (threshold-based puncta segmentation), cells were incubated in control medium (fed) or starved for 3 h. Autophagosomes were defined as RFP/GFP-positive LC3 puncta, and autolysosomes were defined as RFP-only LC3-positive puncta. Two-way ANOVA with Dunnett's post hoc test, mean±SD, up to 2690 (siControl for siABL1/2), 1493 (siABL1/2), 2778 (siControl for siDDR1) and 2843 (siDDR1) analysed cells from n=6 in each condition (fed or starved).*

4. Several experiments are shown that support the idea that increased expression of WIPI1 enhances autophagy. In my opinion, the data only support an increased number of puncta and the formation of large perinuclear autophagosomal structures, but no data on autophagy flux are provided. The presence of abnormal elongated structures is not evidence that autophagy is physiologically increased. The 4h experiment does not distinguish between the disassembly of this aberrant structure or the actual progression of autophagosomes. Perhaps the use of a WIPI1 KO cell line into which a WIPI1 expression plasmid can be introduced may provide clearer information on whether or not WIPI1 potentiates autophagy flux physiologically. At the very least, the data need to be discussed and the conclusions nuanced.

We are most grateful to both reviewer #3 and reviewer #2 for raising this important question. In the following please find our response provided in the above to address this point (reviewer #2, major point 5).

We fully agree with the reviewer's important point that additional experiments with WIPI KO cells would be crucial to investigate the role of WIPI1 as an enhancer in autophagy. Accordingly, we generated WIPI1 KO U2OS cells (**new Fig. S6b**) and are able to report that these new sets of results confirm our initial observation that WIPI1 serves as autophagy enhancer by impacting the autophagic flux:

- By quantitative Western blotting we show that WIPI1 overexpression elevates the autophagic flux in fed conditions, as scored by LC3 lipidation in both presence and absence of lysosomal inhibition (**new. Fig. 4b, Cas9 control**)
- WIPI1 KO cells display reduced basal autophagic flux since LC3-II level did not significantly increase upon lysosomal inhibition (**new Fig. S4b, WIPI1 KO**)
- When WII1 was reintroduced into WIPI1 KO cells, the autophagic flux was restored (**new Fig. S4b, WIPI1 KO**)

These new results are described in the revised result section (**page 11**) to read as follows (new text highlighted in yellow in the manuscript text):

*Consistent with this result, quantification of LC3 lipidation in fed conditions in the presence or absence of lysosomal inhibitor upon overexpression of 9E10-WIPI1 demonstrated significantly increased autophagic flux (**Fig. 4b, Cas9 control**). The autophagic flux was less pronounced in WIPI1 KO cells (**Fig. 4b; Fig. S6b, c**) as seen under fed conditions comparing minus and plus BafA1 settings, when overexpressing the empty 9E10 vector control (**Fig. 4b**). However, this deficiency was rescued by overexpression of 9E10-WIPI1 (**Fig. 4b**).*

5. The authors attempt to connect the function of WIPI1 to tunneling nanotube (TNT) activity. It is stated in the abstract that "increased expression of the WIPI1 gene enhances the formation of autophagic membranes capable of migrating through tunneling nanotubes to neighboring cells with low autophagic activity". In my opinion this sentence is misleading as it leads to the idea that WIPI1 is required to generate a specific structures capable of trafficking between cells. These aspects may only be connected phenomenologically (just because they coincide in a cell). Many different types of vesicles, including autophagosomes, are expected to be able to travel across TNTs. To support a specific functional connection of this phenomenon to WIPI1 function, a number of questions need to be addressed: for example, does the presence of TNTs depend on WIPI1?

We thank all three reviewers who raised the question about the relationship between WIPI1, TNT formation and TNT transfer. We have devoted ourselves with enthusiasm to this point and in the following, please find our text on this issue taken from responding to reviewer #1 (major point 5).

We can only underline that the reviewer has formulated a very important remark, for which we thank sincerely. Accordingly, we have transfected U2OS cells with increasing WIPI1 plasmid concentrations (0.1, 0.25 μg) and quantified the numbers of TNTs formed (TNT index). While the lower concentration (0.1 μg) of transfected WIPI1 plasmid had no effect on THT formation, indeed, we observed a significant increase of TNTs when we introduced 0.25 μg of the WIPI1 plasmid (**new Fig. 6e**). In line with this finding, we further employed WIPI1 KO cells that we generated during this revision (see also later in the text), and found that in the absence of WIPI1, TNT formation was significantly reduced (**new Fig. 6f**). Combined these results suggest that WIPI1 impacts TNT formation.

Motivated by this result, and based on the reviewer's important request, we transfected a coculture of donor cells expressing GFP-LC3 and recipient cells with NLS-mScarlet marked nuclei with increasing WIPI1 plasmid concentrations (0.2, 0.4, 0.6 μg). We then counted the number of recipient cells harbouring GFP-LC3 puncta and observed that increasing WIPI1 plasmid concentrations (0.6 μg) promoted autophagosome transfer (**new Fig. 6l**). This observation now links enhanced WIPI1 expression with enhanced autophagic flux (see also later in the text) and with an increase in transfer of autophagic membranes through TNTs.

These results are described in the revised result section (**page 13**) to read as follows (new text highlighted in yellow in the manuscript text):

*Furthermore, we found that the presence or absence of WIPI1 affects TNT formation (**Fig. 6e, f**). Transient overexpression of GFP-WIPI1 cells significantly increased the number of TNTs (**Fig. 6e**), while WIPI1 deficiency significantly decreased the number of TNTs and starvation-induced TNT formation was significantly attenuated in U2OS cells (**Fig. 6f; S8c, d**).*

*In this context, it is conceivable that an enhancing role of WIPI1 in the formation of autophagic membranes should therefore also influence the extent of autophagic membrane transport by TNTs. This assumption turned out to be relevant since the transfer of GFP-LC3 positive autophagosomes was significantly increased when WIPI1 was overexpressed (**Fig. 6l, m**).*

Is there preferential transport of WIPI1-containing structures? The authors should clearly discuss the limits of this observation and qualify their conclusions.

The reviewer is absolutely correct that this is a new area with several unanswered questions. Although we now provide more data on the relationships between WIPI1 and TNT transfer (see above), e.g. the specificity of the transport is unknown. Following the reviewer's important comment, we have included (highlighted in yellow) the following statement in the discussion section on **page 17**.

Although this finding now warrants the collection of molecular details in future studies, important for a mechanistic understanding of TNT formation and selectivity in autophagic cargo transport by TNTs, it underscores that autophagic membrane transport through TNTs should be a vital mechanism by which cells with high autophagic activity may rescue cells with low autophagic activity.

6. In the same line: Figure 6f. The longevity experiments have been performed in C. elegans, not in mammalian models or humans. Therefore, the human symbol should be changed to that of a worm.

We are most grateful to the reviewer for this important point to our attention. Accordingly, now the symbol displays *C. elegans* in the **new Fig. 8c**.

REVIEWERS' COMMENTS:

Reviewer #1 (Remarks to the Author):

The authors responded to all of my concerns I raised for the original manuscript in a satisfactory manner.

Reviewer #2 (Remarks to the Author):

The revision has fully addressed my concerns. I would recommend publication of the manuscript.

Reviewer #3 (Remarks to the Author):

The authors responded satisfactorily to all my questions. Therefore, I recommend acceptance of the manuscript.
